# Rethinking concrete durability for low-carbon concretes through climate-informed corrosion modelling

**DOI:** 10.1038/s41467-026-76916-3

**Published:** 2026-08-21

**Authors:** Cristhiana Albert, Thilo Schmid, Zhidong Zhang, Ueli Angst

**Affiliations:** Institute for Building Materials, ETH Zurich, Zurich, Switzerland

**Keywords:** Civil engineering, Ceramics, Metals and alloys

## Abstract

Decarbonizing concrete is essential for meeting global climate targets. Many effective strategies to reduce greenhouse gas emissions result in lower concrete alkalinity, which has traditionally been viewed as a durability concern, namely promoting steel corrosion in concrete. This prevailing durability paradigm – firmly embedded in engineering mindsets and industry standards – hinders the practical implementation and thus the realization of the full potential of low-carbon concretes. To resolve this situation, we propose a conceptual framework that offers a perspective on ensuring the durability of reinforced concrete, while tolerating reduced concrete alkalinity. Our approach is inspired by empirical observations and recent scientific insights that consistently pinpointed the decisive role of moisture in concrete durability. We leverage advancements in moisture transport modeling in porous media and corrosion science to predict the time-evolution of the microclimate and corrosion kinetics at the steel-concrete interface. By drawing on time-resolved meteorological data from four examples in Europe, Asia and South America, we showcase the approach, underlining the critical role of local climate for predicting the durability performance of concrete. This work establishes the basis for developing test methods, standards, and predictive tools to promote eco-friendly concrete and evaluate how climate change impacts their durability for generations to come.

## Abstract (web summary)

The work develops a modeling framework that integrates time-resolved climate data, moisture transport in low-carbon concrete, and quantitative steel corrosion kinetics to assess durability and service life across different materials and climates.

## Introduction

Accounting for up to 8% of the anthropogenic global CO_2_ emissions and thereby significantly contributing to climate change^1^, the cement industry faces an imperative need to decrease this metric^2–4^. Cement-related greenhouse gas emissions are associated with (i) the energy required for processing raw materials—mainly limestone and clay—at high-temperature kilns (1450 °C), (ii) the CO_2_ released as a result of reactions that yield the calcium-rich clinker, the main component of traditional Portland cement, and (iii) minor emissions associated with material grinding. In the last decades, a widely adopted strategy to decrease CO_2_ emissions has been the partial replacement of clinker with raw materials (such as limestone, calcined clays, and natural pozzolans) or industrial by-products (fly ash, ground granulated blast furnace slag, combustion ashes from biomass and waste)^5–7^. Such low-clinker cements can produce concrete with properties comparable to traditional cements, and in some respects, even superior, such as higher long-term strength and decreased porosity^8–10^. However, a major concern with cements that have a low clinker content is their limited resistance to carbonation^11,12^, which is widely regarded as a key durability issue under the prevailing paradigm that associates carbonation with steel corrosion in concrete^13^.

Carbonation is a natural process characterized by the ingress of CO_2_ from the air into the concrete matrix, followed by the reaction of CO_2_ with primarily the calcium-rich phases of concrete to form CaCO_3_^12^. The carbonation process affects both the phase assemblage and the pore structure of concrete, generally refining the pore structure for calcium-rich cements and coarsening it for low calcium cements^14,15^. Moreover, the alterations in solid phases affect the chemistry of the liquid phase contained in the pores, reducing its pH from the initially highly alkaline level of Portland-cement-based concrete (pH > 12.5) to near-neutral pH levels of carbonated concrete (pHs ~ 7.5–9.5)^16,17^. As a result, the pH decrease destabilizes the passive film^18^, a thin oxide layer that forms on steel under alkaline conditions at the steel-concrete interface^19^. This depassivation is potentially critical, since the formation of the passive film under alkaline conditions in traditional concrete is the key reason why low-cost carbon steel can be widely used as reinforcement in concrete construction.

Upon loss of the passive film, steel corrosion can occur^18,20^, a process in which ferrous ions are released at the metal surface in an anodic reaction, balanced by a cathodic reaction, generally oxygen reduction. In porous media such as concrete, the anodic iron dissolution is followed by complex and interrelated reactive transport processes^13,21,22^, leading to the formation and transformation of solid phases that precipitate in the pore network of concrete. These processes can thus gradually generate expansive stresses, eventually leading to concrete cracking and spalling, which represent critical limit states for the service life of an engineering structure. Beyond its implications for safety, corrosion-related structural deterioration leads to considerable economic burdens for society^23–25^. A significant portion of these costs arises from the need to repair and maintain degrading infrastructure, which is a grand challenge in all industrialized countries^26,27^. Improved understanding of the underlying degradation mechanisms can contribute to extending the service life of structures, thereby not only mitigating the economic impact of corrosion, but also reducing or postponing repair interventions, which decreases the environmental footprint of the construction sector by limiting the consumption of raw materials (including critical resources such as sand^28^) and by decreasing greenhouse gas emissions associated with construction activities.

Given the specific degradation mechanism described above, the field has traditionally treated carbonation-induced corrosion as a distinct deterioration process, separate from other mechanisms such as chloride-induced corrosion. Consistent with this established distinction, the present study focuses exclusively on carbonation-induced corrosion, with particular emphasis on the rate at which corrosion develops. In fact, the rate at which the detrimental corrosion process occurs in practice can vary significantly depending on the actual service conditions. Both practical experience^20^ and scientific studies^13,20,21,29–32^ have consistently shown that, under dry conditions, steel corrosion rates and hence damage accumulation are negligibly slow. On the other hand, high corrosion rates can develop at elevated moisture states of the concrete matrix. Recent studies have uncovered the dual role of moisture in reinforced concrete: the moisture-porous medium interaction controls the metal surface available for electrochemical reactions and simultaneously influences the saturation degree of the matrix, thereby affecting the reactive transport coupled to the electrochemical interfacial processes^21,32,33^. Moreover, a study^34^ combining electrochemical monitoring of steel corrosion with neutron radiography to track the ingress of a moisture front by capillary suction in a carbonated cementitious matrix has emphasized the importance of the dynamic behavior of moisture: Once the moisture front reached the steel, corrosion was found to be activated quasi-instantaneously. This dynamic moisture-corrosion interaction in concrete is important because structures are rarely exposed to equilibrium moisture conditions; instead, they are typically subjected to time-variable exposure, such as wet-dry cyclic conditions.

These insights on the dominant role of moisture in controlling steel corrosion in carbonated concrete offer a novel route to ensuring long-term durability. By focusing on moisture transport in concrete and related corrosion, we can relax the reliance on the traditional approach of preventing carbonation from reaching the steel. Moving beyond this traditional approach is urgently needed to resolve the goal conflict between reducing greenhouse gas emissions from cement and ensuring durability, where, in the prevailing paradigm, the latter is often equated with preventing carbonation. This conflict, known as the “carbonation dilemma”, limits the full exploitation of low-carbon concretes. For example, several countries do not allow the use of certain low-clinker cements in aggressive exposure conditions (Germany^35,36^, United Kingdom^37^, Belgium^38,39^, Spain^40^, India^41^), impose restrictions on the carbonation resistance (Switzerland^42,43^, Belgium^38,39^, Brazil^44–46^, United States^47–49^, Japan^50,51^), or require larger cover depths or lower water-to-binder ratios for low-clinker concretes compared to traditional concrete (Germany^35,36^, Spain^40^, Portugal^52,53^, China^54,55^, Norway^56^). Ultimately, these strategies lead to higher binder consumption and, as a result, to CO_2_ emissions that are potentially greater than the levels initially envisioned during the development of low-carbon cements.

State-of-the art approaches for service life design of reinforced concrete structures include prescriptive standards – such as the codes in Europe, China, and India –, where the design life is ensured by specifying material or geometrical requirements according to the environmental exposure class of the structure (*deemed-to-satisfy approach*)^57^. On the other hand, *performance-based approaches* have become more widespread, such as in Belgium and Switzerland, but these standards^38,39,42,43^ are still dominated by the traditional paradigm, namely to avoid carbonation of the concrete at the steel depth. Alternatively, some countries (e.g., the United States^49^, Brazil^45^) permit explicit consideration of the phase following carbonation-induced depassivation of the steel by modeling corrosion, though guidance on methodology and limit state criteria remains limited. Spain and Japan provide empirical equations for the corrosion phase, but the somewhat limited transparency concerning the origin and calibration may restrict their applicability to new cementitious systems and their ability to capture the complex exposure conditions encountered in different geographic locations globally. A detailed overview of the state-of-the-art approaches in different international standards is provided in Supplementary Note 1.

A common shortcoming of these approaches lies in the overly simplified description of exposure conditions. For instance, the European standard distinguishes exposure classes such as dry (XC1), wet and rarely dry (XC2), moderate humidity (XC3), or cyclic wet and dry (XC4). These exposure classes are defined qualitatively, although efforts have been made to describe them in quantitative terms. For example, the average long-term annual relative humidity (RH) and the average annual time of wetness (ToW) were analyzed for selected European countries in ref. ^57^. While for field studies on carbonation of concrete, time-resolved meteorological information is often lacking^58^, there are a few examples where detailed data such as on RH and temperature were considered^59–61^. However, the exposure classes in standards remain qualitative and fall short of capturing the diverse climatic conditions across different geographic regions, especially the rate at which steel corrosion in carbonated concrete progresses. This poses a challenge, as a wide range of climatic conditions can, for instance, fall within the range of “cyclic wet and dry” (XC4), yet these conditions can lead to markedly different durability performances. While empirical evidence from engineering practice points to these pronounced moisture and exposure environment-related effects on corrosion^20^, limited quantitative, conceptual modeling frameworks currently exist to capture them and thus to allow for a holistic service life assessment of modern, low-carbon concrete.

**Fig. 1:**
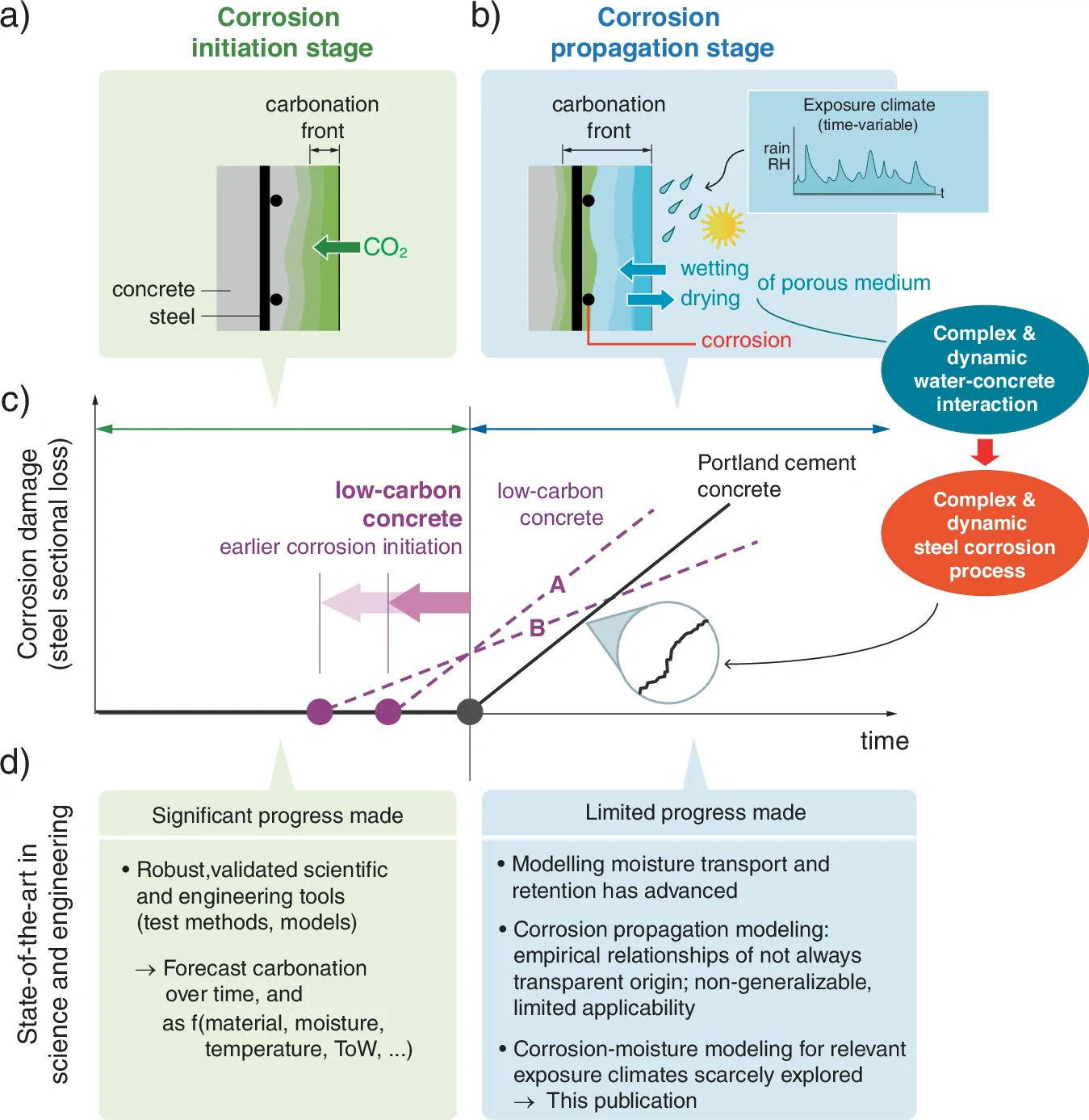
Carbonation-induced steel corrosion in concrete and its effect on service life. Schematic illustration of the service life of a reinforced concrete structure (**c**), following the classical separation into a corrosion initiation phase, in which the ingress of CO_2_ and its reaction with the cementitious phases are the processes controlling the penetration of the carbonation front (**a**), and a corrosion propagation phase (**b**), in which the complex and dynamic water-concrete interaction, governed by the time-variable exposure climate and the concrete material properties, ultimately control the electrochemical corrosion processes at the embedded steel. Figure **c** schematically illustrates the effect of eco-friendly concrete that tends to carbonate faster than traditional Portland cement concrete, potentially leading to an overall shorter service life (letter A), or where the corrosion propagation phase may be beneficial (letter B) in the long term. Figure **d** summarizes the state-of-the-art knowledge in science and engineering with respect to these two phases.

In service life modeling of reinforced concrete, significant progress and validation have been made over the last decades in modeling the carbonation process of concrete, the so-called corrosion initiation phase (Fig. 1)^62,63^. This can be attributed to the extensive research efforts into understanding the carbonation process of concrete^12^ as well as developing and validating quantitative models, such as the DuraCrete model^62,63^. Such models are capable of taking into account detailed climatic factors such as time of wetness, RH of the air, and temperature^62,63^. Additionally, standardized and well-established accelerated laboratory test methods to assess the carbonation resistance of concrete exist, and they have been related to natural, real-life carbonation conditions^64–66^. Hence, the current state-of-the-art (Fig. 1d) allows for reasonably reliable predictions of the carbonation progress of concrete, both traditional and modern, eco-friendly concretes.

However, models to forecast and evaluate the subsequent corrosion propagation phase (Fig. 1b, d) remain lacking, especially models that can account for the actual exposure climate and the complex temporo-spatial moisture effects. For example, the DuraCrete model^62,63^ proposes empirical relationships to calculate corrosion rates based on electrical concrete resistivity (an indirect and approximate indicator for which moisture constitutes one of multiple influencing parameters). Kölio et al.^67,68^ proposed a linear regression model to predict the corrosion rate of steel based on weather indicators, considering monthly averages rather than explicitly modeling the moisture ingress in concrete based on the actual wetting/drying exposure and the concrete’s sorptivity properties. Korec et al.^69^ modeled moisture ingress in concrete and linked it to corrosion under strongly idealized exposure conditions, not reflecting real climatic conditions.

This absence of dedicated models for the corrosion propagation phase in carbonated concrete, capable of accounting for dynamic moisture-corrosion interactions, is particularly critical given the growing relevance of the propagation phase in low-carbon concretes that have shortened corrosion initiation phases (Fig. 1c). This underscores the need for dedicated predictive tools. Moisture transport modeling in concrete has made substantial progress over the last decades^70–73^, which offers opportunities to address the aforementioned challenge of integrating temporo-spatial moisture distribution in concrete with corrosion science. This is urgently needed because current approaches for predicting the corrosion propagation phase in service life design for concrete in carbonation exposure (see Supplementary Note 1) are generally empirical and associated with somewhat limited transparency concerning the origin and calibration, which may restrict their applicability to new cementitious systems and their ability to capture the complex exposure conditions encountered in different geographic locations globally. While literature data is available for instantaneous corrosion rates of steel embedded in carbonated concrete, e.g. as a function of the actual moisture state^21^—or rather the ambient exposure RH (see Supplementary Note 10) –, limited attempts have been made to integrate the dynamic and complex exposure conditions, e.g. rain patterns, characteristic of specific climatic (and meteorological) conditions in order to describe the time-variable corrosion rate evolution within carbonated concrete. In this context, this contribution proposes a model framework allowing for a more detailed evaluation of the role of the corrosion propagation phase than with state-of-the-art approaches.

## The proposed concept

In this work, we address the gap described above and propose an approach to evaluate the corrosion propagation phase, thereby expanding existing models for the initiation phase and providing a complete treatment of the service life of concrete structures. Our approach combines three key components:

- Meteorological data from weather stations, in particular precipitation patterns, provide highly time-resolved information on the duration of wet-dry cycles and their seasonal variations.
- Advanced moisture transport modeling in carbonated concrete, considering the specific, time-resolved climatic conditions of different geographic locations. This modeling explicitly incorporates the material properties of the carbonated cementitious systems under investigation, including capillary absorption and sorption isotherms, to accurately simulate moisture dynamics.
- Quantitative relationships between the degree of water saturation of the cementitious matrix and the corrosion rate of steel provide the critical link between concrete moisture dynamics and durability performance.

Note that the proposed approach here is not intended for direct application in practice. Instead, this detailed and fundamentally based concept, incorporating state-of-the-art knowledge, is meant to serve as a guiding concept for future research and development engineering.

To illustrate the proposed concept, we selected three different concrete types (“Methods” section) and four different geographic locations with different climates (Table 1). These geographic locations were selected to span a broad range of climatic conditions, including a semi-arid, continental climate (Huailai, Asia), a continental one with moderate rainfall throughout the year (Zurich, Europe), a rainforest-dominated one characterized with high rainfall and constant moisture (Manaus, South America), and finally a coastal one with humid maritime climate (Bergen, one of the rainiest cities in Europe). Table 1 shows clear differences in relative humidity (RH) and annual precipitation for the different locations. From publicly available meteorological data^74–77^, the temporal characteristics of precipitation were further quantified by the weekly Precipitation Concentration Index (PCI)^78^, a common parameter in climate research, and by analyzing the distribution of wetting phase durations, with the 90^th^ percentiles provided in Table 1. Supplementary Note 2 gives a more detailed statistical analysis of the different exposure regimes. It is worth emphasizing that, despite the significant differences in meteorological conditions, unsheltered structural components would in all four locations fall under exposure class XC4 “cyclic wet and dry” in accordance with European standards.

**Table 1:** Precipitation and Relative Humidity (RH) data for the four geographic locations considered, all characterized by exposure class XC4 (cyclic wet and dry) according to European standard EN 206

Location	Climate	Avg. RH (%)	Total annual precipitation (mm)	Days with >1 mm of precipitation	Weekly PCI	90^th^ percentile of rain event durations (h)
Huailai, China	Semi-arid, continental	54.7	234	25	34.32	2
Zurich, Switzerland	Continental with moderate rainfall throughout the year	76.1	1109	133	4.36	1.3
Manaus, Brazil	Rainforest, high rainfall and constant moisture throughout the year	73.8	2448	152	4.01	4
Bergen, Norway	Coastal with one of the highest rainfall amounts in Europe	77.3	2806	208	3.23	10

The meteorological data were selected for a single year in each location, ensuring that data availability in the reported data was high. The rain event duration shown in the last column corresponds to the duration for which 90% of all rain events are shorter. See Supplementary Note 2 for more details.

## Results and discussion

### Climate-informed moisture transport and corrosion modeling

The water ingress into the concrete was simulated by an advanced and validated moisture transport model, considering both liquid and water vapor transport^70^ and material properties experimentally determined for carbonated concrete (see “Methods” section, Supplementary Notes 3–5 for details). To account for the actual climatic conditions for the different geographic locations, the RH derived from the meteorological data was imposed as a time-variable boundary condition (with a 1 h resolution). Additionally, recognizing the influence of prior exposure history to specific climatic conditions, the initial moisture content in the concrete was defined uniquely for each geographic location. These starting conditions were determined by assuming the concrete had internally reached equilibrium with the local climate, based on simulations spanning several years to capture the internal equilibration process.

**Fig. 2:**
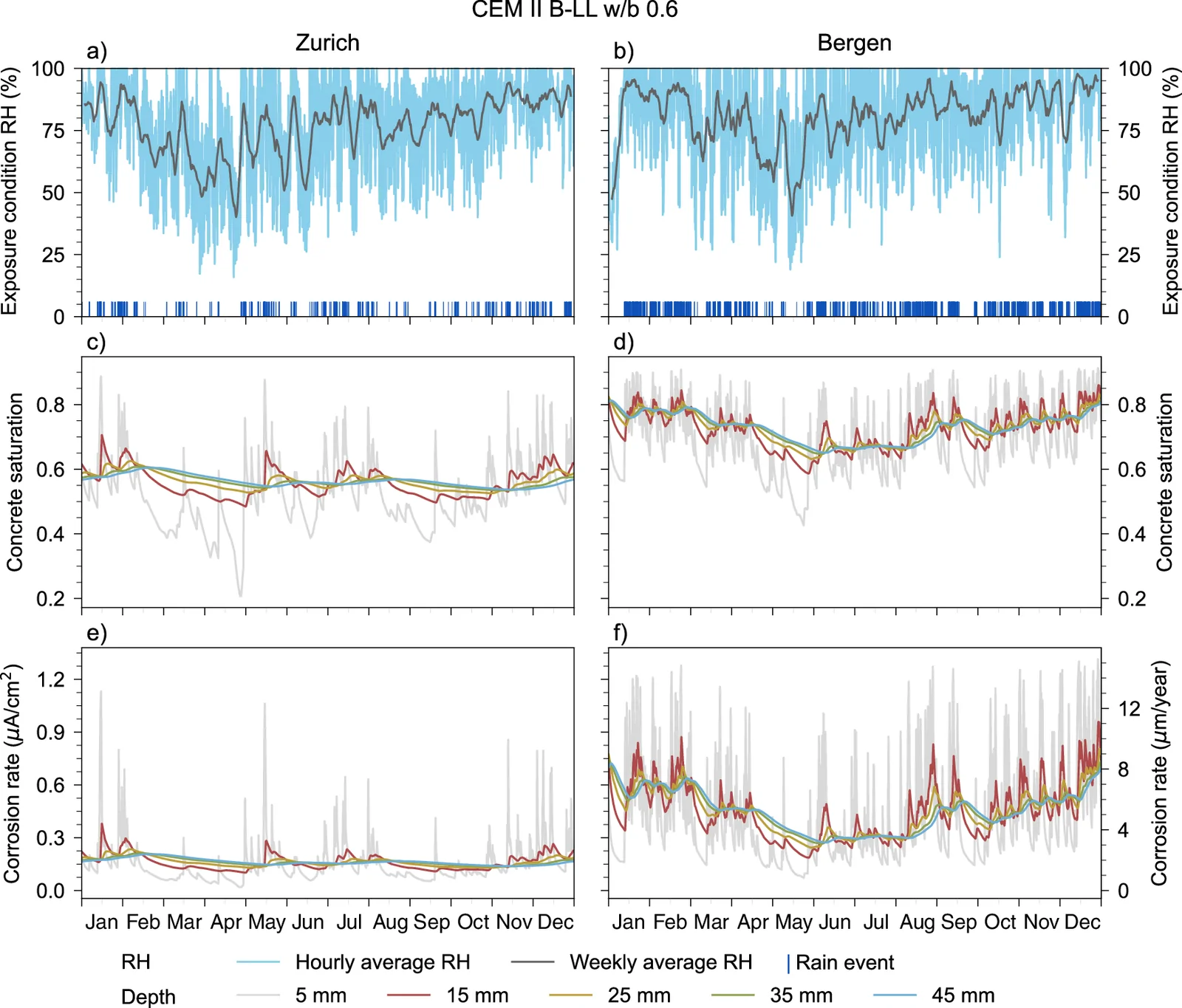
Forecasting the time-variable moisture state and steel corrosion rate in carbonated concrete as a function of exposure climate. Here, the case CEM II B-LL with w/b 0.6 is shown for exposure climates Zurich (Switzerland) and Bergen (Norway); all other considered cases are displayed in Supplementary Figs. 10–21. In detail, the figure displays RH variations and rain events in **a** Zurich and **b** Bergen; the modeled concrete saturation at specific depth (*x*) in **c** Zurich and **d** Bergen, and the resulting instantaneous corrosion rates of steel embedded at different depths (*x*) in **e** Zurich and **f** Bergen. Water ingress was simulated using an advanced two-phase moisture transport model accounting for both liquid and water vapor transport^70^ and experimentally derived material parameters given in Supplementary Notes 3–5. Site-specific RH derived from meteorological data was applied as a time-dependent boundary condition (1 h resolution). Conversion of the steel-concrete interfacial moisture state to the instantaneous corrosion rate was done based on state-of-the-art quantitative relationships. For more details, see “Methods”. Source data are provided as a Source Data file.

Figure 2a, b shows examples of the time-resolved variations in exposure conditions for the case of Zurich and Bergen (see Supplementary Note 7 for complete results of all scenarios). The corresponding variations in moisture state in the concrete are depicted in Fig. 2c, d, illustrating the pronounced effect on depth from the exposed concrete surface (*x*). The results further illustrate the well-known phenomenon that upon wet phases, the moisture state in the concrete, especially at shallow depths like 5–15 mm, rises rapidly, while it decreases gradually during the subsequent dry stage. For the considered cases, beyond a depth of 35 mm, the concrete moisture state exhibits minimal daily fluctuations, instead remaining largely around the average, with slight variations over timescales of months. Figure 2e, f shows the corresponding instantaneous corrosion rates, essentially following a similar trend, with somewhat more pronounced peaks, which can be explained by the non-linear moisture-corrosion rate relationship (Supplementary Note 8).

**Fig. 3:**
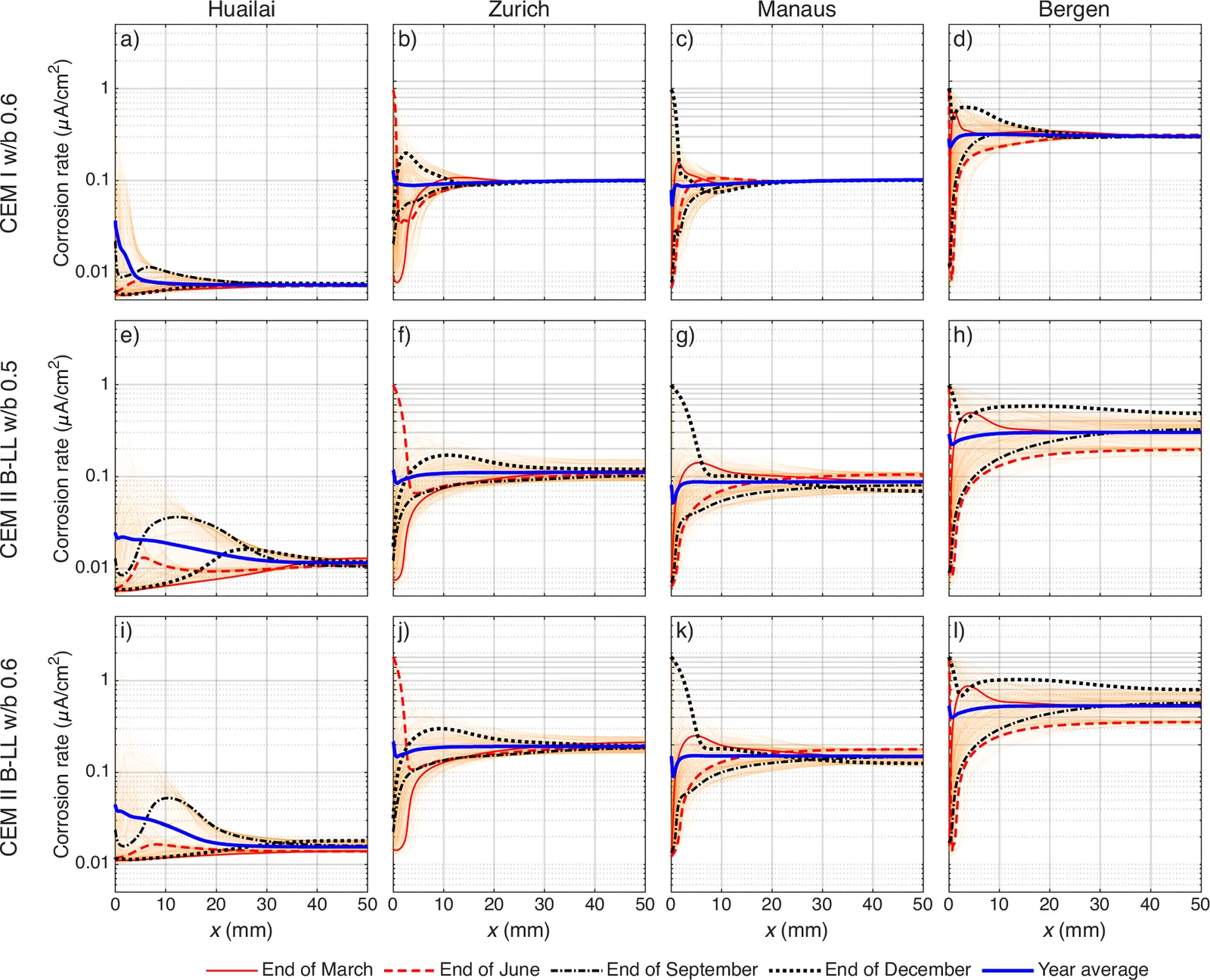
Instantaneous corrosion rate throughout a representative year as a function of depth (*x*) in the three considered carbonated concretes exposed to the four considered climatic conditions. Specifically, concrete CEM I with w/b 0.6 for exposure climates in Huailai (**a**), Zurich (**b**), Manaus (**c**), Bergen (**d**); concrete CEM II B-LL with w/b 0.5 for exposure climates in Huailai (**e**), Zurich (**f**), Manaus (**g**), Bergen (**h**); concrete CEM II B-LL with w/b 0.6 for exposure climates in Huailai (**i**), Zurich (**j**), Manaus (**k**), Bergen (**l**). Note the logarithmic y-axis to capture the large variation in corrosion rates. Each thin line shows the corrosion rate on a specific day throughout the year; the red and black thick lines show corrosion rates at the end of a quarter; the blue thick line stands for the average corrosion rate of the year. Source data are provided as a Source Data file.

Figure 3 consolidates the time-resolved simulation results of the predicted corrosion rate over one full year for all three concretes and four exposure climates considered, with each individual line in each chart representing the daily-based instantaneous corrosion rate distribution. Seasonal variations associated with yearly rainfall distribution are highlighted with the dashed lines, while the thick blue line shows the yearly average corrosion rate of steel. For all examined cases, the highest corrosion rates occur at shallow depths, where environmental fluctuations have the strongest influence. Moreover, in this near-surface region, the instantaneous corrosion rates are observed to scatter significantly, which reflects the pronounced temporal variability in exposure conditions (wetting and drying). With increasing depth *x*, the variability in corrosion rate declines, stabilizing at a certain depth. This depth, beyond which the corrosion rate remains largely unaffected by external climate variations, differs significantly for the different concrete types and geographic locations. Additionally, the level at which the instantaneous corrosion rate stabilizes in this interior part differs significantly for the different geographic locations and the related climates. In fact, the climatic exposure conditions have a significantly greater impact than the material itself, even though a broad spectrum of materials was examined here.

As expected, Fig. 3 shows that, independent of the geographical location, increased corrosion rates occur in more porous concretes. This can be seen from comparing w/b 0.5 and 0.6 for CEM II B-LL, where a higher w/b corresponds to a higher porosity, or from comparing CEM I with CEM II B-LL at the same w/b 0.6, where CEM I is expected to form a denser microstructure in carbonated concrete. These increased corrosion rates at higher porosity are attributed to the (i) faster moisture transport within the concrete cover, leading to extended periods of critically high moisture state at the steel surface, and (ii) intrinsically higher corrosion rates due to the dual role of moisture and porosity on the electrochemical behavior of steel in porous media^21,33^.

**Fig. 4:**
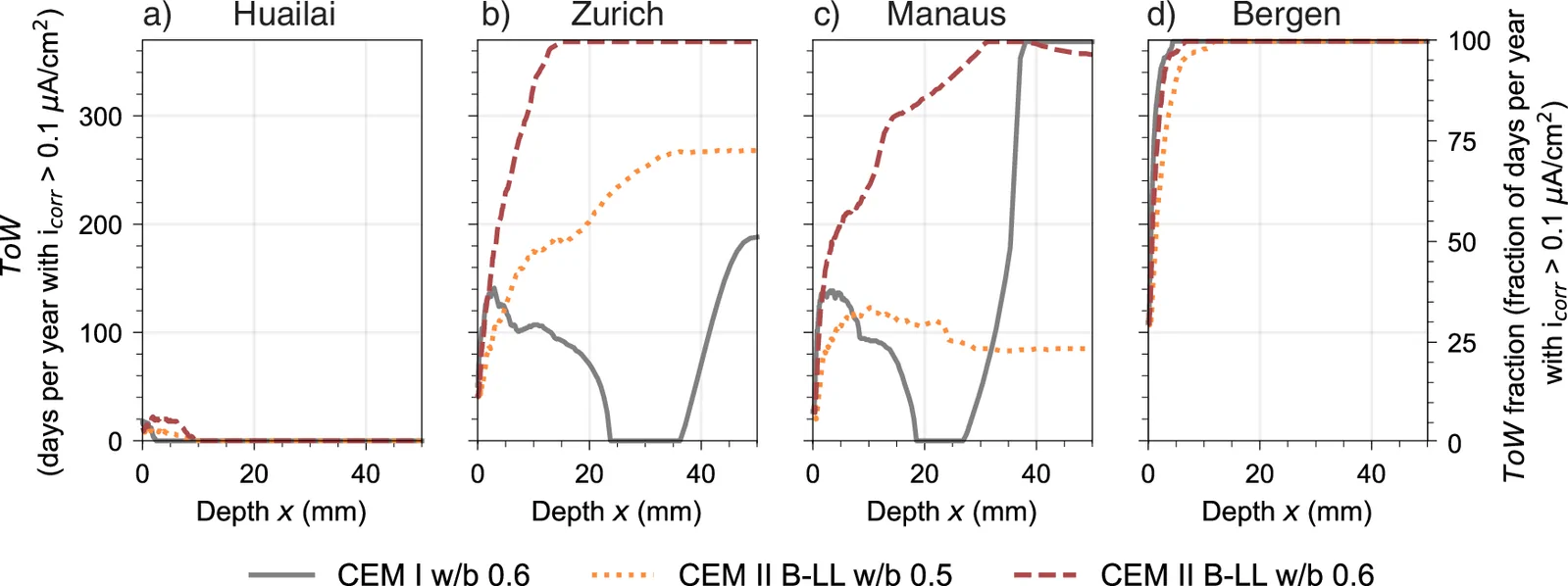
Time of wetness (ToW) as a function of depth (*x*), illustrating the complex, non-linear behavior of different concretes in different geographic locations, resulting from the combined effects of material properties and time-variable exposure conditions. Here, for the purpose of illustration, ToW was defined as the number of days in a year with concrete saturation leading to instantaneous corrosion rates >0.1 μA/cm^2^. Panels display the different exposure climates Huailai (**a**), Zurich (**b**), Manaus (**c**), and Bergen (**d**). Source data are provided as a Source Data file.

The duration during which the moisture state within the concrete is sufficiently high to promote non-negligible electrochemical steel corrosion may be evaluated in terms of the time of wetness (ToW), which is a concept widely used in the field of atmospheric corrosion^79^. Here, for the purpose of illustration, we consider the figure 0.1 μA/cm^2^ as an established threshold distinguishing negligible from non-negligible instantaneous corrosion rate^80^. Figures 3 and 4 shows the significant differences in ToW between the semi-arid, continental climate in Hualiai and the coastal, rainy climate in Bergen. The examples of Zurich and Manaus, on the other hand, reveal the pronounced influence of the material properties of the concrete.

While the concept of ToW may be useful for an initial classification of the corrosiveness of a certain material-climate combination, the examples in Fig. 4, especially the case of CEM I in Zurich and Manaus, highlight the limitation in applying this concept to the case of steel corrosion in cementitious media. The main problem with the ToW concept is that it treats corrosion as a binary, on-off state, but the actual corrosion pattern is more complex. As shown in our examples (Figs. 3 and 4), the instantaneous corrosion rate can vary dramatically even when the conditions are above the wetness threshold. Since for steel corrosion in porous media, with increasing moisture content, the instantaneous corrosion rate increases exponentially^21^ rather than gradually, a binary metric like ToW cannot capture the underlying processes adequately and therefore fails to describe corrosion behavior in porous systems. This is particularly evident in the CEM I examples from Zurich and Manaus. In these cases, the conditions are often hovering around the 0.1 μA/cm^2^ threshold value, which means the material is frequently transitioning between a wet and dry state. This frequent transitioning leads to the complex, non-linear relationship between ToW and depth that is shown in Fig. 4.

**Fig. 5:**
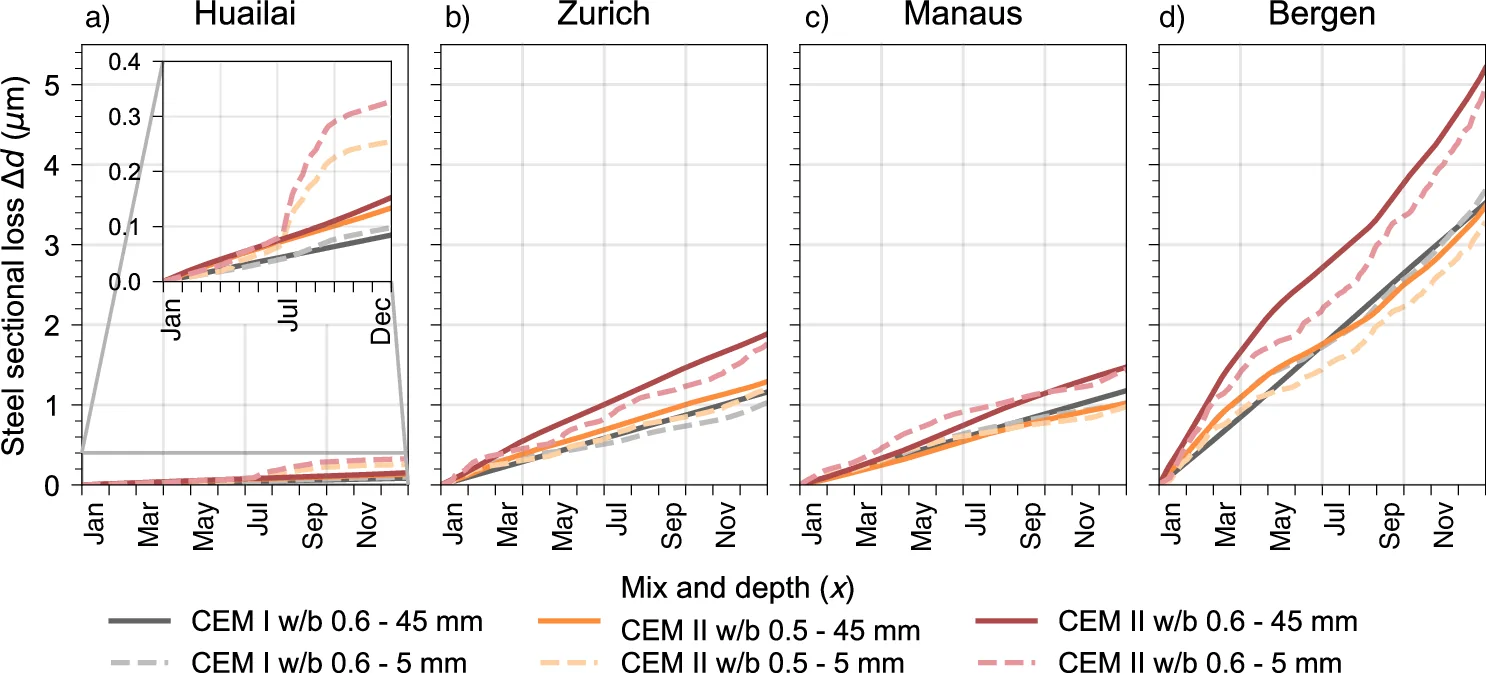
Cumulative steel sectional loss (μm) over a representative year in the three carbonated concretes and for the four geographic locations. Results are plotted for two selected cover depths, 5 and 45 mm, and the four exposure climates Huailai (**a**), Zurich (**b**), Manaus (**c**), and Bergen (**d**). The inset in fig. a shows a magnified view of the comparatively low sectional losses in Huailai. Source data are provided as a Source Data file.

Thus, for a more accurate evaluation of the expected degradation process, consideration of the actual instantaneous corrosion rates, rather than a binary on-off state, is needed. To this end, we use Faraday’s law of electrolysis to convert the hourly instantaneous corrosion rates, expressed as electrochemical current density, to steel sectional loss, thereby allowing the direct comparison of the cumulative steel sectional loss for different materials and locations over a year (Fig. 5). It is interesting to note that the cumulative steel sectional loss is comparable for the traditional, Portland cement based system at high w/b ratio (CEM I w/b 0.6) and for the system with reduced clinker at lower w/b ratio (CEM II B-LL w/b 0.5) in all exposure conditions, suggesting that, at least in these cases, decreasing the w/b ratio of concrete might roughly counterbalance the use of a low-clinker cement in terms of corrosion propagation. In this context, it may be worth noting that replacement of the Portland cement clinker (i.e., transitioning from CEM I to CEM II B-LL in this example) has a significantly greater influence on the overall global warming potential (GWP) of the concrete than the w/b ratio. Applying the Open Concrete tool^81^ to estimate CO_2_-equivalent emissions of concrete, typical CEM I mixes with w/b 0.6 result in approximately 260–320 kg/m^3^, while replacing the Portland cement clinker with limestone, as in the CEM II B-LL concretes, decreases such emissions by around 28–34%, with minor differences between the w/b 0.5 and w/b 0.6 concretes. This reduction in CO_2_ emissions for materials with clinker replacement is consistent with existing literature^82,83^.

While Fig. 5 reflects the ToW-based classification in Fig. 4, especially for the distinct cases Huailai and Bergen, it gives a more quantitative estimate of the damage at the structural level over time, setting the basis for service life considerations discussed in the subsequent section.

### Role of corrosion propagation phase in service life predictions

The service life of a structure can be estimated by combining the period prior to corrosion (initiation phase) and the period during which corrosion occurs and until a limit state is reached (propagation phase) (Fig. 1). To illustrate how a detailed assessment of the corrosion stage affects service life predictions, we performed corresponding calculations. To this aim, the initiation phase was computed as the time for the carbonation front to reach the steel in a specific concrete type, cover depth, and exposure environment, based on a state-of-the-art model, namely the DuraCrete model^57,62,63^. One of the key features of the DuraCrete model is that it takes into account the climatic conditions, particularly moisture, which strongly affects the concrete carbonation rate. The model considers moisture by relying on the ToW concept, which is defined as the fraction of days in a year with precipitation exceeding 2.5 mm. The propagation phase was evaluated as the time at which the cumulative steel sectional loss reached 100 μm^84,85^, computed with the climate-informed moisture transport and corrosion modeling approach described above. This threshold of 100 μm cumulative loss is adopted here as a suitable value for the purpose of illustrating the proposed concept. However, it should be noted that this figure is not universal and depends on several parameters, including corrosion rate, porosity, binder type, concrete cover depth, and concrete mechanical properties (strength, Young’s modulus)^85–87^.

Among the here studied climates, the carbonation phase (initiation phase) was shortest in the semi-arid climate (Huailai), as this led to an intermediate concrete saturation level, well known to provide a near-ideal combination of wet and dry periods, thus simultaneously allowing for CO_2_ gas diffusion, dissolution, and reaction, thereby accelerating the carbonation process^64^. The case of Bergen, on the other hand, exhibited the longest initiation phase, which can be attributed to its comparatively high moisture state, limiting the ingress of CO_2_.

**Fig. 6:**
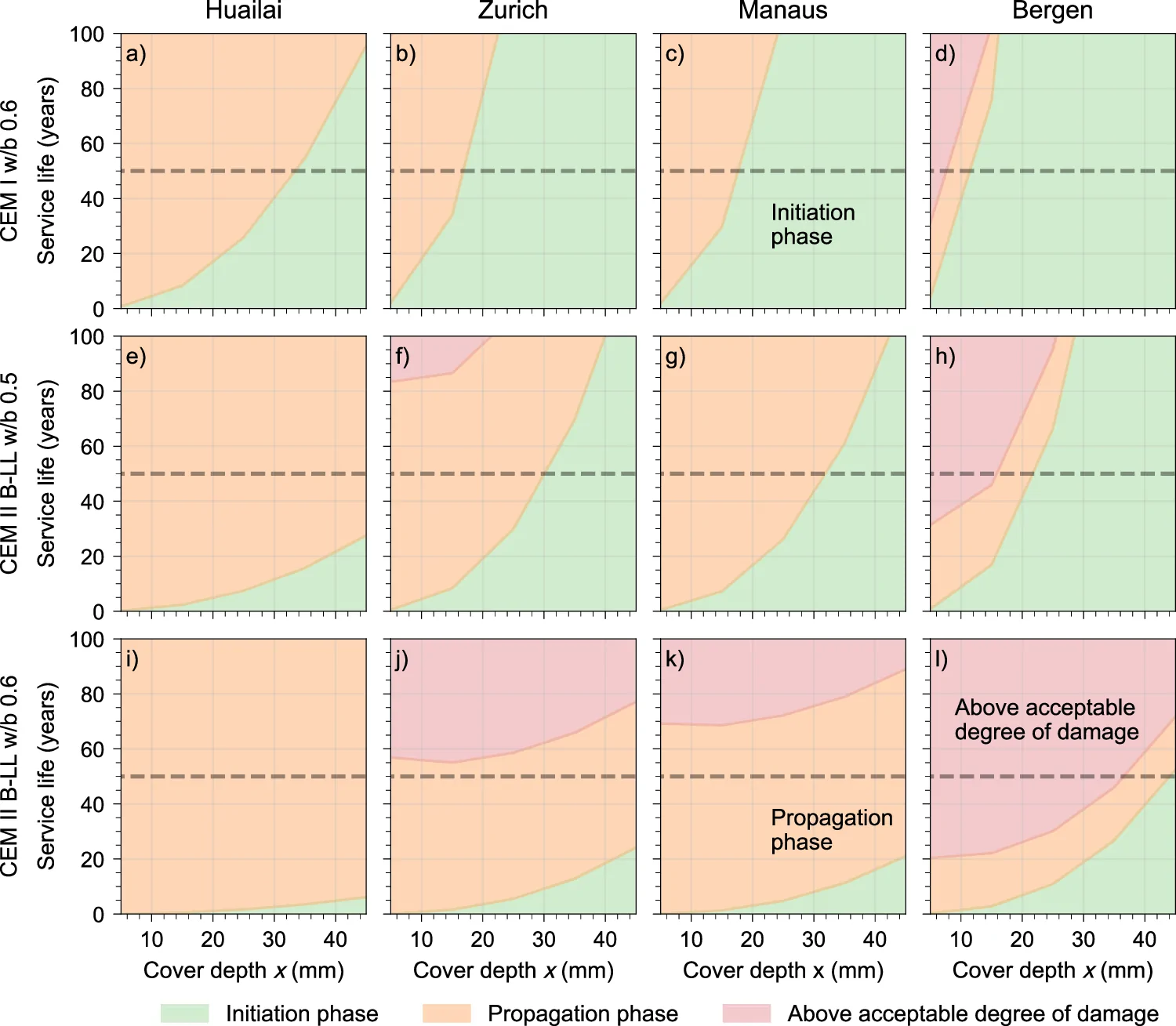
Service life as a combination of the initiation and propagation phase of corrosion in carbonated concrete in XC4 exposure, as a function of the cover depth, cement type, w/b ratio, and geographic location (climate). Specifically, cement CEM I with w/b 0.6 for exposure climates in Huailai (**a**), Zurich (**b**), Manaus (**c**), Bergen (**d**); cement CEM II B-LL with w/b 0.5 for exposure climates in Huailai (**e**), Zurich (**f**), Manaus (**g**), Bergen (**h**); cement CEM II B-LL with w/b 0.6 for exposure climates in Huailai (**i**), Zurich (**j**), Manaus (**k**), Bergen (**l**). Red areas mean that the service life cannot be achieved at the specific cover depth (*x*). According to European standard EN 206, the minimum required cover depths (*x*) for 50 years of service life vary in different countries in the range 30−40 mm. Source data are provided as a Source Data file.

Figure 6 reveals that service lives of 50 years or more cannot always be achieved merely by considering the initiation phase for concretes made from the here considered low-carbon concretes (based on CEM II) with cover depths of 30–40 mm according to European standards (EN 206). However, if the propagation phase is considered as well, this situation changes, and a 50-year service life becomes achievable for all systems with 30 mm cover depth or less, with the exception of CEM II B-LL w/b 0.6 for the case of Bergen, where a cover depth of ∼40 mm is needed.

These results thus illustrate that for low-carbon binders and concretes with diminished resistance against carbonation compared to traditional Portland cement, consideration of the corrosion propagation phase is essential, as it represents a significant part of the service life of a structure. Despite the short initiation phase, the propagation phase can account for the majority of the service life in the semi-arid Huailai, and above 50 years of the service life in wetter climates, such as in Zurich and Manaus. For even higher average RH and precipitation volumes, such as in Bergen, corrosion rates are high and short propagation phases are observed compared to the other locations. However, the time for carbonation to reach the reinforcement also increases at such high moisture levels^64^, and might still surpass the required 50–100 year service life of a structure for most materials. Critical conditions arise when the concrete mix leads to both fast carbonation, even at high moisture levels, and high corrosion rates, as is the case for CEM II B-LL w/b 0.6 in Bergen.

The results in Fig. 6 further emphasize the need to consider the differences in exposure climates. While all four climates qualify as exposure class XC4 “cyclic wet and dry” in accordance with European standards, the significant differences in meteorological conditions, especially precipitation patterns, lead to markedly different performances, with the initiation and corrosion propagation phases contributing differently to the service life.

In summary, this work showcases an approach in which the concrete cover depth is not merely considered a barrier against carbonation but also a barrier against moisture ingress, thus controlling the corrosion propagation phase. This approach is conceptually a significant departure from the state-of-the-art, which can provide a fairer evaluation of the durability of low-carbon reinforced concretes. Ultimately, this approach can support the reduction of CO_2_ emissions in the cement and concrete industry while ensuring the durability and safety of structures, namely by providing a route for future durability design and performance testing.

### Perspective on future durability design and performance testing

Current approaches to ensure the long-term durability of reinforced concrete in carbonation exposure conditions consider and tailor the concrete cover as a barrier against carbonation. The complexity of these state-of-the-art approaches varies depending on whether they are prescriptive or performance-based (Supplementary Note 1). However, in either case, the effectiveness of this barrier is determined by a combination of concrete properties, primarily its resistance against carbonation (a function of cement type and mix proportions) and the cover depth. Future approaches, on the other hand, could expand this concept to also embrace material selection and design by focusing on the concrete’s function as a barrier against moisture ingress, especially in its carbonated state. This philosophy could be supported by laboratory testing that evaluates the carbonated concrete’s resistance to moisture uptake and its moisture retention properties. Such laboratory tests already exist, e.g. water sorption tests, and while they are currently not used in the context of ensuring the corrosion resistance of reinforced concrete under carbonation exposure, they may readily be integrated in future engineering approaches as performance assessment tests, e.g., combined with accelerated carbonation testing.

However, while such standardized laboratory tests provide controlled material characterization, interpreting these results requires a theoretical framework that connects the laboratory conditions to real-world performance. The approach presented here establishes such a framework and addresses another limitation of state-of-the-art design approaches: the oversimplified description of the exposure conditions. Current durability design standards categorize exposure conditions using qualitative exposure classes, yet our results show that concrete can perform very differently within the same class (Fig. 6). This underlines the importance of quantitative approaches that integrate both local climate conditions and material properties, as different materials can exhibit markedly different performance even when falling under the same exposure class.

However, our calculations, as presented in this work, are computationally expensive and require high-resolution, gap-free meteorological data with respect to precipitation and relative humidity, which may not always be available. It should be noted that the approach proposed here is not intended for direct practical application; rather, this fundamentally based concept is meant to serve as a guiding framework for future research and engineering development. Thus, this manuscript encourages future developments to substitute the complex numerical model calculations—considering the full, time-series meteorological input data and related response at the material level—with simplified, yet sufficiently reliable approaches. Such a simplified approach should relate annual or seasonal corrosion rates to proxies, that is, characteristic parameters that describe key features of precipitation and humidity patterns, along with material properties.

A preliminary exploration of such relationships, even with the limited data available in this study (three concrete mixes, four geographic locations), reveals that this is a challenge that requires further consideration (Supplementary Note 9). Simple and readily available climatic measures, such as annual average RH or annual total precipitation, are insufficient for accurately forecasting the corrosion rate. More complex parameters that reflect precipitation patterns, such as the precipitation concentration index (PCI), a common parameter in climate research, fail to adequately capture the full picture alone. This is because moisture transport processes in concrete and the resulting corrosion kinetics of the embedded steel are highly non-linear and are inherently complex due to their temporal dependencies (i.e., each event is influenced by the previous history). Thus, future studies should focus on developing multi-parameter approaches, including the derivation of relevant, new proxies and indices from meteorological data, tailored to the here described application, and establishing relationships with the corrosion rates of steel in carbonated concrete.

Furthermore, to develop a framework that can be applied in practice, future scientific research is necessary to refine the proposed model. This includes addressing the following key areas. For the purpose of illustration, we here assumed a constant threshold for the steel sectional loss of 100 μm to describe the end of the service life, based on earlier work, where this number was considered to correlate with the limit state concrete cracking^84,85^. However, corrosion-induced cracking of the concrete is more complex, and a larger cover depth might have other advantages, beyond delaying carbonation and moisture ingress, such as decreased concrete cover cracking or spalling^20^. This could be due to concrete withstanding higher stresses caused by the corrosion products formed, associated with a higher total steel sectional loss and corrosion rate^84,85,88^. Concrete with increased porosity, such as low-carbon concrete compared to Portland cement systems, has also been suggested to accommodate larger volumes of corrosion products before cracking starts, which might partially compensate for the higher corrosion rates of steel in such concretes^85^. In-depth analyses of the corrosion damage threshold as a function of cover depth could shed light on this deterioration mechanism. Moreover, future work could expand our model framework to consider temperature effects on moisture transport and corrosion. However, the priority should be on addressing the former aspect, both because of the expected relevance (compare Supplementary Note 10) and because the state of the art in this regard is particularly limited.

Additionally, the currently available literature data for the instantaneous corrosion rate of steel in carbonated concrete as a function of the concrete’s moisture state shows limitations in several aspects. First, while literature commonly reports instantaneous corrosion rates as a function of exposure RH (upon equilibration)^29,30^, it rarely provides the necessary data on water sorption isotherms or pore size distribution of the cementitious matrix, essential to reliably convert RH to degree of saturation. Second, although corrosion rates might change in the long-term due to a number of reasons, such as the accumulation of corrosion products at the steel-concrete interface^89^ and alterations in pore structure and electrolyte chemistry, there is limited data available in this regard. Moreover, corrosion rate data for modern low-carbon concretes is scarce, particularly for concrete with high clinker replacements^29,30^, calcined clay systems^90^, or even Portland cement-free concretes like MgO/hydromagnesite systems^91^, where corrosion rates may be higher than in the here considered literature. Finally, most literature corrosion rate data are based on electrochemical measurements, and validation using complementary techniques is limited. Thus, the relationship adopted in this work (Eq. (7), Supplementary Note 8) may be refined as additional data become available. It should also be noted that the corrosion rates used herein are derived from average values reported in the literature and may therefore underestimate peak values, and hence, the actual numbers related to the presented case studies should be applied to practical conditions with caution. In light of the limitations outlined above, we consider expanding the currently available dataset a research priority. Future efforts should focus on extending the data basis on corrosion rates to include (i) a broad range of modern and relevant cementitious binders, (ii) field data under actual exposure conditions to complement the currently predominant laboratory-based studies, (iii) concrete systems rather than mortar specimens to further validate performance, (iv) both short- and long-term behavior, (v) measurement techniques beyond electrochemical methods to enable broader validation of predominantly electrochemical data, and (vi) data expressing corrosion rates as a function of the degree of saturation within the concrete, ideally accounting for the microstructure at the steel-concrete interface, rather than solely in terms of equilibrium exposure RH.

In conclusion, the here presented conceptual approach and model framework allow overcoming different shortcomings of the state-of-the-art in evaluating the durability of reinforced concrete in carbonation exposure conditions. Current approaches oversimplify environmental exposure by classifying conditions as cyclic wetting-drying, which ignores the significant differences between various geographic locations and the related climates. Furthermore, state-of-the-art approaches primarily focus on the carbonation process and overlook the importance of the corrosion propagation phase, a crucial factor in determining the service life of concrete, particularly in eco-friendly systems. The proposed conceptual approach is flexible, allowing for a quantitative consideration of diverse geographic and climatic conditions instead of relying on simplified, qualitative XC categories. This enables a more accurate and equitable assessment of low-clinker concrete, which tends to be disadvantaged by current approaches due to their focus on the initiation phase. Moreover, the proposed durability framework may contribute to postponing repair interventions in existing structures, thereby directly reducing the consumption of critical raw materials and energy. Additional environmental benefits may arise from the extended service life of concrete structures, which can continue to act as carbon sinks^92^ for longer periods compared to scenarios involving earlier demolition of ageing structures.

Finally, our approach offers opportunities to analyze the effects of climate change on the future performance of reinforced concrete. For instance, the presented framework may be used to account for anticipated shifts in precipitation patterns and moisture in the atmosphere over the coming decades, which are expected to vary significantly on a local scale^93^. Our results have shown that the distribution of the rainfall over time plays a major role. Even if the total amount of rainfall is comparable (Bergen and Manaus), corrosion propagation can be very different. Here, this is because Manaus has a relatively constant humid climate, while Bergen exhibits stronger fluctuations with pronounced extended wetting phases. It is exactly these wetting phases that temporarily boost the corrosion rate and lead to an overall more rapid corrosion propagation. Note that moisture effects are much more dominant than temperature effects in controlling the corrosion rate in porous media (Supplementary Note 10). Thus, climate-change-induced shifts in precipitation patterns may pose a problem and may threaten the durability of eco-friendly, reinforced concrete structures much more than a temperature rise by 2–3 °C.

## Methods

### Meteorological evaluation

We selected four locations from a range of different climates, considering gap-free availability of detailed, that is, with hourly resolution or better, and over at least a full year, data about precipitation and RH. We chose Zurich, Switzerland; Manaus, Brazil; Huailai, China; and Bergen, Norway, with publicly available data^75–77^. In Zurich, measurements were available at an interval of 10 min; for the other locations, the interval was 1 h. From this time series data, the data points were categorized into dry and wet intervals. Adjoining wet intervals were considered as a single longer wet phase. Using this classification, the RH data were used to compute the average RH during dry and wet phases. Further analyses included computing percentiles for the duration of wet or dry phases. Finally, the data was used to feed into the boundary conditions of a numerical simulation of moisture transport in concrete. Note, we focus on precipitation-driven moisture ingress into concrete as the primary climate-related factor controlling corrosion of steel in concrete. Temperature effects are excluded from this analysis because their role in corrosion is considered negligible compared to that of moisture (Supplementary Note 10). More details on the meteorological data are given in Supplementary Note 2.

### Materials considered

To exemplify the proposed concept with materials of markedly different properties, we used a combination of two variables: binder type, namely traditional Portland cement (CEM I) and a Portland limestone cement with a lower carbon footprint (CEM II B-LL), and two distinct water-to-binder ratios (w/b 0.5 and 0.6). These materials were selected considering CEM I as the reference and CEM-II/LL due to its widespread use in many countries, including Switzerland^94^, its decreased clinker content and its rapid carbonation compared to CEM I^95^, thus providing a suitable system representative for low-clinker cements.

Experiments were carried out to characterize the materials and particularly to determine moisture transport and retention properties, including porosity, electrical resistivity, moisture sorption isotherms, and water ingress by capillary water absorption. Experimental details and measured results are provided in Supplementary Notes 3–6; the “Methods” section details how the material characterization data were implemented in the numerical simulations.

### Simulations of moisture transport in carbonated concrete

Moisture in concrete exists in different phases, including liquid water and water vapor. When simulating moisture transport in a porous material, these two phases need to be simultaneously considered^70–73^. Hence, a two-phase continuum moisture transport model was employed here. Using the established approach of homogenized simulations for mass transport in porous media^96^, each finite element in the domain was viewed as a homogeneous porous medium consisting of solid phases and pores, whereas the porosity represents the available space for moisture transport. Unsaturated moisture transport in a rigid porous material can be formulated by the Richards’ equation^70^ which is written as Eq. (1):

1 $$\frac{\partial {S}_{l}}{\partial t}=\nabla \left[{D}_{a}\left({S}_{l}\right)\nabla {S}_{l}\right]$$

where ${S}_{l}$ is the degree of liquid water saturation, ${D}_{a}\left({S}_{l}\right)$ is diffusivity, which is a function of ${S}_{l}$. ${D}_{a}\left({S}_{l}\right)$ has contributions from liquid water and water vapor, as shown in Eq. (2):

2 $${D}_{a}\left({S}_{l}\right)={D}_{l}\left({S}_{l}\right)+{D}_{v}\left({S}_{l}\right)$$

The model assumes that equilibrium between liquid water and water vapor is always maintained, which is a reasonable assumption for a porous material with low permeability, such as concrete^97^, where mass exchange between liquid water and water vapor is considerably faster than transport. Hence, sorption isotherms were used to describe the equilibrium between ${S}_{l}$ and RH in the environment. The contributions of liquid water and water vapor to diffusivity ${D}_{a}\left({S}_{l}\right)$ are given as described in Eqs. (3, 4), respectively^70^

3 $${D}_{l}\left({S}_{l}\right)=-{k}_{{rl}}\frac{{K}_{l}}{\phi {\eta }_{l}}\frac{{{{\rm{d}}}}{P}_{c}}{{{{\rm{d}}}}{S}_{l}}$$

4 $${D}_{v}\left({S}_{l}\right)=-{\left(\frac{{M}_{v}}{{\rho }_{l}{RT}}\right)}^{2}{D}_{v0}{\phi }^{{x}_{D}}{\left(1-{S}_{l}\right)}^{{x}_{D}+2}\frac{{P}_{{vs}}{{{\rm{RH}}}}}{\phi }\frac{{{{\rm{d}}}}{P}_{c}}{{{{\rm{d}}}}{S}_{l}}$$

where ${\eta }_{l}$ is the dynamic viscosity of liquid water, ${k}_{{rl}}$ is relative permeability, ${K}_{l}$ is the intrinsic permeability, ${M}_{v}$ is the molar mass of water molecule, ${\rho }_{l}$ is liquid water density, ${x}_{D}$ is a resistant parameter to consider the microstructural effect on vapor diffusion (taking as 2.74 for cement-based materials based on the fitting of experimental data of CO_2_ and oxygen diffusion^98^), and ${P}_{{vs}}$ is the saturated vapor pressure in air. The term $\frac{{{{\rm{d}}}}{P}_{c}}{{{{\rm{d}}}}{S}_{l}}$ is the moisture retention capacity of concrete derived from the water vapor sorption isotherm by using Kelvin equation and the van Genuchten Eq. (5)^99^,

5 $${S}_{l}={\left[{\left(\frac{{P}_{c}}{\alpha }\right)}^{\frac{1}{1-m}}+1\right]}^{-m}$$

where α and m are two material related parameters, which are generally determined by fitting the measured water vapor sorption isotherms for a given material. For the studied materials (CEM I w/b 0.6, CEM II B-LL w/b 0.5, and CEM II B-LL w/b 0.6), sorption isotherms were experimentally obtained by equilibrating mortar samples at a range of RH and determining their corresponding saturation degree at each RH level (more details can be found in Supplementary Note 5).

Relative permeability ${k}_{{rl}}$ is expressed as a function of ${S}_{l}$ by using the van Genuchten - Mualem model^99^ in Eq. (6),

6 $${k}_{{rl}}={{S}_{l}}^{0.5}{\left[1-{\left(1-{{S}_{l}}^{\frac{1}{m}}\right)}^{m}\right]}^{2}$$

where m is taken from van Genuchten equation (see).

Another key input for numerical simulations is water permeability, ${K}_{l}$, which was inversely determined in this study by fitting the simulated mass change, measured by means of water absorption testing^100,101^. The complete results on this evaluation are presented in Supplementary Note 4.

To perform moisture transport simulations, the initial and boundary conditions must be explicitly defined. The simulations started with an initially uniform moisture distribution throughout the entire simulation domain. The collected meteorological data with hourly resolution was used to derive the boundary condition. While sorption hysteresis (i.e., the gap between desorption and adsorption isotherms) is generally expected to influence simulation accuracy^101^, measured water vapor sorption isotherms on carbonated low-clinker mortars show negligible hysteresis (Supplementary Note 5). Therefore, sorption hysteresis was neglected (measured adsorption curves were used for the simulations). Note that the moisture transport was modeled at the bulk material level without considering the effect of the different microstructure at the SCI and its specific sorption isotherms, which are challenging to experimentally measure. Consistently, the corrosion rates of steel were related to the bulk concrete saturation, as described in the subsequent section. This assumption is considered well justified, given the absence of experimental data linking corrosion rates to interfacial porosity; existing correlations are exclusively based on bulk material properties. More details on the moisture transport simulations are given in Supplementary Note 6.

### Corrosion rates of steel in cementitious materials

The rate of steel corrosion in carbonated concrete increases with increasing concrete moisture state^20,30^. Based on available literature data^21,31,33^, we derived Eq. (7) describing the instantaneous corrosion rate as a function of the concrete’s degree of saturation (Supplementary Note 8):

7 $${{{{\rm{i}}}}}_{{{{\rm{corr}}}}}={{{{\rm{k}}}}}_{{icorr} \, {cem}}{{{\rm{\cdot }}}}0.0025\,\exp (6{{{\rm{\cdot }}}}{S}_{l})$$

Here, ${{{{\rm{k}}}}}_{{icorr\; cem}}$ = 1 for CEM I w/b 0.6. Although reliable data on corrosion rates of carbon steel in carbonated low-clinker cementitious materials is limited in the literature, available data suggests that i_corr_ tends to be higher for those materials than for CEM I. Corrosion rates were thus increased for CEM II B-LL w/b 0.5 and w/b 0.6 based on literature factors^21,33,102^ of ${{{{\rm{k}}}}}_{{icorr\; cem}}$ 1.3 and 2.2, respectively (see Supplementary Note 8).

### Service life calculation

The service life was calculated by combining the initiation and propagation phases of corrosion. The initiation phase was considered as the time for the carbonation front to reach the cover depth, *x*, calculated based on the DuraCrete model^57,62,63^, as detailed in Eqs. 8–11. The inverse effective natural carbonation resistance of concrete R_NAC_^−1^ for CEM I w/b 0.6 was assumed as 4.5 × 10^−4^ (mm^2^/s)/(kgCO_2_/m^3^), based on Table 7.2 of the DuraCrete statistics document^63^. Since R_NAC_^−1^ was not available for CEM II B-LL, the carbonation depth of these mixes was scaled using a custom carbonation depth factor ${{{{\rm{k}}}}}_{{cem}}$. This factor was derived from the ratio of carbonation rates reported for mortars produced with equivalent cements^95^: 1.6 mm/year^0.5^ for CEM I w/b 0.6, 2.8 mm/year^0.5^ for CEM II B-LL w/b 0.5, and 5.5 mm/year^0.5^ for CEM II B-LL w/b 0.6. In addition, the average RH considered was computed from the boundary conditions used for the moisture transport simulations, where the RH was considered as 99.9% during rain events.

8 $${{{{\rm{x}}}}}_{c}({{{\rm{t}}}})={{{{\rm{k}}}}}_{{cem}}\cdot \sqrt{2\cdot {{{{\rm{k}}}}}_{e}\cdot {{{{\rm{k}}}}}_{c}\cdot {{{{\rm{R}}}}}_{{{{\rm{NAC}}}}}^{-1}\cdot {{{{\rm{C}}}}}_{a}}\cdot \sqrt{{t}}\cdot W(t)$$

9 $${{{{\rm{k}}}}}_{e}=1.138{\left[1-{\left(\frac{{{{\rm{RH}}}}}{100}\right)}^{5}\right]}^{2.5}$$

10 $${{{{\rm{k}}}}}_{c}={\left(\frac{{{{{\rm{t}}}}}_{{{{\rm{c}}}}}}{7}\right)}^{-0.567}$$

11 $$W\left(t\right)={\left(\frac{{{{{\rm{t}}}}}_{0}}{{{{\rm{t}}}}}\right)}^{\frac{{({p}_{{dr}}\cdot {{{\rm{ToW}}}})}^{{b}_{w}}}{2}}$$

${{{{\rm{x}}}}}_{c}({{{\rm{t}}}})$: carbonation depth at time t, in mm

${{{{\rm{k}}}}}_{{cem}}$: custom carbonation depth factor for different mixes, equal to 1, 1.75 and 3.43 for CEM I w/b 0.6, CEM II B-LL w/b 0.5 and CEM II B-LL w/b 0.6, respectively.

$${{{{\rm{k}}}}}_{e}$$: environmental factor

$${{{{\rm{k}}}}}_{c}$$: curing factor

${{{{\rm{R}}}}}_{{{{\rm{NAC}}}}}^{-1}$: inverse effective natural carbonation resistance of concrete, 4.5E-4 (mm^2^/s)/(kgCO_2_/m^3^)

${{{{\rm{C}}}}}_{a}$: environmental CO_2_ concentration, 0.005 kgCO_2_/m^3^

t: exposure time (s)

W(t): wetting factor

${{{\rm{RH}}}}$: Relative Humidity (%), equal to 54.8%, 76.7%, 74.6% and 80.9% for Huailai, Zurich, Manaus and Bergen, respectively.

${{{{\rm{t}}}}}_{{{{\rm{c}}}}}$: curing time, 7 days

${{{{\rm{t}}}}}_{0}$: reference time, 28 days = 2419200 s

${p}_{{dr}}$: probability of driving rain, 0.1

${{{\rm{ToW}}}}$: time of wetness, fraction of days in a year with precipitation ≥ 2.5 mm. ${{{\rm{ToW}}}}$ is 6%, 27%, 28% and 45% for Huailai, Zurich, Manaus, and Bergen, respectively, as calculated from meteorological data (see Supplementary Note 2).

$${b}_{w}$$: regression exponent, 0.446

The propagation phase was calculated by extrapolating the considered year of instantaneous corrosion rates as a function of time-resolved exposure conditions over longer time spans, namely by replicating this representative year in sequence. As a limit state, a cumulative steel attack depth of 100 μm was assumed as the maximum tolerable damage before concrete cracking, based on literature^84,85,87^. We highlight that this value is not absolute and depends on several factors, including the corrosion rate, concrete cover, porosity and mechanical strength of concrete. Instantaneous corrosion rates were converted to attack depth by means of Faraday’s first law of electrolysis.

## Supplementary information

Supplementary Information

Transparent Peer Review file

## Source data

Source Data

## Data Availability

Source data are provided with this paper.
