# Supplementary material for: Rethinking concrete durability for low-carbon concretes through climate-informed corrosion modelling
Source: Nat Commun. 2026 Aug 21;17:9999. doi: 10.1038/s41467-026-76916-3 (PMC13590661; doi:10.1038/s41467-026-76916-3)
Supplement: Supplementary file 1 [file 41467_2026_76916_MOESM1_ESM.pdf]

# Supplementary Information

## Title

## Rethinking Concrete Durability for Low-Carbon Concretes through Climate-Informed Corrosion Modelling

## Authors

Cristhiana Albert[1*], Thilo Schmid[1*], Zhidong Zhang[1], Ueli Angst[1**]

## Affiliations

[1]Institute for Building Materials, ETH Zurich, Zurich, Switzerland

* These authors contributed equally

** Corresponding author: uangst@ethz.ch

## Supplementary Note 1 – Standards

National standards on durability requirements for reinforced concrete structures in selected countries in Europe, America, and Asia were reviewed and summarized in Supplementary Table 1 . The analysis focused on methodologies used to address carbonation-induced corrosion, with respect to the initiation and propagation phases of corrosion. Emphasis was given to the considerations for low-clinker cements.

*Supplementary Table 1 – Summary of standards on durability design of reinforced concrete for carbonation-induced corrosion.*

| Country | Standard | Approach | Propagation period | Method | Considerations on low-clinker cements |
|---|---|---|---|---|---|
| **Switzerland** | EN206-1 (NA)[1]; SIA 262, Annex I[2] | Prescriptive + Performance-based | Neglected | Tables with empirical values of cover depth, w/c ratio and minimum cement content are provided according to exposure class. Equations with empirical parameters are proposed for the k-value correction of w/c ratio and minimum cement content of blended cements.<br>The carbonation rate is calculated based on accelerated carbonation experiments (SIA 262, Annex I), and an empirical equation relating carbonation depth with the square root of time. | According to exposure class, same cover depth, w/c ratio and minimum cement content for CEM I and other cements, except CEM II B-LL with 20 kg/m3 higher minimum cement content (EN 206 NA).<br>Partial replacement of CEM I or CEM I/A-LL with fly ash, silica fume and slag is permitted by conservative correction of the cement content and w/c ratios with the k-value. |
| **Germany** | DIN EN206-1 (NA)[3]: 2024; DIN EN 1045-2[4] | Prescriptive + Performance-based | Neglected | Tables with empirical values of cover depth, w/c ratio and minimum cement content are provided according to exposure class.<br>Equations with empirical parameters are proposed for the equivalent performance calculation in terms of w/c ratio and minimum cement content of blended cements. | According to exposure class, same cover depth, w/c ratio and minimum cement content for CEM I and other cements (EN 206 NA).<br>Partial replacement of cements with fly ash, silica fume, and slag is permitted by conservative correction of the cement content and w/c ratios with the k-value. Low-clinker cements CEM II/B-LL, CEM II/B-L, CEM II/A,B-W, CEM III/C, CEM IV and CEM V are not recommended for exposure XC3 and XC4 (DIN EN 1045-2). |
| **United Kingdom** | BS 8500-1, 2:2023[5] BS flex 350, 2:2024[6] | Prescriptive + Performance-based | Neglected | Tables with empirical values of cover depth and strength class are provided according to exposure class. An additional performance-based standard for alternative binder systems has been proposed, evaluating them based on their carbonation rate. | According to exposure class, a combination of cover depth and strength class for CEM I and other cements. |
| **Belgium** | NBN B15-001:2018[7], NBN B15-100: 2018[8] | Prescriptive + Performance-based | Neglected | Tables with empirical values of cover depth, w/c ratio, strength class and minimum cement content are provided according to exposure class, combining carbonation with frost-damage and chloride attack. | Low-clinker and innovative cements can be applied if carbonation properties are comparable to regulated cements. For certain exposure classes which combine carbonation with frost-damage and chloride attack, CEM II and CEM III are not permitted. |

| Country | Standard | Approach | Propagation period | Method | Considerations on low-clinker cements |
| --- | --- | --- | --- | --- | --- |
| | | | | A performance-based standard allows the use of low-clinker and innovative cements comparable to regulated cements after a series of durability tests, including accelerated carbonation. | |
| **Norway** | NS-EN 206:2013+A2: 2021/NA:2022 [9]; NS-EN 12390-10 :2018[10] | Prescriptive + Performance-based | Neglected | Tables with empirical values of cover depth, w/c ratio and minimum cement content are provided according to exposure class, which are related to durability classes. Performance-based criteria are provided. | According to exposure and durability class, same or lower maximum w/c ratio for CEM II/A and CEM II/B compared to CEM I. Other cements may be evaluated by performance-based criteria. |
| **Denmark** | DS 206:2025[11]; NT BUILD 357[12] | Prescriptive + Performance-based | Neglected | Tables with empirical values of maximum w/c ratio, minimum cement content, and maximum SCM content are provided according to exposure class (which are the same for XC2, XC3 and XC4). Performance-based standards (NT BUILD) may be used in certain circumstances. | According to exposure class, same maximum w/c ratio and minimum cement content for CEM I, CEM II/A and CEM II/B. |
| **Spain** | EHE- 8 (2008); Chapter VII, Annex 9[13] | Prescriptive | Considered, proposing empirical equation for corrosion rate | Corrosion initiation and propagation periods can be considered. Carbonation is calculated by an empirical equation relating carbonation depth with the square root of time, proposing empirical carbonation rates for different cement types.<br>Corrosion propagation time is calculated by empirical equation and is proportional to cover depth and indirectly proportional to rebar diameter and corrosion rate in the environment (with suggested values according to the exposure class). | According to exposure class, higher cover depth for cements other than CEM I (Chapter VII). For carbonation depth calculation, CEM I, CEM II/A, CEM II/B-S, CEM II/B-L, CEM II/B-LL, CEM II/B-M, CEM/V have the same cover depth for w/c ratio, while other cements have a higher cover depth for same w/c ratio (Annex 9); CEM II/A,B-Q, CEM II/A,B-W, CEM II/A,B-T, CEM III/C and CEM V/B are not recommended for reinforced concrete (Article 26) |
| **Portugal** | LNEC E 464:2007; NP EN 1992-1-1/NA:2010[14,15] | Prescriptive | Neglected | Tables with empirical values of cover depth, w/c ratio and minimum cement content are provided according to exposure class. | According to exposure class, same cover depth, w/c ratio and minimum cement content for CEM I and CEM II/A. CEM II/B, CEM III/A (min 50 mass.-% clinker) have a higher minimum cement content for same cover depth, w/b ratio. CEM IV, CEM V/A require a lower w/c ratio for same cover depth. |
| **Brazil** | NBR 6118:2023[16]; NBR 12655:2015[17]; NBR 15575-2:2013[18] | Mostly Prescriptive + Performance-based | Only broadly mentioned | Tables with empirical values of cover depth, w/c ratio and minimum cement content are provided according to exposure class.<br>Performance-based standard (NBR 15575-2) allows the use of models for carbonation and corrosion prediction, but does not specify which methods can be used. | According to exposure class, same cover depth, w/c ratio, strength class and minimum cement content for CEM I and other cements. If approved by carbonation/corrosion models, alternative cement types could be employed. |

| Country | Standard | Approach | Propagation period | Method | Considerations on low-clinker cements |
|---|---|---|---|---|---|
| **United States** | ACI 318:2019[19], ACI 222:2019[20], ACI 365.1R:2017[21] | Prescriptive + Performance-based | Only broadly mentioned in ACI 365.1 | Tables with empirical values of cover depth, w/c ratio and minimum cement content are provided according to exposure class. Possible to decrease cover depths if alternative protection methods are used. ACI 365.1 provides general guidance on carbonation and corrosion prediction, considering methods based on "experience, deductions from performance of similar materials, accelerated testing, applications of reliability and stochastic concepts, and mathematical modeling based on the chemistry and physics of degradation processes". | According to exposure class, same cover depth, w/c ratio and strength class for CEM I and other cements (with up to 70% PC replacement). Possible to use alternative materials upon performance-based validation, but does not specify which methods can be used. |
| **Canada** | CSA A23.1-09; CSA S413:14 | Performance-based | Neglected | No definition of carbonation exposure class, but mention of limiting w/c ratio for cover depths < 50 mm to avoid carbonation. No criteria to evaluate the material performance against carbonation is provided. Corrosion inhibition strategies are presented in CSA S413:14. | Blended cements (HVSCM) have a limiting w/c ratio for cover depths <50 mm, whereas no limits are defined for Portland cement. |
| **China** | GB/ T 50476-2019[22], CCES 01-2004[23] | Prescriptive | Neglected | Tables with empirical values of concrete strength class, cover depth, and w/c ratio are provided according to exposure class. | According to exposure class, blended cements with slag and fly ash are allowed with a max replacement of 50%. |
| **India** | IS 456:2000[24] | Prescriptive | Neglected | Tables with empirical values of cover depth, w/c ratio and minimum cement content are provided according to exposure class. | According to exposure class, same cover depth, w/c ratio and minimum cement content for CEM I and other cements, with limiting clinker replacement by 35% fly ash and 60% slag. |
| **Japan** | JSCE 15:2007[25], JSCE 16:2007[26] | Prescriptive + Performance-based | Considered in one of the approaches, allowing a critical width of concrete cracks, based on empirical equations | Approach avoiding carbonation to reach the reinforcement (by matching cover depth, w/c ratio, concrete carbonation rate) or accepting corrosion propagation and cracking below limiting values. Tables with empirical values of cover depth and w/c ratio are provided according to exposure class. Carbonation progress can be calculated by (i) empirical square root of time model, proposing carbonation rates for different cements, (ii) accelerated testing, or (iii) (unspecified) physicochemical models. Before cracking, steel corrosion progress can be determined by (i) methods based on corrosion data from inspections or (ii) methods based on corrosion rates of steel obtained with accelerated testing. Concrete cracking can be predicted by (i) methods | According to exposure class, same cover depth and w/c ratio for CEM I, and CEM II B with fly ash or slag. Carbonation progress can be calculated by empirical equation assuming higher carbonation rates for cements other than CEM I. |

| Country | Standard | Approach | Propagation period | Method | Considerations on low-clinker cements |
|---|---|---|---|---|---|
| | | | | based on amount of corrosion or (ii) mechanical models. An equation for the steel corrosion depth limit (to avoid concrete cracking) is proposed based on assumptions and finite element analysis, but values from experiments can also be adopted. Steel corrosion rates might also be calculated with an empirical equation based on rain events in Japan, cover depth, concrete permeability to water, and carbonation, assuming parameters based on experimental data. Corrosion rates can be assumed constant or vary over time. Extreme carbonation and water ingress cases might lead to higher corrosion rates and require complementary experiments. The limiting factor for corrosion propagation is the critical crack width, as a function of the cover depth, and it is empirically defined. | |

## Supplementary Note 2 – Meteorological data

### *Choosing data from different geographic locations*

From publicly available meteorological data[27–30], the four locations were selected data to represent different climates and meteorological conditions, as shown in Table 1 in the main text. Huailai is the driest climate, having relatively little total precipitation and the fewest precipitation days. Additionally, its average RH is much lower compared to the other locations, which share similar values. While Zurich experiences more precipitation days, the amount of precipitation in Manaus exceeds that of Zurich considerably. Bergen is the wettest climate with a similar amount of annual precipitation as Manaus, but far greater number of precipitation days.

Note that we only considered rain as precipitation relevant for moisture transport and corrosion of steel in concrete. Other forms of precipitation, such as snow, were excluded on the following basis. In Manaus, the temperature always remains above zero. The meteorological data available for Huailai does not distinguish between rain and snow. However, since the rain season in Huailai is in summer, there is practically no precipitation during the colder months when snow could form. For Zurich, the precipitation data is presented separately as snow and rain, and only the rain contribution is considered here. For Bergen, while precipitation is given collectively (not distinguishing snow and rain), additional information shows that snow accounts for only around 2% of the annual precipitation[31]. This is because of the influence of the gulf stream that keeps the air temperature in the coastal region of Bergen close to or above freezing temperature throughout the winter.

### *Time series analysis*

For these locations, we acquired time series data containing the relative humidity, and the precipitation during the interval between the time steps. The measurement interval was 10 minutes for the data from Zurich, Switzerland, and 1 hour for all other locations. An example is shown in Supplementary Figure 1. Note that for better readability, the data has been aggregated into 1 hour intervals, with the relative humidity averaged and the precipitation summed. Histogram of the relative humidity (RH) measurements are shown in Supplementary Figure 2. It also includes the average RH during dry and wet periods.

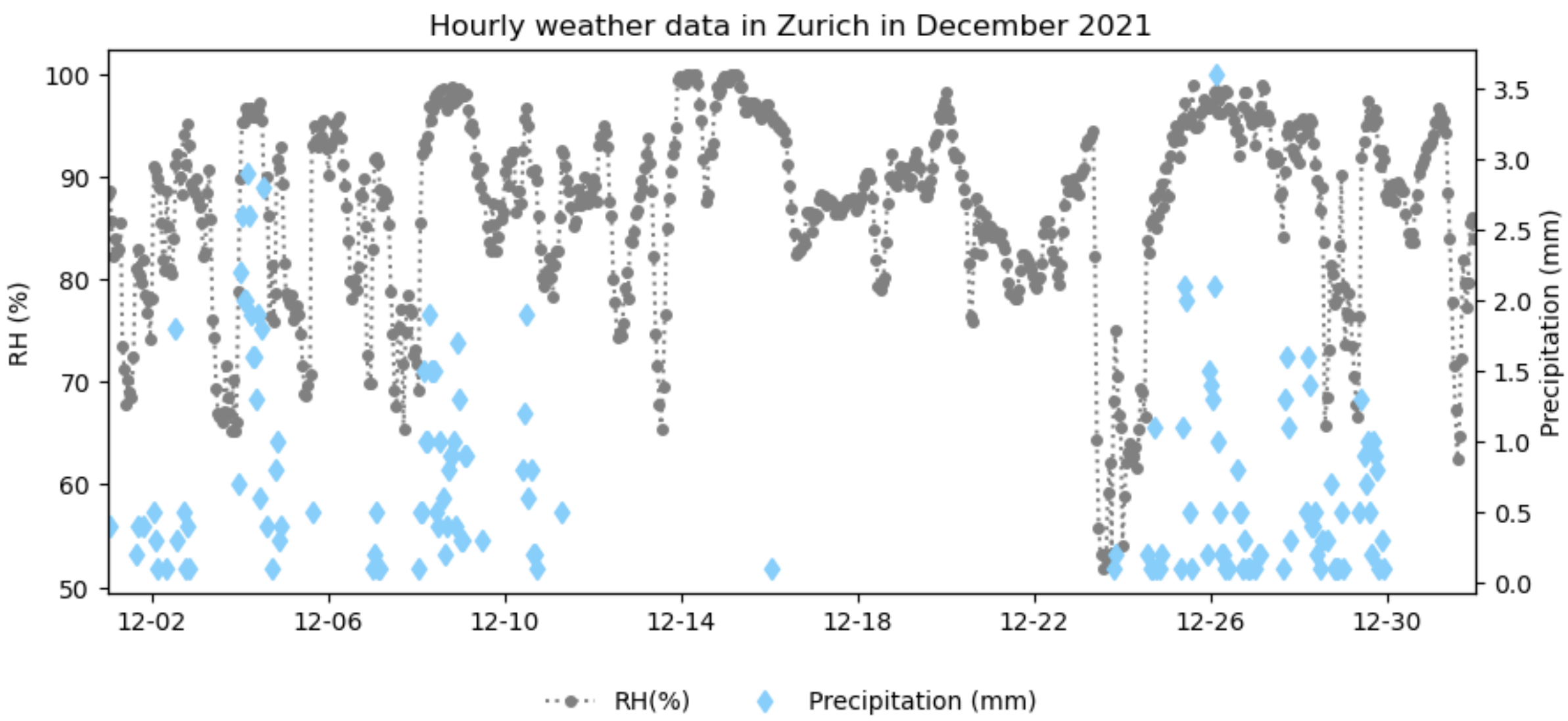


*Supplementary Figure 1 - Example of the Relative Humidtiy (RH) time series data used in this project. It shows the measured data for the month of December 2021 in Zurich, with an hourly resolution. For the precipitation data, the zero measurements are not shown. Source data are provided as a Source Data file.*

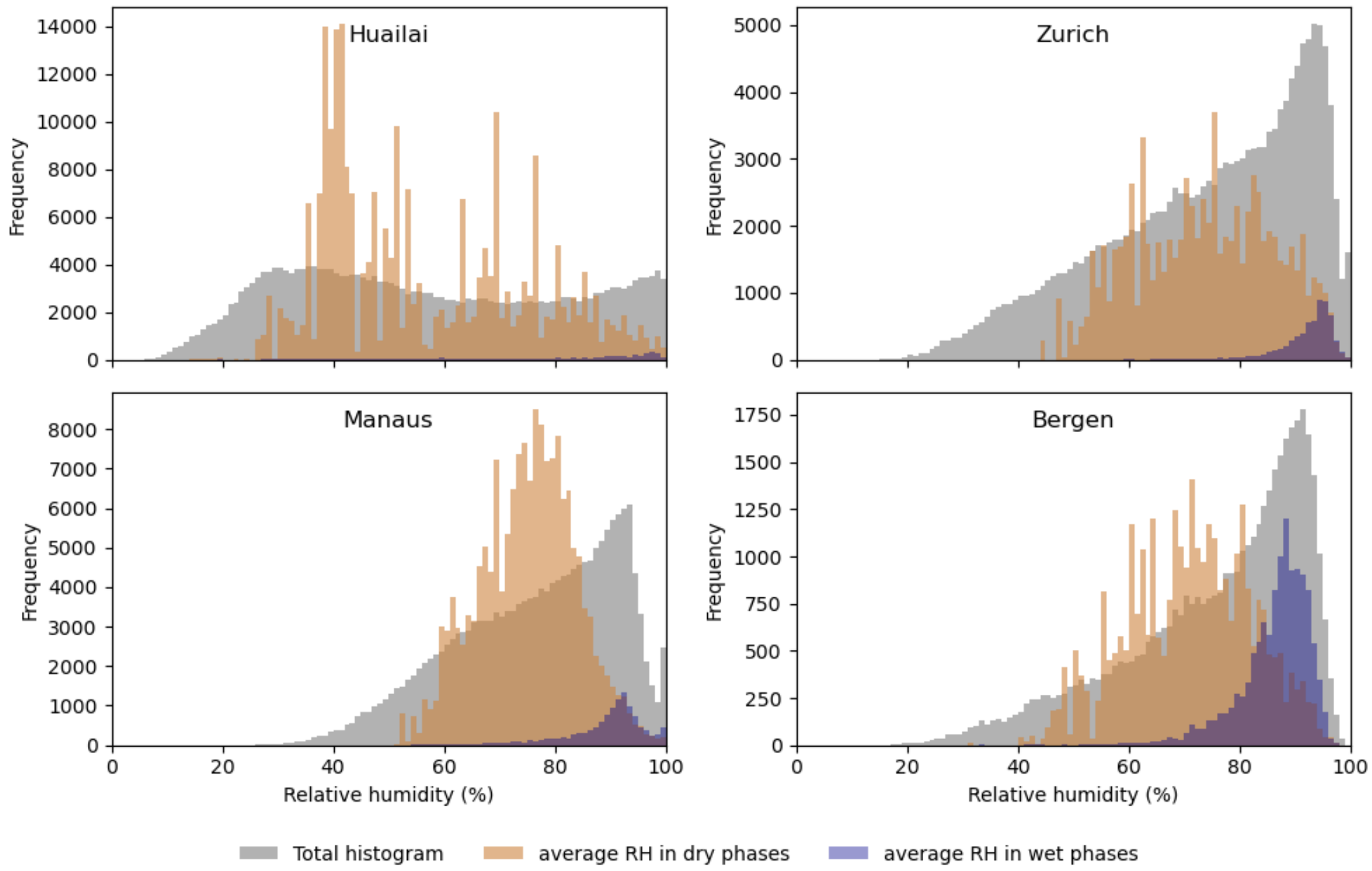


*Supplementary Figure 2 - Relative humidity (RH) across all measurements (grey) and the average RH in dry (orange) and wet (blue) periods. For each dry or wet period, the average value was computed and weighed by the durations of the respective phase. Source data are provided as a Source Data file.*

The recorded precipitation was used to divide the intervals into wet (precipitation >0) and dry (precipitation = 0) phases. Adjacent time intervals with non-zero measured precipitation were grouped into the same wet period. Similarly, successive time intervals without any precipitation were categorized as a dry period. The distribution of lengths of the wetting phases is presented in Supplementary Figure 3. The dashed line indicates the 90$^{th}$ percentile. Therefore, 90 percent of all precipitation events in these four locations are shorter than 2, 1.5, 4, or 10 hours for the four locations considered, respectively. Note that the better time resolution for the data from Zurich resulted in a finer histogram compared to the other locations. This suggests that, while the total rain amount is similar between Manaus and Bergen, Bergen experienced longer continuous rainfall, whereas Manaus saw more short bursts of rain. Conversely, in Zurich the weather seems to be more variable, because both short dry and short wet periods are more likely compared to Bergen and Manaus. For Huailai, which experiences little precipitation, we can observe by the long tail of the dry period histogram that long dry periods are common.

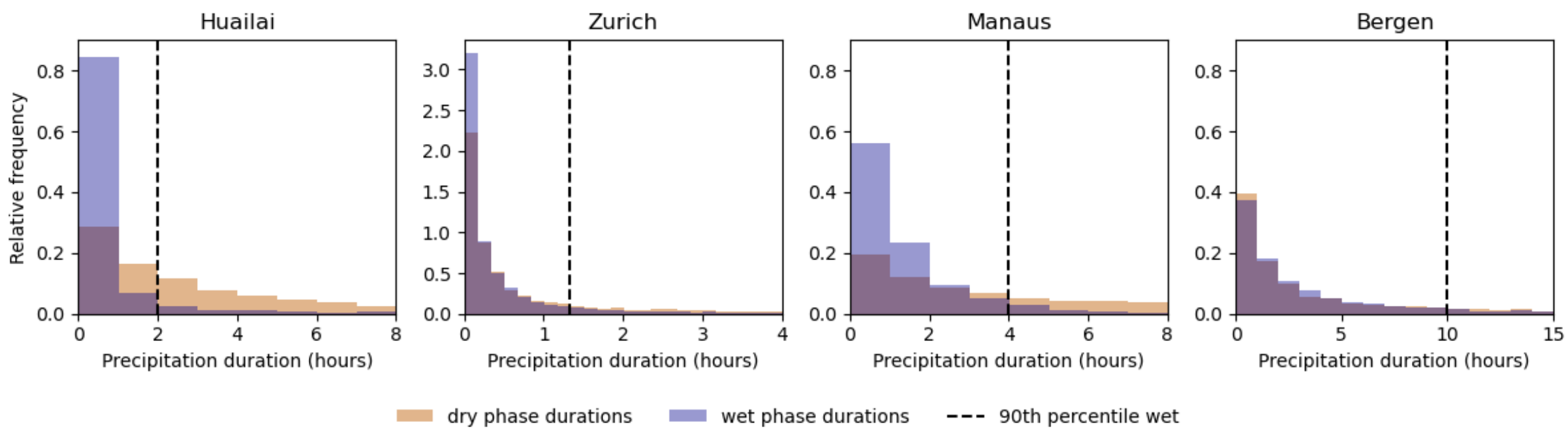


*Supplementary Figure 3 - Duration of precipitation events. The 90th percentile is marked by the dashed line. Note the different x-axis limits for Bergen and Zurich, and the different y-axis limits for Zurich. Source data are provided as a Source Data file.*

### *Variability index*

To compare the variability of the precipitation between those four locations, various statistical quantities were computed. In the literature, studies on precipitation variability are mostly concerned with seasonal or at most daily variations. One example of a commonly used metric to express such variations is the Precipitation Concentration Index (PCI), which is computed the following formula[32]

$$\mathrm{PCI} = \frac{\sum P_i^2}{(\sum P_i)^2} \times 100$$

Here, $P_i$ is the amount of precipitation in period $i$. Usually, this period is months, and the PCI quantifies the variation in precipitation between different months. We have adapted the PCI to concern weekly variations, thus $P_i$ is the accumulated precipitation over a week and the index $i$ runs through the 52 weeks of the year. We call this the weekly PCI. The results for the monthly and weekly PCI are shown in Supplementary Figure 4 a) and b), respectively. Monthly PCI values range from 8.3 to 100, although values above 50 are rarely reached. PCI values below 10 represent evenly distributed rainfall, and values higher than 10 stand for seasonal rainfall[32]. Thus, we see that all locations exhibit at least seasonal variations in the precipitation, with only Huailai having strongly irregular precipitation. The weekly PCI shows an even stronger discrepancy between Huailai and the other locations, indicating that Huailai has weeks with much more intense rainfall compared to other weeks of the year.

For modelling moisture in concrete, we are also interested in hourly or even more finely resolved variations. However, the PCI did not yield useful results for such small periods. A different quantity that showed a clear difference between the four locations was the autocorrelation function, which computes the correlation between the time series measurement data and a shifted version of itself. The most pronounced results were obtained for a shift of 1 hour of the precipitation amounts, shown in Supplementary Figure 4c. In this case, the value is highest among the four locations for Bergen. This means, that the precipitation amount at a given moment is more strongly correlated to the precipitation amount measured one hour earlier, and therefore longer wetting events are more likely to occur in Bergen than in the other three locations. In contrast, the value for Huailai is almost zero, meaning that it is unlikely for precipitation to sustain more than one hour. For Zurich, the autocorrelation is lower than Bergen, but higher than for Manaus. Thus, Zurich is more likely to experience precipitation that lasts for more than one hour than Manaus.

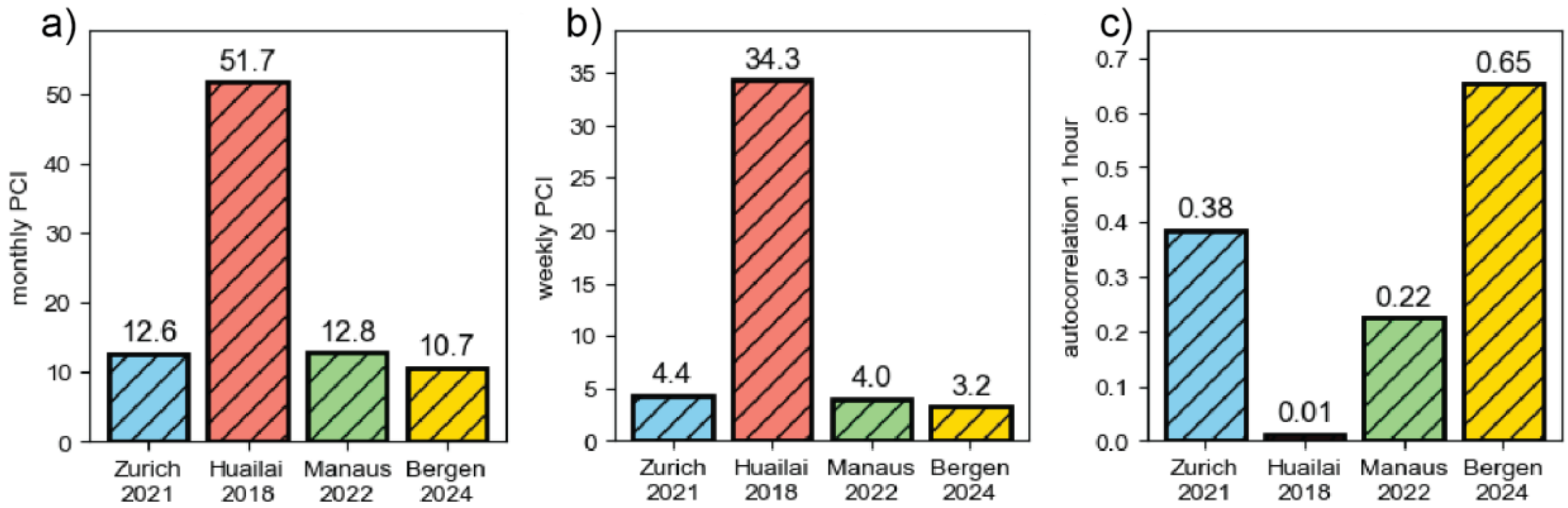


*Supplementary Figure 4 – Variability indices for the four locations and the selected years. a) monthly PCI. b) weekly PCI. c) Autocorrelation function of the precipitation data for a time shift of 1 hour. Source data are provided as a Source Data file.*

### *Temperature variability*

In addition to the precipitation variability of the four locations, also the temperature variability in the meteorological data was analyzed. Supplementary Figure 5 shows the daily and hourly variations of temperature for the four considered locations. Huailai and Zurich show a wider range of daily variations regularly reaching daily temperature differences higher than 10 degrees. In the case of Manaus and Bergen the range is narrower and below 10 degrees Celsius in the majority of cases. Based on these data, and for the reasons detailed in Supplementary Note 10, temperature was not included in the model described in the manuscript, as its influence is considered secondary compared with the dominant role of moisture.

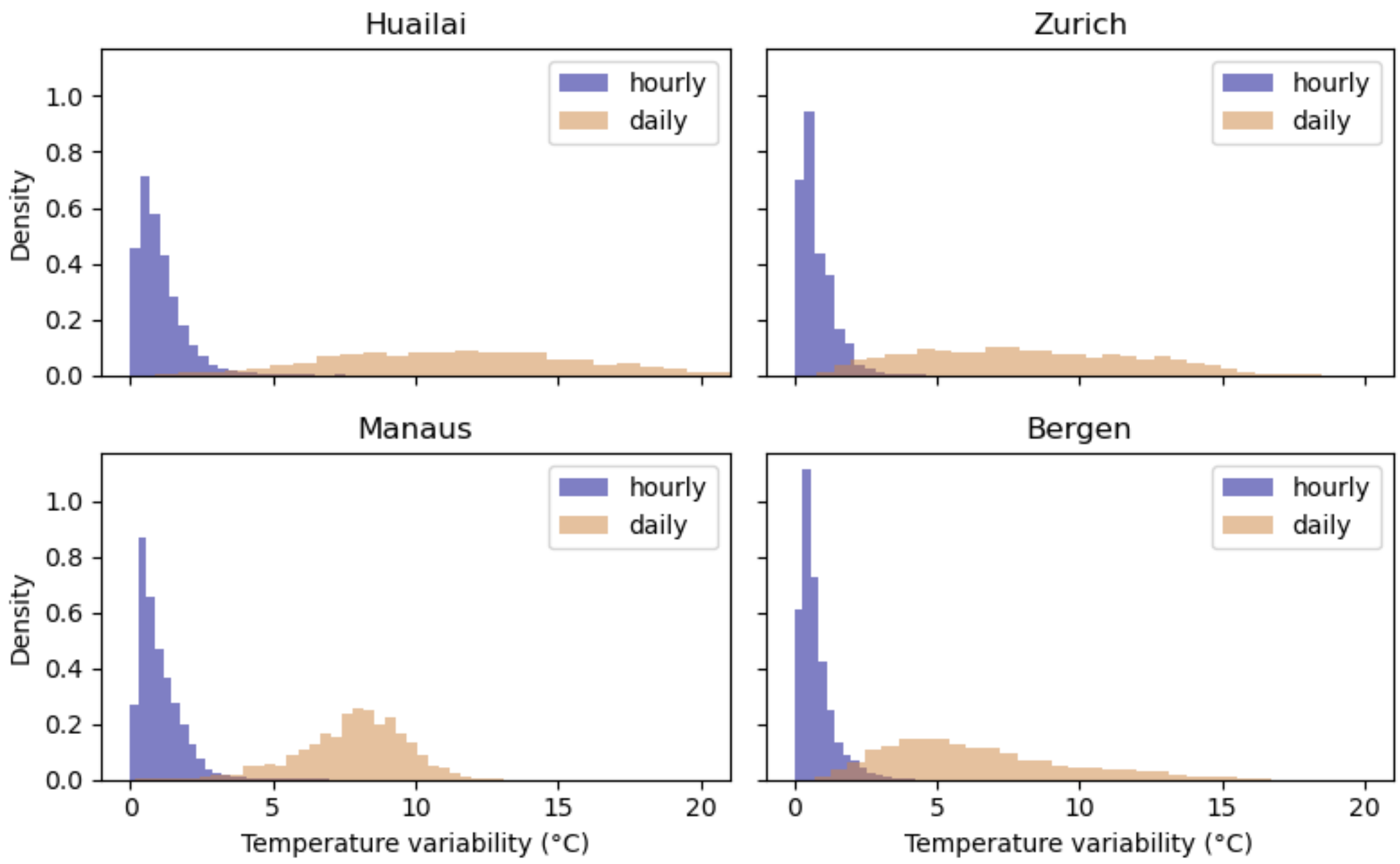


*Supplementary Figure 5 – Daily and hourly variations of temperature. This analysis considers the difference between the maximum and minimum temperature reached per hour or day, respectively. Source data are provided as a Source Data file.*

## Supplementary Note 3 – Materials

The mortar samples were produced with Portland cement (CEM I) and a low-clinker cement with up to 35% limestone (CEM II B-LL), provided by Vigier Cement in Switzerland. The mortars were produced with water/binder (w/b) ratios of 0.5 and 0.6, utilizing sand with a 4 mm maximal grain size, and sand-to-binder ratio of 3:1 in all cases. The mortar specimens were cured in a 95% RH chamber, with curing durations of 28 days for CEM I and 56 days for CEM II B-LL, reflecting the slower microstructure development of the latter. After curing, the samples were carbonated at 4% $CO_2$, 60±3% RH, and a temperature of 20°C. The complete carbonation of the mortar samples was verified with phenolphthalein indicator[33,34].

The porosity and resistivity of the carbonated mixes are provided in Supplementary Table 2. Porosity was determined by the weight method using the water absorption mortar samples. The mass differences between their dry state ($m_{dry,}$ dry at 65°C) and saturated state ($m_{sat}$, vacuum saturated, as described in Supplementary Note 5) divided by the volume of the mortars gives the porosity, as shown by the following equation:

$$\phi = \frac{V_{water}}{V_{mortar}} = \frac{m_{sat} - m_{dry}}{V_{mortar} \cdot \rho_{water}}$$

The impedance of the saturated mortars was measured using an LCR meter at a frequency of 2 kHz, which was connected to two stainless steel rods (2 mm diameter) embedded in the mortars and spaced 20 mm apart. To obtain the resistivity from the impedance, the cell constant was determined from a finite elements simulation modeling the primary current distribution in the specimen geometry using COMSOL Multiphysics[35].

*Supplementary Table 2 – Measured material properties*

| Parameter | Unit | CEM I w/b 0.6 | CEM II B-LL w/b 0.5 | CEM II B-LL w/b 0.6 |
|---|---|---|---|---|
| Porosity $\phi$ | - | 0.156 | 0.191 | 0.214 |
| Resistivity (saturated) | Ωm | 309 | 140 | 80 |

Additional measurements with carbonated mortar mixes produced from comparable cement types (Portland cement and partial replacement by limestone and w/b of 0.6) exhibited pore solutions with pH ~9.5, according to pore water expression measurements. Therefore, the mortar mixes used in this work (CEM I w/b 0.6, CEM II B-LL w/b 0.5, CEM II B-LL w/b 0.6) can reasonably assumed to have pH ~9.5.

## Supplementary Note 4 – Water ingress by capillary absorption

For the capillary absorption tests, specimens with 30x40x50 $mm^3$ were coated with epoxy on the sides not in contact with the water layer, in order to promote the ingress of water through a single surface with known surface area (30x40 $mm^2$). After mass equilibrium at 85% RH, the specimens were positioned in supports over a water bath. The capillary water ingress in the mortars was determined by the mass gain of the specimens over time, as illustrated in Supplementary Figure 6. The water absorption curve is used to calibrate the moisture transport model, as described in Supplementary Note 6.

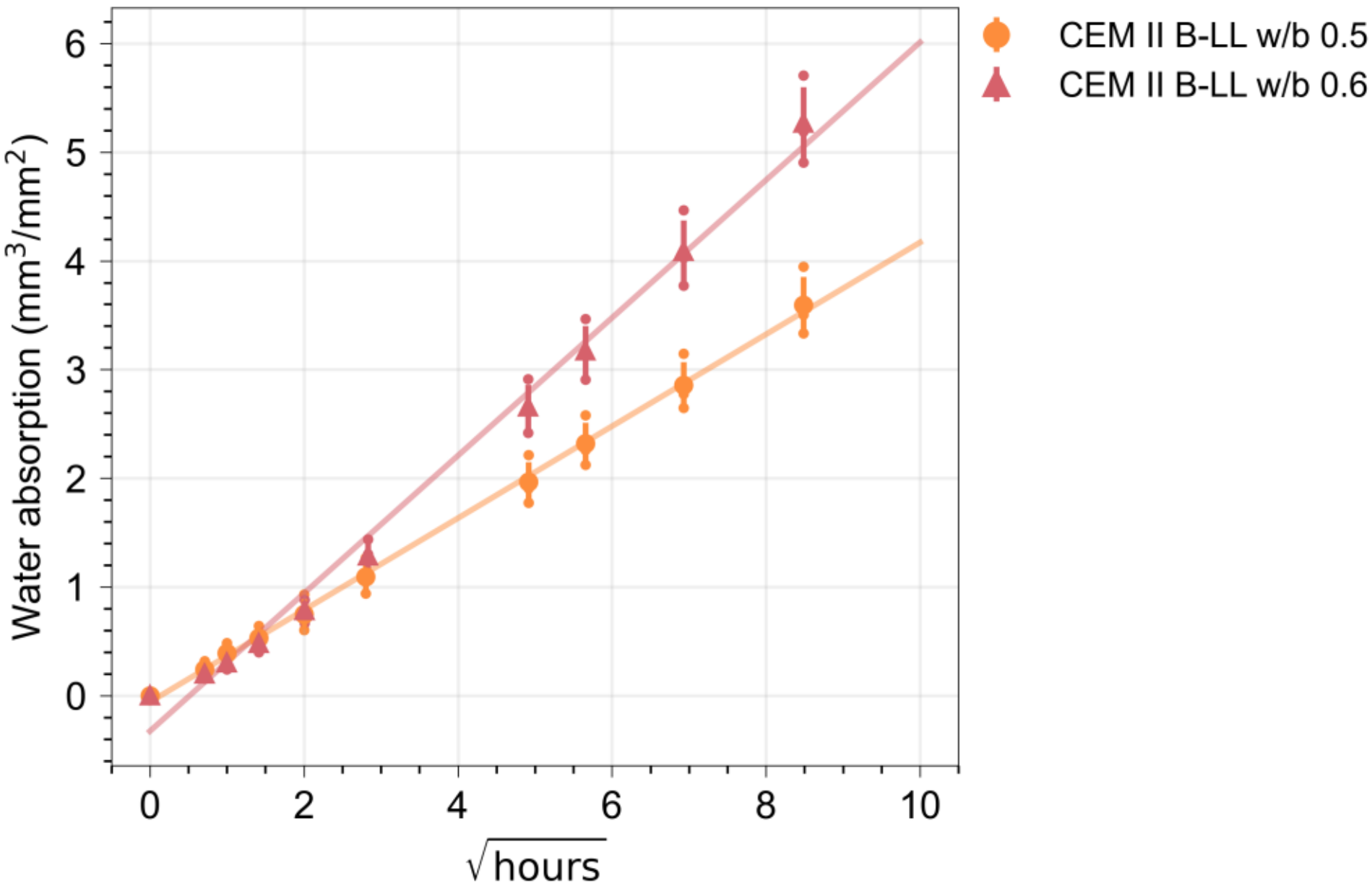


*Supplementary Figure 6 – Capillary absorption curves for carbonated CEM II B-LL w/b 0.5, and CEM II B-LL w/b 0.6 mixes. Error bars represent ± one standard deviation of 3 measurements. Source data are provided as a Source Data file.*

## Supplementary Note 5 – Sorption isotherms

The sorption isotherms of the mortar samples were characterized after carbonation (4% $CO_2$, 60±3% RH). Specimens of 40x40x160 mm³ were cut in thin slices of 40x40x3 mm³ employing an Accutom-50 cutting machine. Part of the specimens followed an adsorption path and part a desorption path. The lowest RH and degree of saturation (=0) were considered for specimens kept inside an oven at 65°C until mass equilibrium (< 0.0005% / min, approx. 3 weeks). The highest RH (=100%) and degree of saturation (=1) were considered for specimens saturated with deionized water and undergoing a vacuum protocol, with repeatedly applying vacuum (up to 0.15 bar) during 20 min and releasing it for 3 times. Intermediate RH conditions were achieved by storing the samples in desiccators with saturated salt solutions defining RH of 22%, 33%, 44%, 53%, 75%, 85%, and 97% until mass equilibrium (< 0.0005% / min, approx. 3 months). The saturation was calculated by interpolating the mass gain compared to the mass at 0% and 100% RH. The sorption isotherms are shown in Supplementary Figure 7.

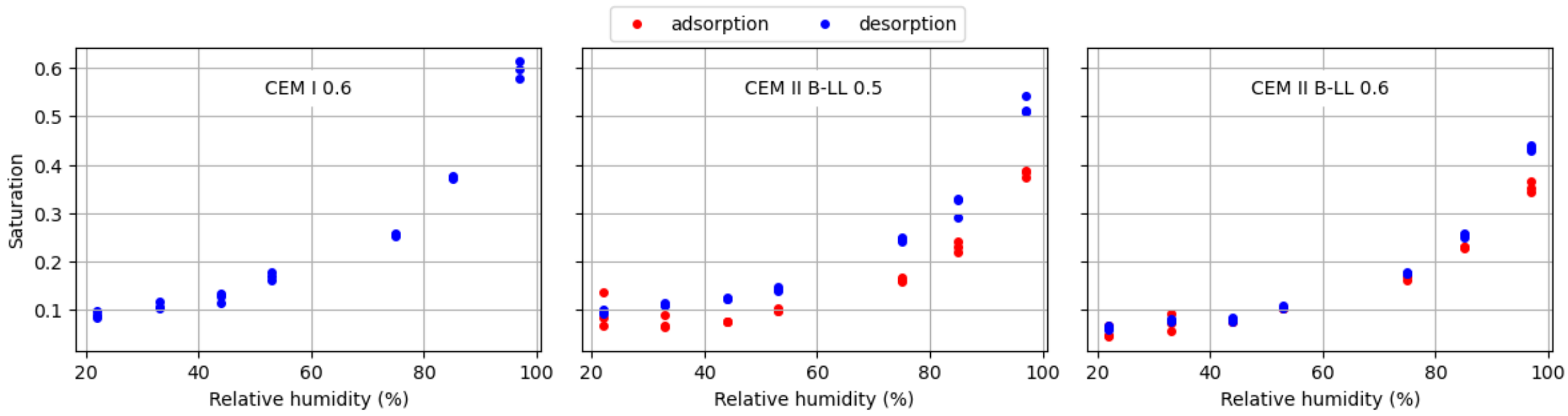


*Supplementary Figure 7 – Sorption isotherms showing the mortar saturation degree vs. RH for carbonated CEM I w/b 0.6, CEM II B-LL w/b 0.5, and CEM II B-LL w/b 0.6 mixes. Source data are provided as a Source Data file.*

## Supplementary Note 6 – Moisture transport simulations

The moisture transport model described in the main manuscript text requires the sorption isotherms as a model input. The sorption isotherms including desorption and adsorption curves were measured by the saturated salt solution method as described in Supplementary Note 5. The measured sorption isotherms were then fitted with the van Genuchten equation [36], as shown in Supplementary Figure 8a. The two fitting parameters $\alpha$ and $m$ in the van Genuchten equation for the studied materials are provided in Supplementary Table 3.

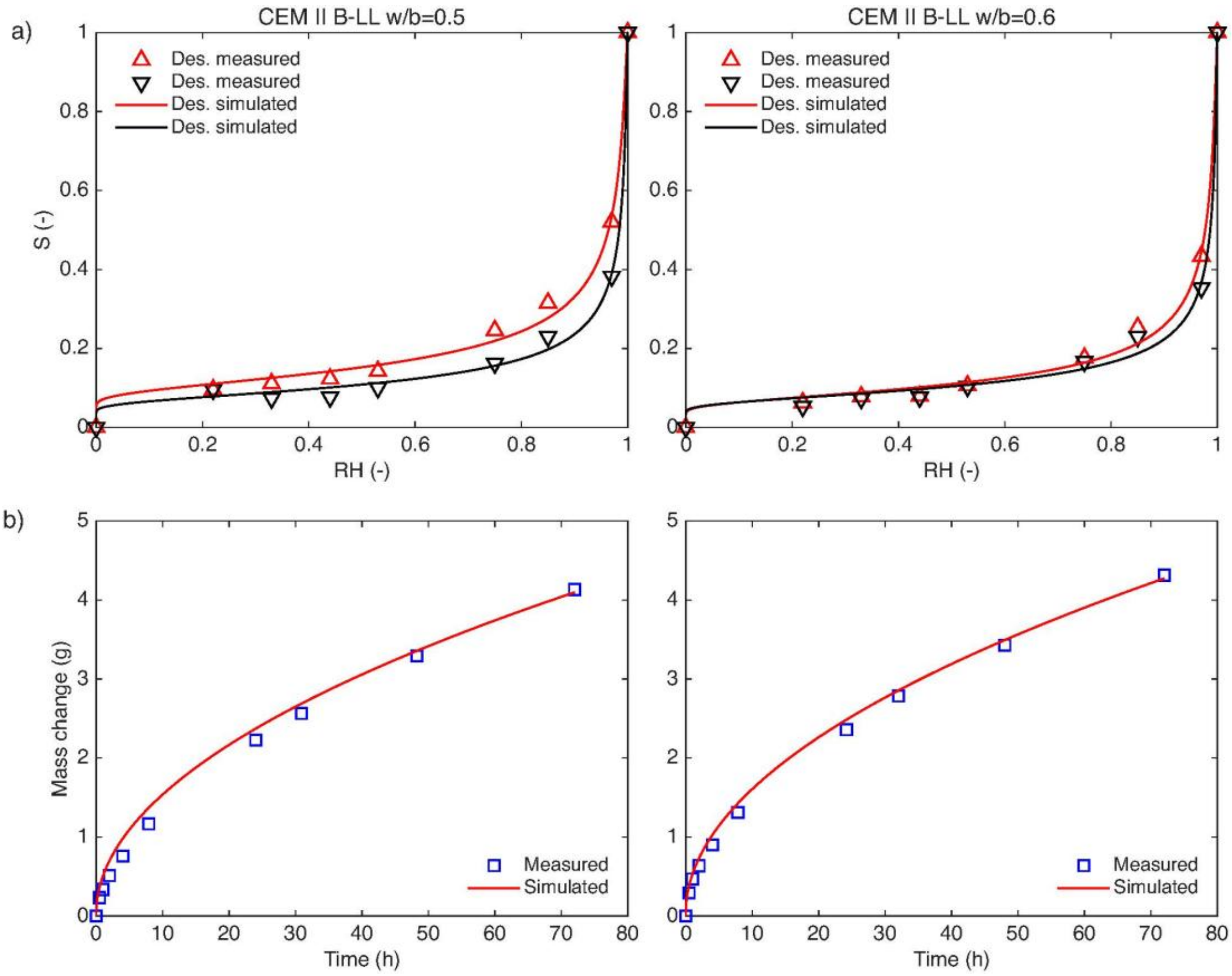


*Supplementary Figure 8 – Examples for properties for carbonated CEM II B-LL w/b=0.5 and w/b=0.6 mortars: a) sorption isotherms relating Saturation (S) and Relative Humidity (RH), and b) water absorption curves. Source data are provided as a Source Data file.*

Another key parameter for the moisture transport model is the intrinsic permeability $K_l$, which was determined by inverse modelling, which is a common approach in the literature[37]. This procedure treats $K_l$ as the only unknown in the model simulating the mass increase during a water absorption test. Supplementary Figure 8b shows that the obtained $K_l$ allowed for a good match between measured and simulated water absorption curves for CEM II B-LL w/b 0.5 and 0.6. The literature data show the values of $K_l$ for CEM B-LL w/b 0.6 range from $2.9\times10^{-20}$ to $8.6\times10^{-20}$ $m^2$ [38], which are very close to the obtained values in this study. Since we do not have the measured water absorption curve for CEM I w/b 0.6, the value of $K_l$ was estimated based on data for similar materials from the literature[39,40].

*Supplementary Table 3 - Parameters for numerical simulations of moisture transport in carbonated concrete*

| Parameter | Unit | CEM I w/b 0.6 | CEM II B-LL w/b 0.5 | CEM II B-LL w/b 0.6 |
|---|---|---|---|---|
| $K_l$ | $m^2$ | $1.05\times10^{-20}$ | $190\times10^{-20}$ | $235\times10^{-20}$ |
| $\alpha$ | Pa | $1.68\times10^{7}$ | $0.044\times10^{7}$ | $0.036\times10^{7}$ |
| $m$ | - | 0.310 | 0.293 | 0.289 |

With these calibrated parameters, we can employ the moisture transport model to simulate moisture transport in concrete when it is exposed to the weather conditions of the four selected geographic locations. The RH in these locations served as the boundary condition for the simulations. The simulation ran for multiple years until the yearly change patterns of saturation profiles for two consecutive years were similar, and the simulated results of the last year are shown in Supplementary Figure 9, which clearly displays the effect of weather conditions on moisture transport.

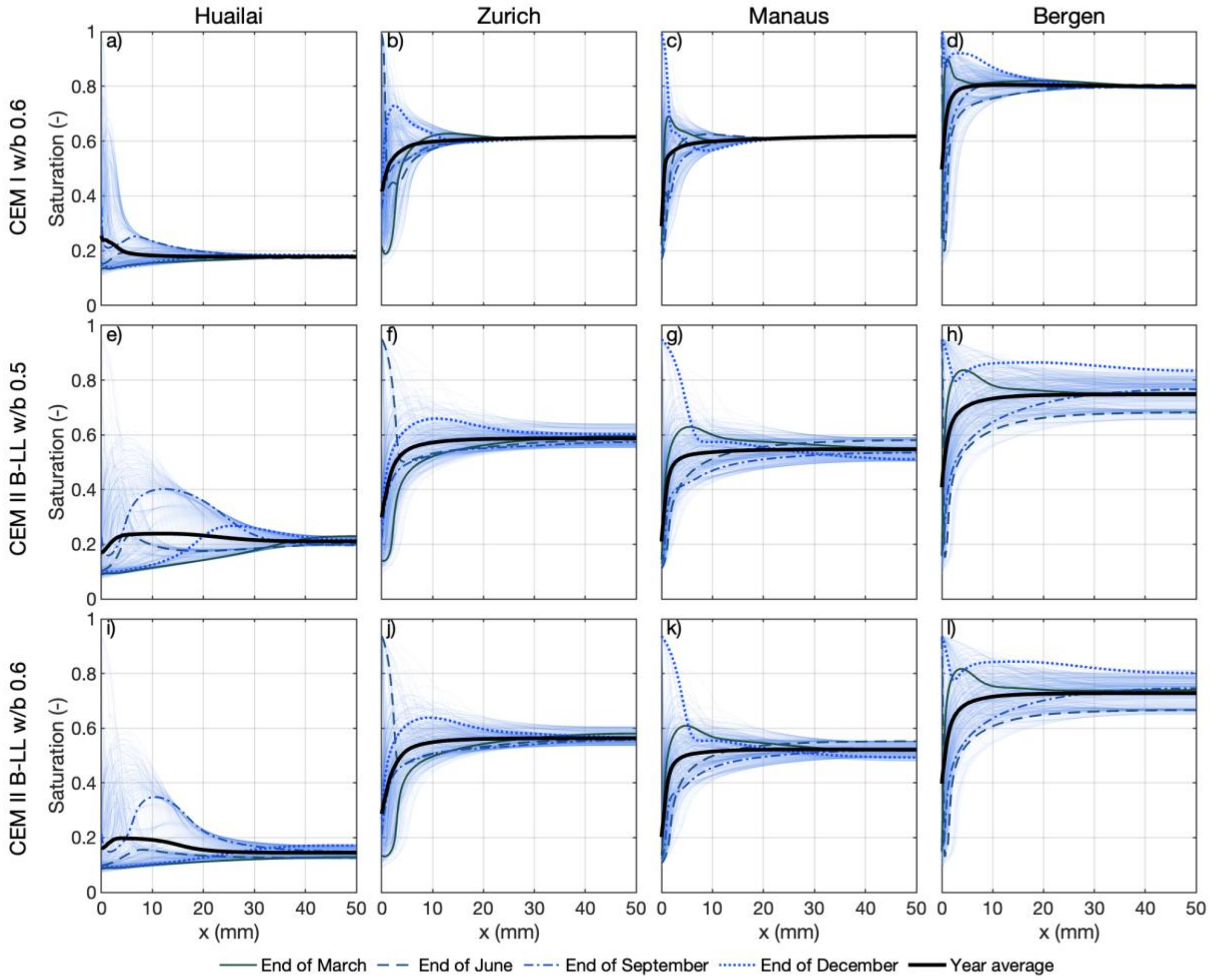


*Supplementary Figure 9 – Simulated saturation profiles throughout the year in carbonated concretes with different cement types, w/b ratios, and exposed to different weather conditions, as a function of the cover depth (x). Each thin line shows the corrosion rate at a specific day throughout the year; the blue thick line shows corrosion rates at the end of a quarter; the black thick line stands for the average saturation of the year. Source data are provided as a Source Data file.*

## Supplementary Note 7 – Modelled saturation and corrosion rates as a function of environmental RH

### *CEM I w/b 0.6*

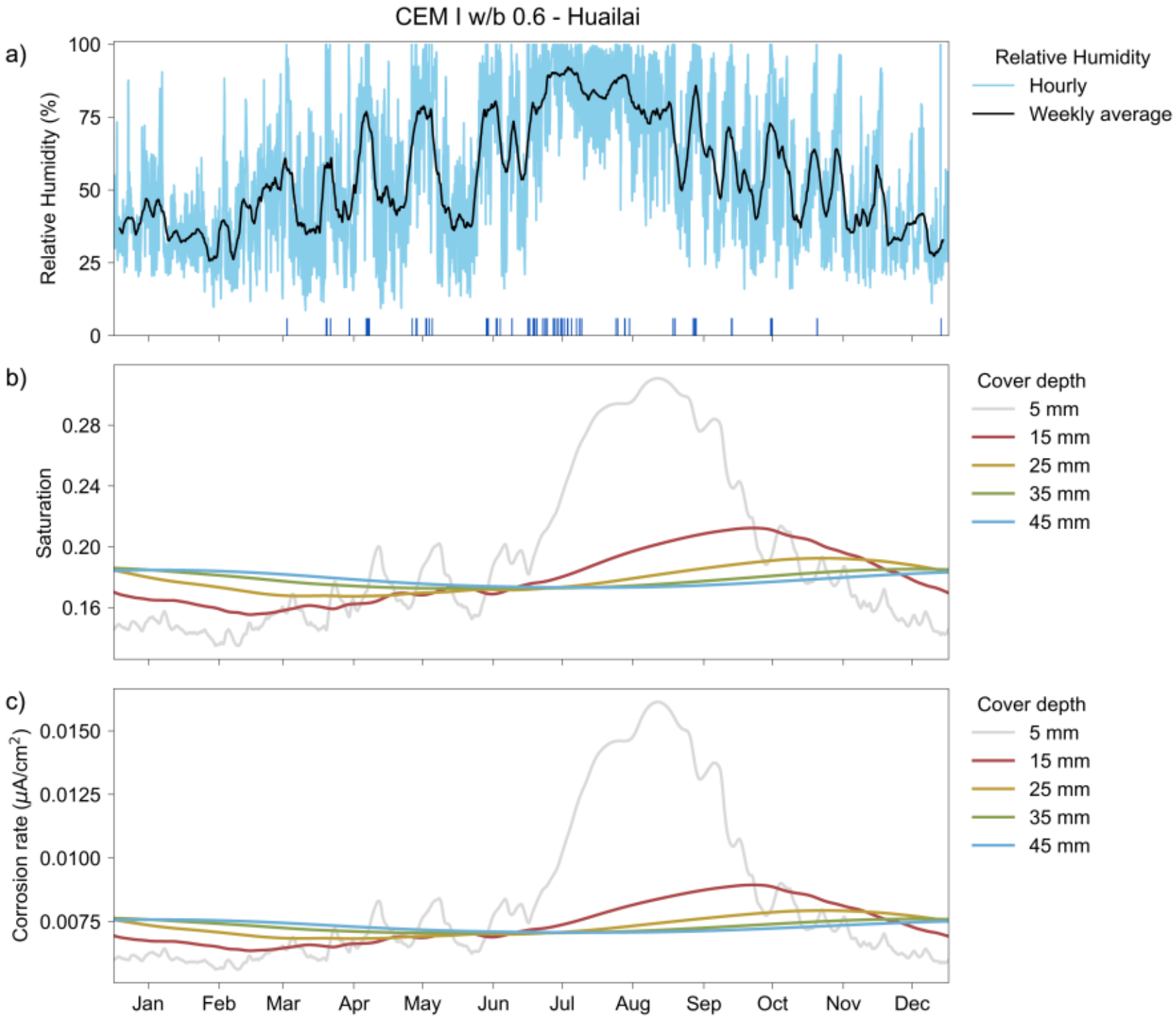


*Supplementary Figure 10 – Overview of the modelled annual saturation and corrosion rates of steel in a concrete produced with CEM I, w/b 0.6 as a function of exposure conditions in Huailai. Source data are provided as a Source Data file.*

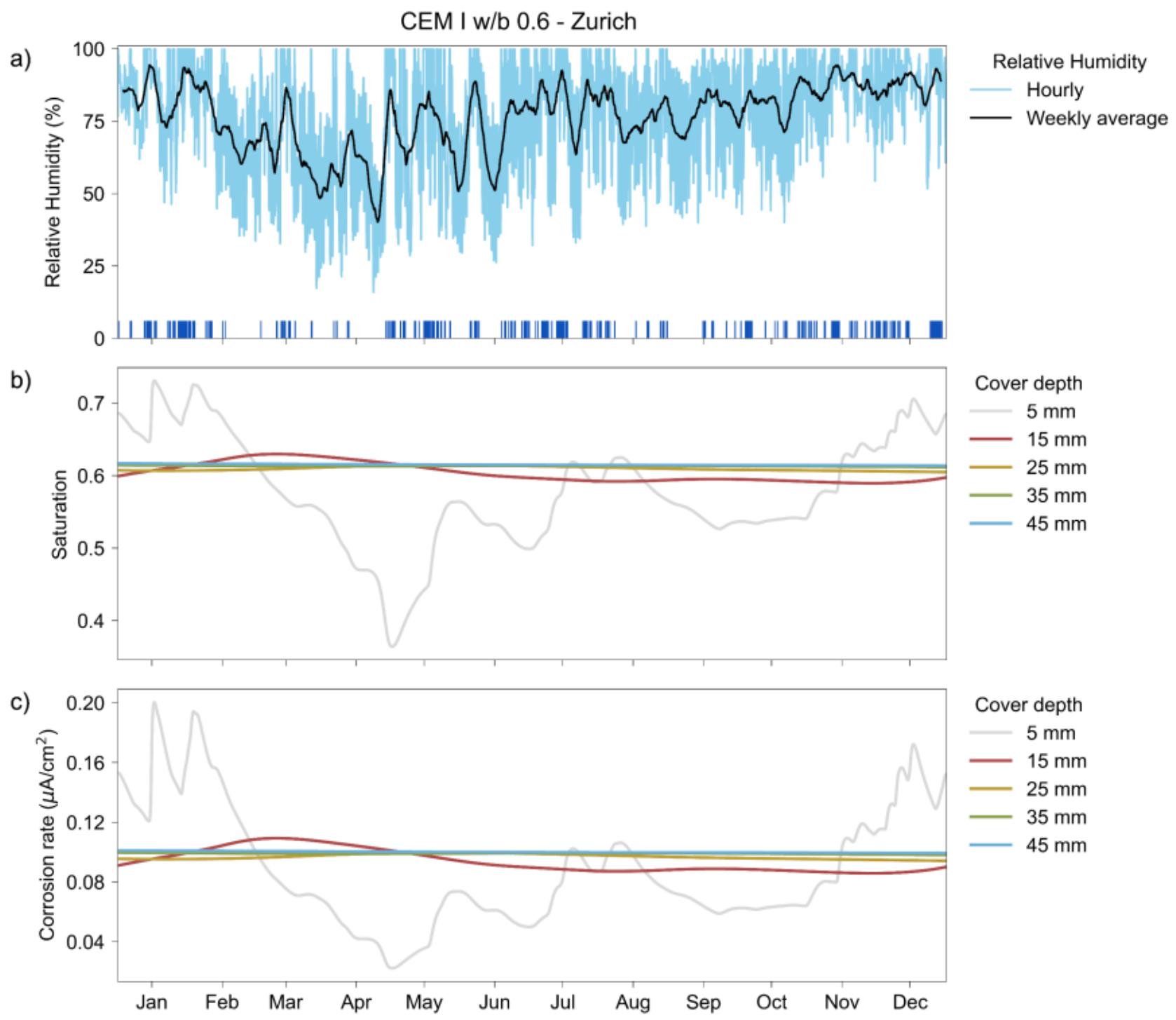


*Supplementary Figure 11 – Overview of the modelled annual saturation and corrosion rates of steel in a concrete produced with CEM I, w/b 0.6 as a function of exposure conditions in Zurich. Source data are provided as a Source Data file.*

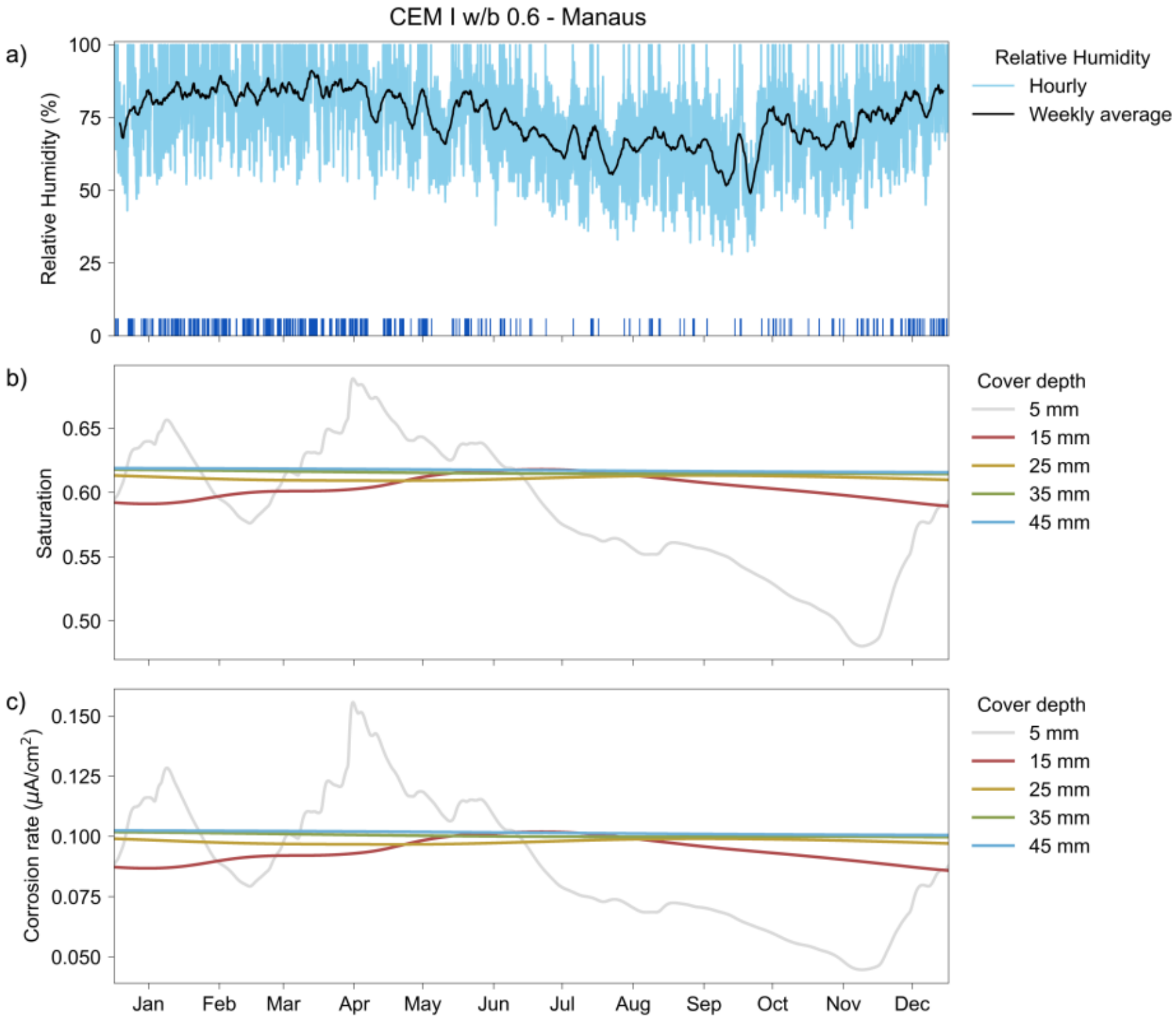


*Supplementary Figure 12 – Overview of the modelled annual saturation and corrosion rates of steel in a concrete produced with CEM I, w/b 0.6 as a function of exposure conditions in Manaus. Source data are provided as a Source Data file.*

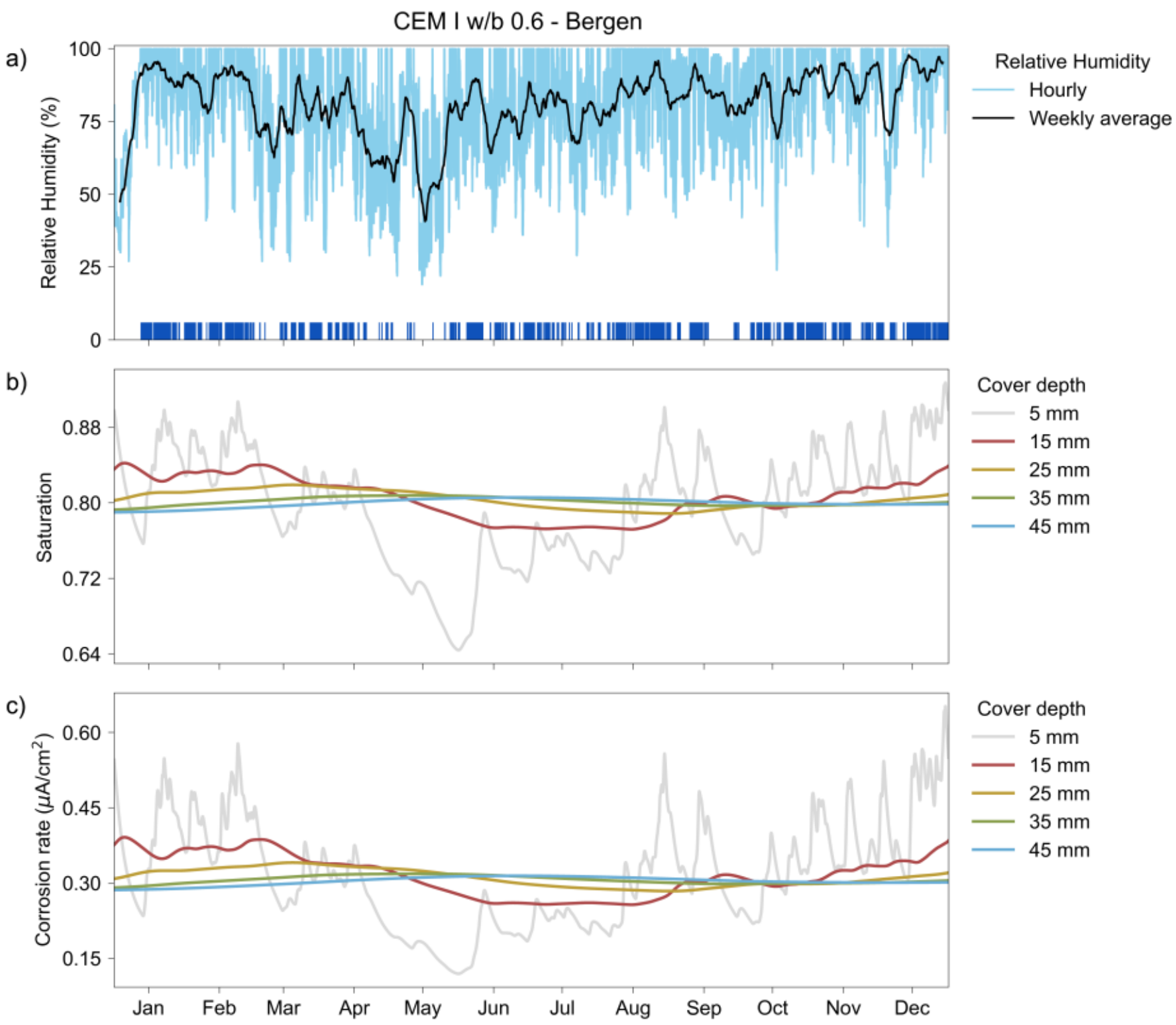


*Supplementary Figure 13 – Overview of the modelled annual saturation and corrosion rates of steel in a concrete produced with CEM I, w/b 0.6 as a function of exposure conditions in Bergen. Source data are provided as a Source Data file.*

## *CEM II B-LL w/b 0.5*

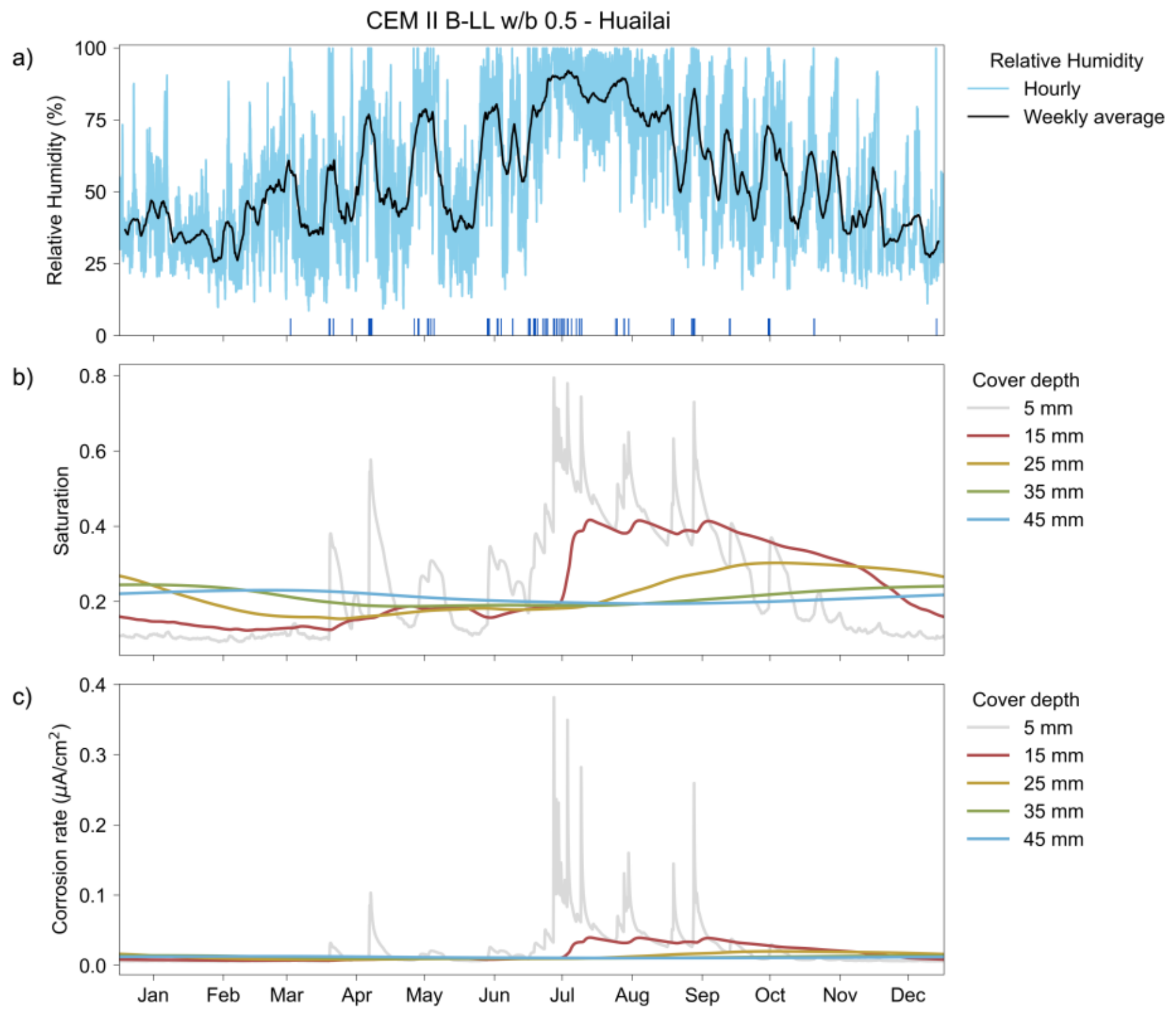


*Supplementary Figure 14 – Overview of the modelled annual saturation and corrosion rates of steel in a concrete produced with CEM II B-LL, w/b 0.5 as a function of exposure conditions in Huailai. Source data are provided as a Source Data file.*

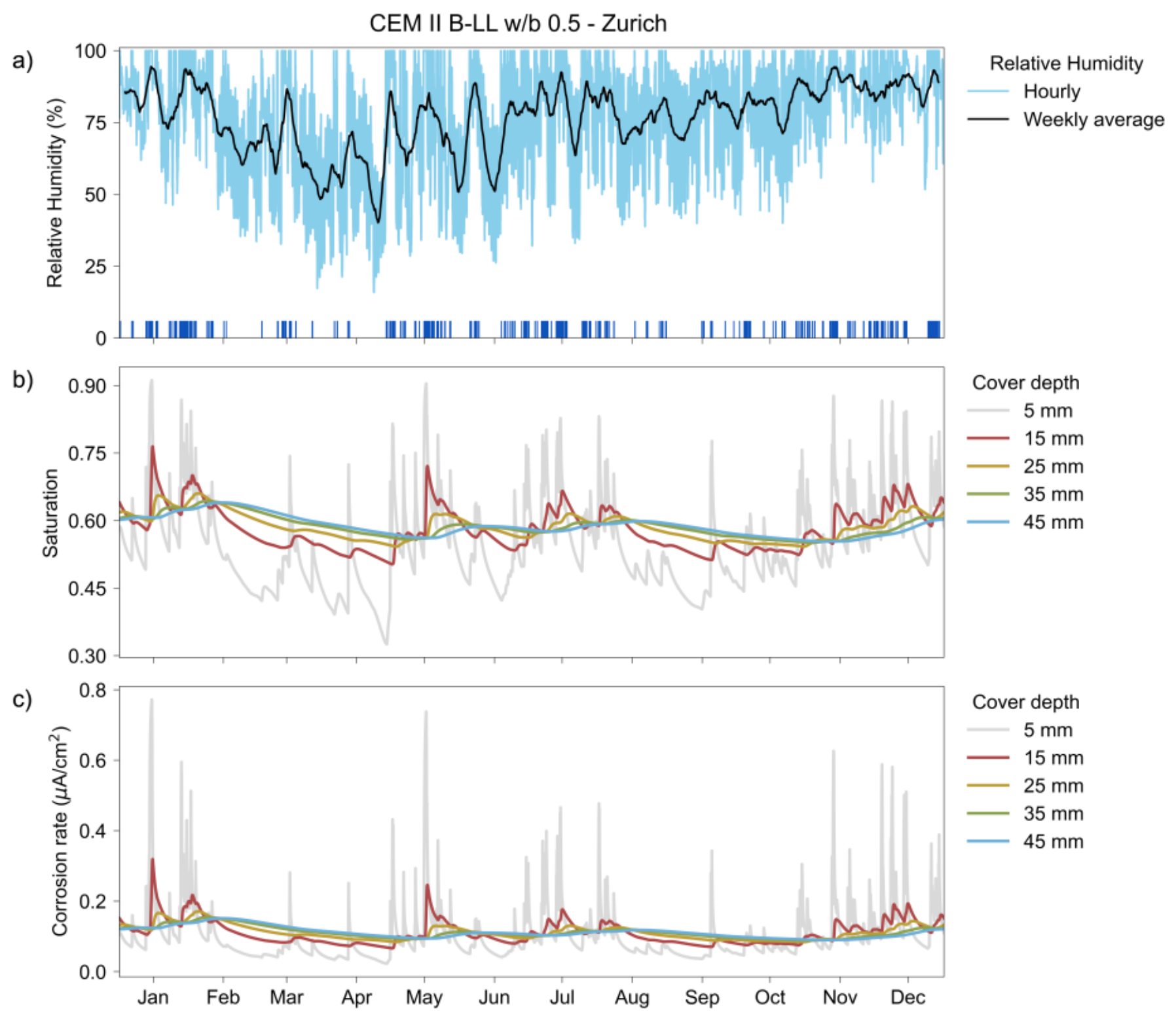


*Supplementary Figure 15 – Overview of the modelled annual saturation and corrosion rates of steel in a concrete produced with CEM II B-LL, w/b 0.5 as a function of exposure conditions in Zurich. Source data are provided as a Source Data file.*

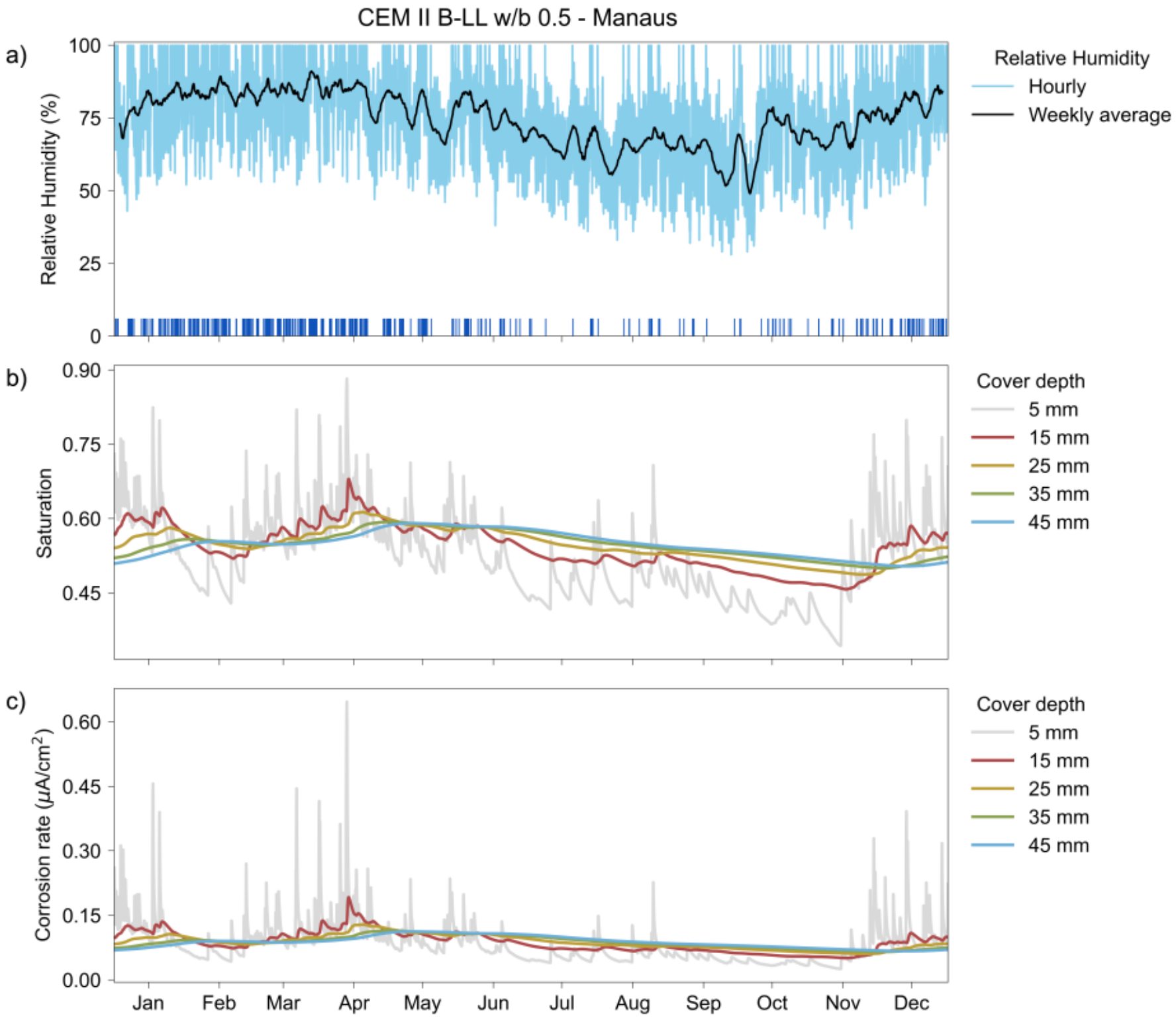


*Supplementary Figure 16 – Overview of the modelled annual saturation and corrosion rates of steel in a concrete produced with CEM II B-LL, w/b 0.5 as a function of exposure conditions in Manaus. Source data are provided as a Source Data file.*

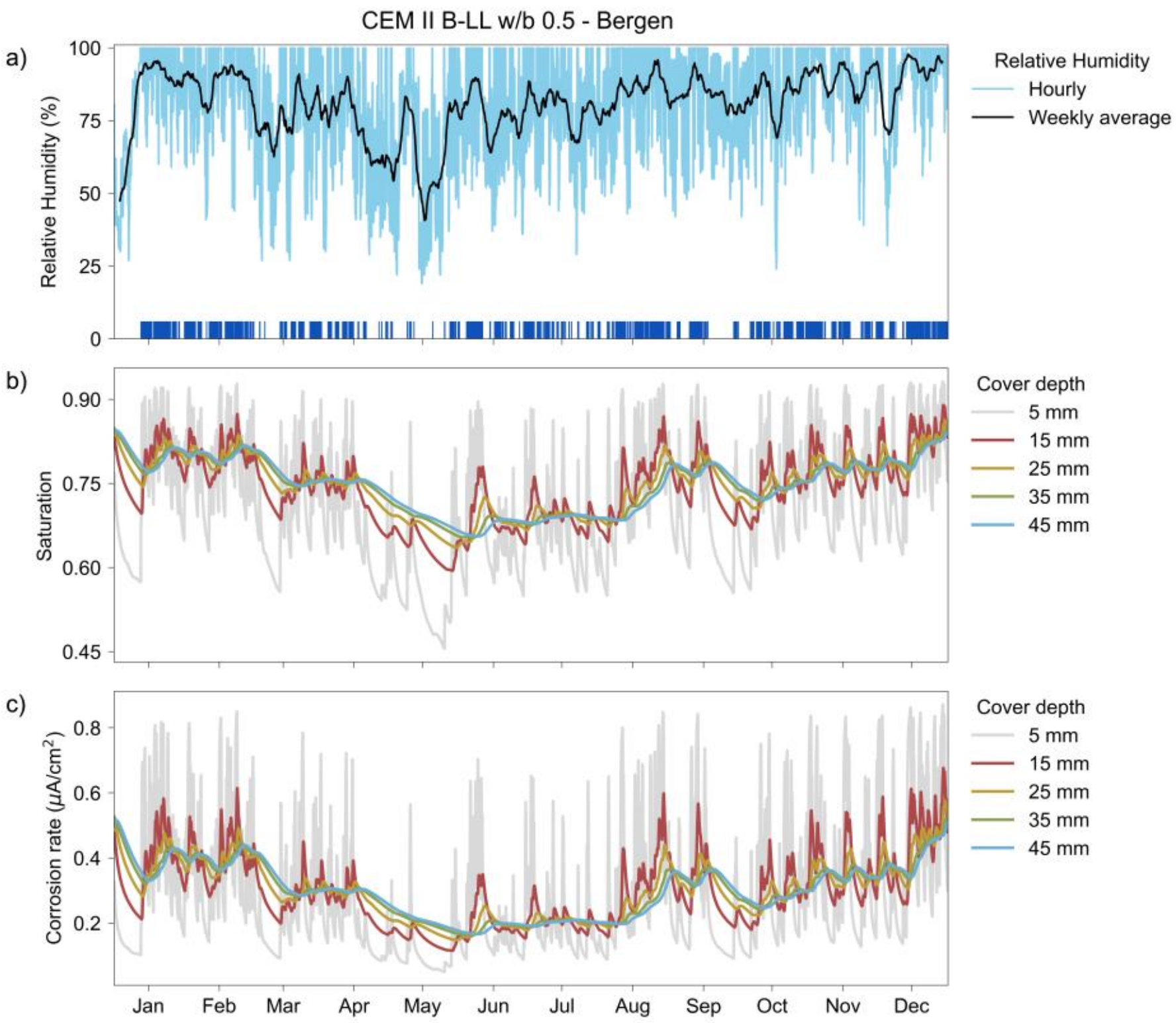


*Supplementary Figure 17 – Overview of the modelled annual saturation and corrosion rates of steel in a concrete produced with CEM II B-LL, w/b 0.5 as a function of exposure conditions in Bergen. Source data are provided as a Source Data file.*

## *CEM II B-LL w/b 0.6*

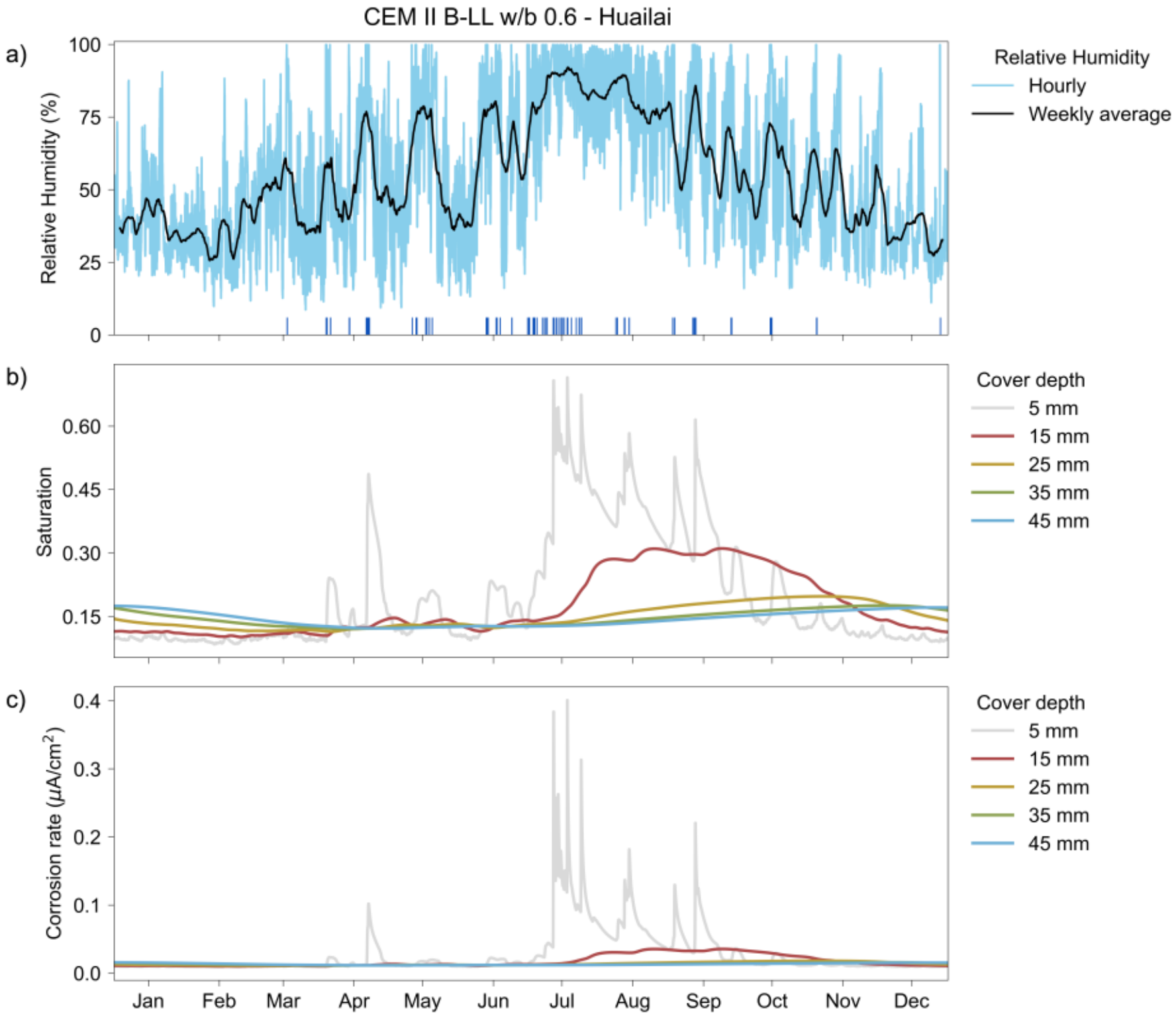


*Supplementary Figure 18 – Overview of the modelled annual saturation and corrosion rates of steel in a concrete produced with CEM II B-LL, w/b 0.6 as a function of exposure conditions in Huailai. Source data are provided as a Source Data file.*

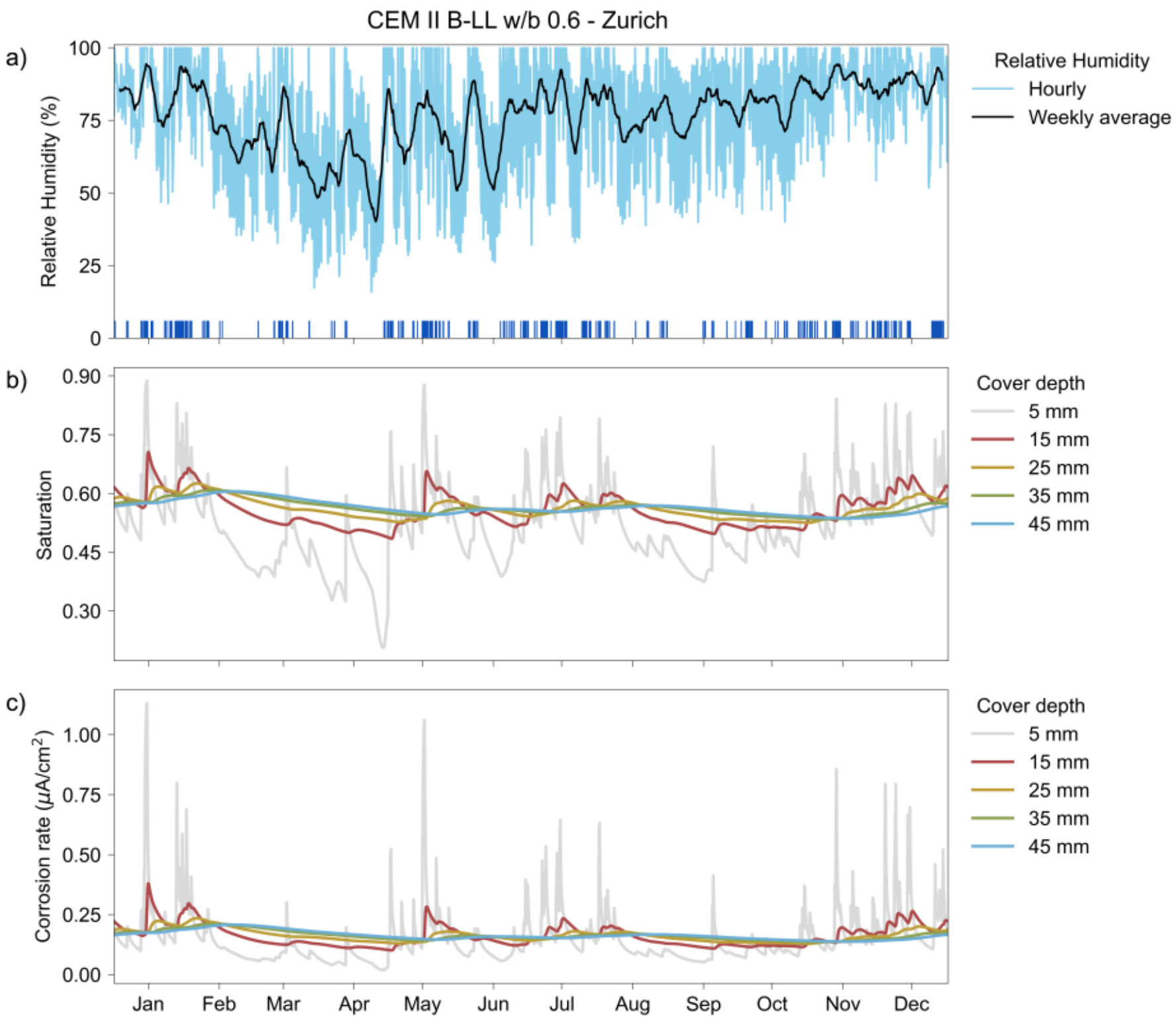


*Supplementary Figure 19 – Overview of the modelled annual saturation and corrosion rates of steel in a concrete produced with CEM II B-LL, w/b 0.6 as a function of exposure conditions in Zurich. Source data are provided as a Source Data file.*

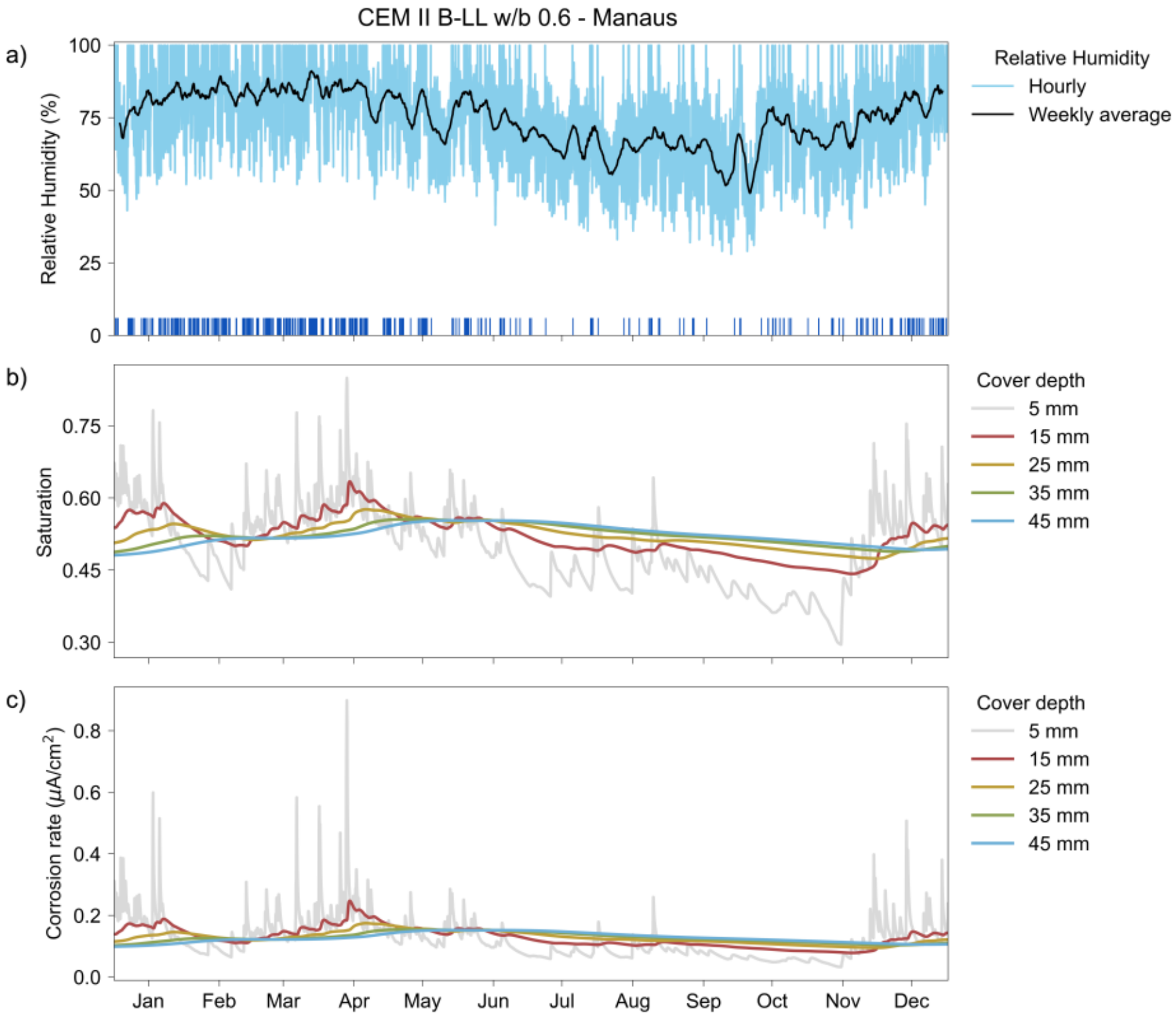


*Supplementary Figure 20 – Overview of the modelled annual saturation and corrosion rates of steel in a concrete produced with CEM II B-LL, w/b 0.6 as a function of exposure conditions in Manaus. Source data are provided as a Source Data file.*

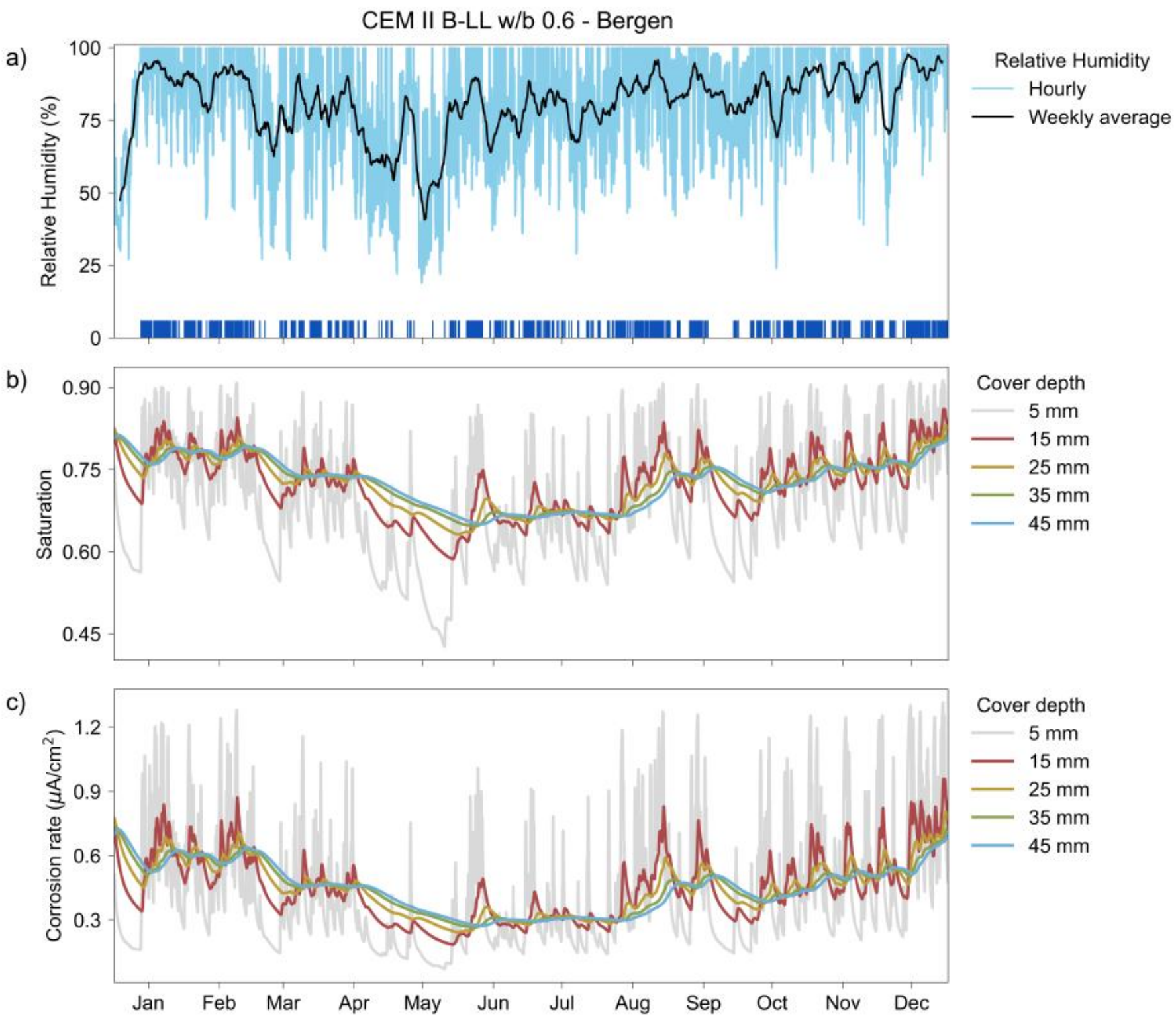


*Supplementary Figure 21 – Overview of the modelled annual saturation and corrosion rates of steel in a concrete produced with CEM II B-LL, w/b 0.6 as a function of exposure conditions in Bergen. Source data are provided as a Source Data file.*

## Supplementary Note 8 – Corrosion rates as a function of degree of saturation of the concrete

Literature data on corrosion rates of steel in carbonated concrete is commonly presented as a function of the exposure relative humidity (RH), generally upon equilibration[41,42]. To relate the degree of saturation in concrete, as obtained from moisture transport modelling, to corrosion rates of the embedded steel, a conversion between RH and degree of saturation is required, which in turn necessitates the use of sorption isotherms. This section describes the literature data employed for this purpose and highlights current limitations in the state of the art.

We analysed available literature data on instantaneous corrosion rates of carbon steel in carbonated CEM I w/b 0.5 and 0.6 concrete equilibrated at different RH[43–45]. Since the sorption isotherms of these cementitious materials were not available in the respective studies, the indicated exposure RH was here converted into degree of saturation of the concrete by applying two sorption isotherms: (a) the sorption isotherm of CEM I measured in this study (Supplementary Figure 7), and (b) the sorption isotherm of CEM I reported in Ren et al.[46]. Note that there is limited literature data on moisture sorption isotherms in *carbonated* (as opposed to uncarbonated) concrete, which presents a challenge in converting exposure RH into degree of concrete saturation and highlights the need for further research in this respect.

The estimated degree of saturation was then related to the corresponding instantaneous corrosion rate of steel as shown in Supplementary Figure 22. Due to the pronounced non-linear relationship, the data were described using an exponential function as given in Equation 7 of the manuscript, with $\mathrm{k}_{icorr\ cem}$ = 1 for CEM I w/b 0.6. To account for the tendency reported in the literature that CEM II may exhibit higher corrosion rates than CEM I, and to avoid overestimating the performance of CEM II within the current state of knowledge, conservative adjustments were made as follows: $\mathrm{k}_{icorr\ cem}$ = 1.3 for CEM II w/b 0.5 and $\mathrm{k}_{icorr\ cem}$ = 2.2 for CEM II w/b 0.6, on the basis of the ratio of $i_{corr}$ between carbonated CEM II mixes and carbonated CEM I w/b 0.6, at high RH conditions (99%) or full saturation[43,44,47].

It should also be noted that, given the limited data available in the literature, average values were in Equation 7 adopted instead of fractiles, as the latter could not be reliably estimated on the basis of the available dataset. The use of average values is considered appropriate for the purpose of illustrating the proposed concept. However, it should be noted that these values are not conservative and therefore do not represent a safety-oriented design basis.

The here presented data, as well as Equation 7 in the manuscript, should be considered as the best available knowledge to date. However, these are subject to uncertainties that should be addressed in future research (see Methods section "Corrosion rates of steel in cementitious materials" of the manuscript for further details).

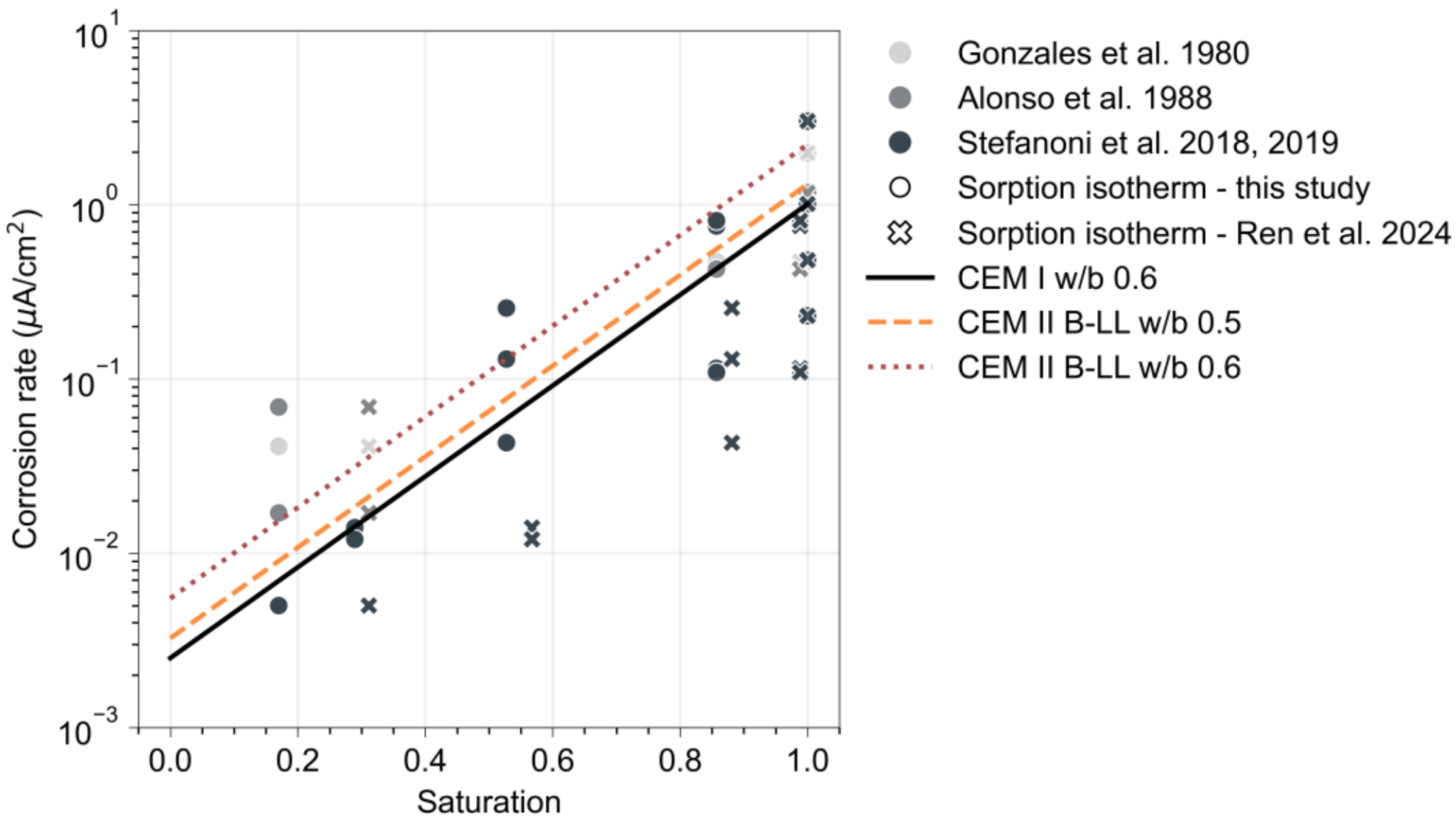


*Supplementary Figure 22 – Corrosion rates as a function of saturation, based on Portland cement concretes with w/b 0.6 from literature[43–45], and converting RH to saturation utilizing sorption isotherms of this study and of Ren et al.[46]. Source data are provided as a Source Data file.*

## Supplementary Note 9 – In search of simple durability predictors

Direct correlations between corrosion rates and climate parameters reveal general trends (Supplementary Figure 23). Higher RH and increased precipitation days (> 1mm) (Supplementary Figure 23a,c) lead to higher concrete saturation and increased average corrosion rates of steel. On the other hand, the total annual precipitation seems to play a secondary role (Supplementary Figure 23b). A lower monthly PCI, i.e., more homogeneous precipitation volumes over time, suggests an increasing corrosion rate (Supplementary Figure 23d). However, the PCI values are relatively similar for Zurich, Manaus and Bergen, not allowing to adequately distinguish these climates and their influence on corrosion rates. Despite such trends, no single climate parameter fully captures the complexity of the climate influence on concrete saturation and corrosion rates of steel, showing the relevance of considering seasonal variations and the duration and distribution of rain events.

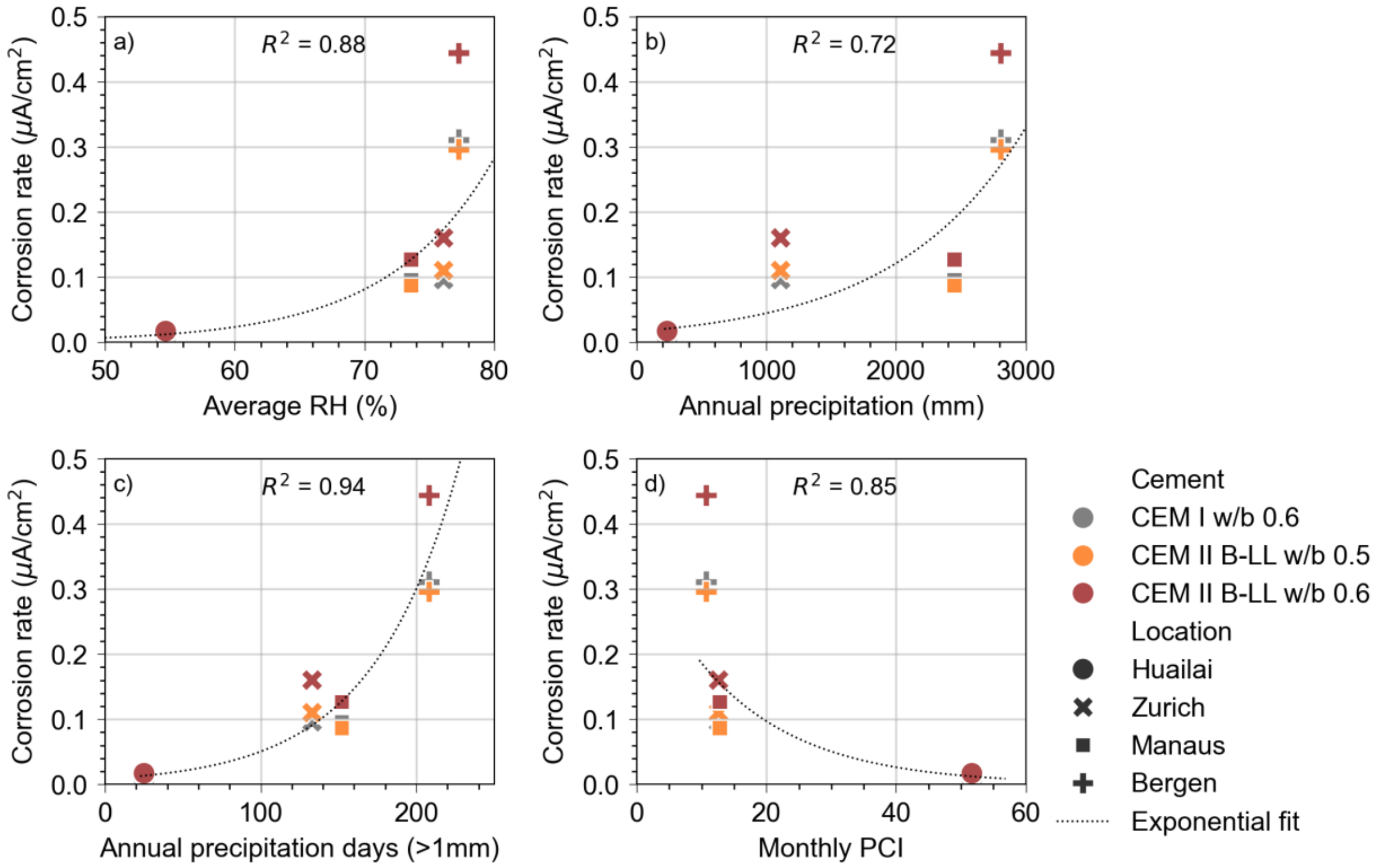


*Supplementary Figure 23 – Average annual corrosion rate versus selected meteorological and climate indicators. The data shows that the complex, time-dependent moisture transport processes within carbonated concrete leading to steel corrosion under actual exposure conditions cannot be captured by a single predictive parameter. Source data are provided as a Source Data file.*

## Supplementary Note 10 – Comparison of temperature and moisture effects on the corrosion rate

In a previous research project[48] (link to publication in footnote[A]), different exposure-related and material-related factors on the instantaneous corrosion rate of steel in carbonated concrete were investigated, which allows for a quantitative comparison of the effects of temperature and moisture condition on the instantaneous corrosion under otherwise identical experimental conditions. Specifically, corrosion rates of carbon steel in different concretes, produced from a range of different binders and water/binder ratios, were measured.

The concrete's moisture state was in Ref.[48] found to be the by far most pronounced influencing parameter. When changing the moisture state of the concrete from equilibrium at 95% relative humidity to a condition where the concrete was in contact with liquid water, the measured corrosion rates varied by a factor of 10. In actual exposure conditions, the moisture state can vary over significantly greater ranges (e.g. 20–100% RH as for the meteorological data considered in the present study, see Supplementary Note 2). Hence, in agreement with literature[41], it is common to find variability by a factor >100 in terms of instantaneous corrosion rates under such variable conditions.

On the other hand, when temperature was varied between 5 and 40°C, the corrosion rate was measured to vary only by a factor of less than 3 in Ref.[48]. This corresponds to approximately a factor of 1.2–1.5 in corrosion rate per 10°C temperature variation. This result is consistent with findings from other studies in reinforced mortars[49–51] as well as for carbon steel immersed in seawater[52] or in acidic electrolytes[53], showing an increase in corrosion rates by a factor of approx. 2 (in rare cases 3) for every 10°C increase. As is apparent from Supplementary Note 2, for the here considered climates, the temperature varied within a maximum daily range of approx. 10°C (Manaus), 15°C (Bergen, Zurich), or 20°C (Huailai).

Hence, for corrosion of steel in concrete under practical conditions the moisture state of the concrete is a significantly more dominant factor than temperature, with an influence that is at least one to two orders of magnitude greater.

Moreover, in the context of climate change, we consider the raise in temperature by 2–3°C on the earth's surface to have a negligibly small effect on the corrosion-related deterioration for steel in concrete compared to the effects related to changes in precipitation patterns.

For these reasons, in the present work, we prioritised the moisture state as the primary factor and left temperature effects for further consideration.

[A] https://www.cemsuisse.ch/app/uploads/2021/11/201807_Schlussbericht-komplett_NEU-2.pdf

## Supplementary References


1. SIA. SN EN 206:2013+A2:2021 Concrete – Specification, Properties, Manufacturing and Conformity. Switzerland. (2021).
2. SIA. SIA 262/1:2019 Concrete Structures – Supplementary specifications. Switzerland. (2019).
3. DIN. DIN EN 206-1 Concrete – Specification, performance, production and conformity. Germany. (2024).
4. DIN. DIN 1045-2 Concrete, reinforced and prestressed concrete structures – Part 2: Concrete. Germany. (2023).
5. BSI. BS 8500-1:2015+A2:2023 Concrete – Complementary British Standard to BS EN 206. United Kingdom. (2023).
6. BSI. BSI Flex 350 v2.0:2024 - Alternative binder systems for lower carbon concrete. (2024).
7. NBN. NBN B15-001:2018 Béton - Spécification, performances, production et conformité. Belgium. (2018).
8. NBN. NBN B 15-100:2018, Concrete - Methodology for the assessment and the validation of the fitness for use of cements or additions for concrete. Belgium. (2018).
9. NS-EN 206:2013+A2:2021. Concrete – Specification, Properties, Manufacturing and Conformity. Norway. (2021).
10. NS-EN 12390-10:2018. Determination of the carbonation resistance of concrete at atmospheric levels of carbon dioxide. Norway. (2018).
11. DS 206:2025. Concrete – Specification, performance, production and conformity – Rules for application of EN 206 in Denmark. (2025).
12. NT BUILD 357:1989. Concrete, repairing materialsand protective coating: Carbonation resistance. Norway. (1989).
13. Ministerio de Fomento. Code on Structural Concrete (EHE-08). Spain. (2008).
14. LNEC. E464 Betões. Metodologia prescritiva para uma vida útil de projeto de 50 e de 100 anos face às ações ambientais. Portugal. (2007).
15. LNEC. NP EN 1992-1-1/NA:2010 Eurocódigo 2 – Projecto de estruturas de betão. Portugal. (2010).
16. ABNT. NBR 6118 Design of concrete structures. Brazil. (2023).
17. ABNT. NBR 12655 Portland cement concrete - Preparation, control, receipt and acceptance - Procedure. Brazil. (2015).
18. ABNT. NBR 15575-2 Residential buildings - Performance Part 2: Requirements for structural systems. Brazil. (2013).
19. ACI. ACI 318-19 Building Code Requirements for Structural Concrete. United States. (2019).
20. ACI. *ACI 222R-19 Guide to Protection of Reinforcing Steel in Concrete against Corrosion. United States.* (2019).
21. ACI. *ACI 365.1 R-17 Report on Service Life Prediction. United States.* (2017).
22. *GB/T 50476-2019: Standard for Design of Concrete Structure Durability. People's Republic of China.* (2019).
23. CCES 01-2004: Guide to Durability Design and Construction of Concrete Structures. People's Republic of China. (2004).
24. BIS. IS 456:2000 Plain and Reinforced Concrete - Code of Practice. India. (2000).
25. JSCE. JSCE 15 - Standard specifications for concrete structures: Design. Japan. (2007).
26. JSCE. JSCE 16 - Standard specifications for concrete structures: Materials and Construction. Japan. (2007).

27. Instituto Nacional de Meteorologia (INMET). Daily precipitation, Manaus Station (A101). (2000).

28. Federal Office of Meteorology and Climatology Meteoswiss, IDAWEB application,. https://gate.meteoswiss.ch/idaweb/login.do (2024).

29. Norwegian Centre for Climate Services. https://seklima.met.no/observations/ (2025).

30. Bai, J. *et al.* Meteorological Observation Dataset from HL_RS_TS. https://doi.org/10.3974/geodb.2020.09.12.V1.

31. Weatherspark. Climate and Average Weather Year Round in Bergen. https://weatherspark.com/y/52849/Average-Weather-in-Bergen-Norway-Year-Round.

32. Oliver, J. E. Monthly precipitation distribution: a comparative index. *Prof. Geogr.* **32**, 300–309 (1980).

33. CPC-18 Measurement of hardened concrete carbonation depth. *Mater. Struct.* **21**, 453–455 (1988).

34. Bernal, S. A. *et al.* Recommendation of RILEM TC 281-CCC: RILEM CPC-18R1—guideline for measuring the carbonation depth of hardened concrete using a pH indicator solution. *Mater. Struct.* **59**, 137 (2026).

35. Polder, R. *et al.* Test methods for on site measurement of resistivity of concrete. *Mater. Struct.* **33**, 603–611 (2000).

36. van Genuchten, M. T. A Closed-form Equation for Predicting the Hydraulic Conductivity of Unsaturated Soils. *Soil Sci. Soc. Am. J.* **44**, 892–898 (1980).

37. Zhang, Z., Thiery, M. & Baroghel-Bouny, V. Investigation of moisture transport properties of cementitious materials. *Cem. Concr. Res.* **89**, 257–268 (2016).

38. Zhang, Z. & Angst, U. Microstructure and moisture transport in carbonated cement-based materials incorporating cellulose nanofibrils. *Cem. Concr. Res.* **162**, 106990 (2022).

39. Song, H.-W. & Kwon, S.-J. Permeability characteristics of carbonated concrete considering capillary pore structure. *Cem. Concr. Res.* **37**, 909–915 (2007).

40. Ren, F., Chen, X., Zeng, Q. & Zhou, C. Effects of pure carbonation on pore structure and water permeability of white cement mortars. *CEMENT* **9**, 100040 (2022).

41. Stefanoni, M., Angst, U. & Elsener, B. Corrosion rate of carbon steel in carbonated concrete – A critical review. *Cem. Concr. Res.* **103**, 35–48 (2018).

42. Angulo Ramirez, D. E., Meira, G. R., Quattrone, M. & John, V. M. A review on reinforcement corrosion propagation in carbonated concrete – Influence of material and environmental characteristics. *Cem. Concr. Compos.* **140**, 105085 (2023).

43. Stefanoni, M., Angst, U. M. & Elsener, B. Electrochemistry and capillary condensation theory reveal the mechanism of corrosion in dense porous media. *Sci. Rep.* **8**, 7407 (2018).

44. Stefanoni, M., Angst, U. M. & Elsener, B. Kinetics of electrochemical dissolution of metals in porous media. *Nat. Mater.* **18**, 942–947 (2019).

45. Alonso, C., Andrade, C. & González, J. A. Relation between resistivity and corrosion rate of reinforcements in carbonated mortar made with several cement types. *Cem. Concr. Res.* **18**, 687–698 (1988).

46. Ren, F., Zhou, C., Zhang, Z., Angst, U. & Chen, X. Quantifying isothermal drying of cement mortars in view of water sensitivity. *Constr. Build. Mater.* **443**, 137593 (2024).

47. Lollini, F. & Redaelli, E. Corrosion rate of carbon steel in carbonated concrete made with different supplementary cementitious materials. *Corros. Eng. Sci. Technol.* **56**, 473–482 (2021).

48. Stefanoni, M., Elsener, B. & Angst, U. *Cemsuisse Forschungsprojekt „Sicherstellung Der Dauerhaftigkeit von Stahlbetonbauten Bei Karbonatisierung“*. (2020).

49. Baccay, M. A., Otsuki, N., Nishida, T. & Maruyama, S. Influence of Cement Type and Temperature on the Rate of Corrosion of Steel in Concrete Exposed to Carbonation. *Corrosion* **62**, 811–821 (2006).

50. Cheng, L. & Maruyama, I. A Prediction Method for the Corrosion Rate of Steel Rebar in Carbonated Mortar under Variable Environmental Conditions. *J. Adv. Concr. Technol.* **21**, 611–630 (2023).

51. Wan, L. S., Hirata, M., Oyamoto, T., Kanda, T. & Tan, K. H. Synergistic impact of temperature and pore saturation on corrosion in carbonated reinforced concrete. *Case Stud. Constr. Mater.* **22**, e04364 (2025).

52. Melchers, R. E. Effect of Temperature on the Marine Immersion Corrosion of Carbon Steels. *Corrosion* **58**, 768–782 (2002).

53. Konovalova, V. The effect of temperature on the corrosion rate of iron-carbon alloys. *Mater. Today Proc.* **38**, 1326–1329 (2021).
