# Supplementary material for: Rethinking concrete durability for low-carbon concretes through climate-informed corrosion modelling
Source: Nat Commun. 2026 Aug 21;17:9999. doi: 10.1038/s41467-026-76916-3 (PMC13590661; doi:10.1038/s41467-026-76916-3)
Supplement: Supplementary file 2 [file 41467_2026_76916_MOESM2_ESM.pdf]

nature portfolio

Peer Review File

Rethinking Concrete Durability for Low-Carbon Concretes through Climate-Informed Corrosion Modelling

Corresponding Author: Professor Ueli Angst

This file contains all reviewer reports in order by version, followed by all author rebuttals in order by version.

Version 0:

Reviewer comments:

Reviewer #1

(Remarks to the Author)
An interesting paper from he outset.
The group more broadly is globally significant, as they are currently the contemporary critical mass in the domain of advanced studies in the degradation of reinforced concrete structures.
The present study takes the lens (appropriately, given the contemporary importantce) of climate and emissions. This is not a common topic in Nature Comms - in spite of the global relevance. As such, it is important that works such as these are communicated broadly. On this basis, the work is I believe important and merits publication. I have a number of comments for consideration.

1) Writing style: I get the sense when writing for a general journal, the paper has drifted towards colloquial or editorial style, as opposed to focused on the science. Perhaps if the authors are aware that the paper merits publication based on the science, they will be more open to trimming superfluous and non-technical aspects of the narrative. Also, there are several atypical facets in the writing. For example, the Abstract should be one paragraph....not two. The writing style also is better for the reader if words such as 'we' are replaced by different sentence structures that put the subject at the core of the sentence, and not people.
(in fact, the second paragraph in the Abstract, could be the opening part of a consolidated paragraph, as it gives the reader what they need).

2) I appreciate there there is some nuance in the format required for the journal. However, I feel that the introduction provides a lot of background, that is broad, and then the reader needs to really 'catch up' when the original parts of the paper are emphasised. I believe it's possible to start the paper by critically jumping into the topic at hand.

3) The results and discussion are REALLY interesting, and, really important. At this stage, I could have infinite questions - but I will actually leave the edits to the authors. My main point here, is that the reader is likely to want to be able to grapple with the impactful findings based on what is known in the field already. For example, how do the findings relate to known structures (or models) in the literature (i.e. from other works?). Is there any value in briefly discussing the findings based on the fields knowledge of things like critical chloride thresholds? (and how does this study then impact what may be done by a practitioner?). I am aware the group themselves are leaders in the work around critical chloride thresholds - so are well placed to briefly elaborate the discussion.
Better still, can a visual plot be made that relates the climate focused work in the study, with other works over the recent decades?

I don't see any of the above comments as having a pre-determined correct response or edits. They are principally for the authors to take the perspective of the reader (in the writing) and the revelation of the wider impact (through correlation with known / established works) - in a manner that makes sense to the authors.

Reviewer #2

(Remarks to the Author)
This paper proposed a conceptual framework to predict the service life of carbonation-influenced reinforced concrete. This novelty is to utilize moisture transport modelling based on day-to-day climate date. Steel rebar corrosion, caused either by carbonation or chloride, is a complicated process, and certain assumptions must be made to provide explainable results. The work has potential impact as it provides a tool to analysis how climate conditions influence corrosion rate. This is beyond the conventional rebar corrosion study which are highly sample specific. Over I feel the work is worth publishing. I have the following questions that the authors need to address.
1. Homogeneous corrosion along rebar surface is assumed, which is ok for carbonation-induced corrosion. Temperature, as an important climate factor, is not considered to influence the correlation between RH and corrosion rate – how to validate this?
2. There seems to be an assumption that corrosion rate is only determined by the moisture condition at the vicinity of the steel concrete interface (SCI), i.e. the corrosion rate – moisture correlation is independent of the cover depth. Can this be validated? Would oxygen availability be a limiting factor when rebar is deeply covered?
3. Isn't the corrosion rate also related to the metallurgical nature of the steel? How is this considered in the model?
4. In the corrosion kinetics modelling, is the SCI considered to have an identical pore structure as the bulk concrete? There are works suggesting the SCI may have more porosity than bulk concrete (Horne, A.T., Richardson, I.G. and Brydson, R.M.D., 2007. Quantitative analysis of the microstructure of interfaces in steel reinforced concrete. Cement and Concrete Research, 37(12), pp.1613-1623.)
5. I like the idea of ToW for rebars, but what the author stated in line 238-245 seems to suggest that ToW is a misleading measure of the real corrosion kinetics, isn't it?
6. Please provide a zoomed-in inset for Fig 4(a). Is it possible to provide the 1st order derivative for Fig 4 in the appendix? This will be a nice representation of corrosion kinetics throughout a year.
7. Fig 5 is great, but the estimation of carbonation phase relies on Duracrete model which is not an original work of this paper. Duracrete model itself has a good number of assumptions and regressions. Can the author comment on whether Duracrete model improves or reduces the reliability of service-life prediction in this paper? My concern is that the Duracrete model could not capture the influence of daily moisture change as the propagation-phase model in this paper.
8. The current practical approaches indeed oversimplify the exposure conditions, but are friendly to normal concrete technicians. The author must explain how the proposed computational modelling method, which requires concrete property and climate data (daily based, not yearly averaged as the author mentioned) as input, can be readily used by the industry.
9. I fully agree with this: "Thus, climate-change induced shifts in precipitation patterns may pose a problem and may threaten the durability of eco-friendly, reinforced concrete structures much more than a temperature rise by 2-3 °C."
10. Since supplementary figure 6-8 and table 2 are from real measurement, the author should provide the mix design of the specimens.
11. The correlation between saturation and corrosion rate is a core input to the overall modelling. In section 4.3, I understand that a simplification is needed to complete this conceptual framework. However, the author should provide stronger justification to this relationship. In particular, why is this relationship independent of the cover depth? The author should also provide a perspective how this can be validated for a random concrete mixture in real practise.

Reviewer #3

(Remarks to the Author)
This study presented a framework for durability evaluation of concrete subjected to carbonation. The study is well written and relevant to current needs. However, there some aspects that are unclear and should be amended/added. Please see points below:

- L40: silica fume? That's very seldom used for clinker replacement and specifically used for durability / high performance concrete
- L42: I would not call these properties superior. Early-age heat of hydration reduction is beneficial for massive concrete structures but comes at a penalty of lower strength, which admittedly is a main blocker in the adoption of lower carbon concretes. Chloride diffusion and associated properties are more superior for SCMs, as is later age strength gain
- L54-56: reads like depassivation is the main reason the carbon steel is used
- L79: abandon is a strong word. I guess "relax the reliance" on carbonation front reaching the reinforcement for durability might be more straight forward
- L92-95: simply, the deemed-to-satisfy approach
- L96: UK too from BSI flex 350
- Supplementary information Table 1, for UK, "E4" is not a cement but a performance category. Suggest you list the E4 corresponding cements. Also UK has BSI Flex 350. Suggest the authors also consider, Ireland, Norway and Canada in the review.
- L106-L116. Agree that these are qualitative. But there are quantifications based on RH. Such as, works by Stefanie Von Greve-Dierfeld and other organisations etc. These need to be reported and acknowledged
- L146: CEM II/B-LL is not a composite cement but a Portland-limestone cement. Also declare the % limestone power and the two distinct w/b
- The basis on selection CEM I and CEM IIB LL for calculations need to be clarified.
- L192: CEM II/B-LL on its own is very hard to use in concrete at higher %, the w/b of 0.6 will practically make this concrete unusable and really vulnerable to deterioration.
- Figure 2: useful finding, but how reliable is the estimation?
- Figure 4: as in Figure 2 the relevant threshold for negligible corrosion rate is proposed as 0.1 μA/cm^2, it would be useful to

have an equivalent for section loss vs crack (acknowledging this depends on concrete strength, cover depth etc)
- Finally on perspectives, what should be more clearly discussed is the lack of on-site validation of the model. Have the authors tried to corelate their modelling results to evidence/measurements from actual structures in corresponding locations? This type of analysis is something that should be added to strengthen the validity of the approach.
- Good study overall and moves durability design/characterisation towards the right direction.

Reviewer #4

(Remarks to the Author)
This manuscript addresses a timely and relevant challenge at the intersection of sustainable construction and infrastructure durability, proposing a climate-informed corrosion modeling framework for low-carbon concretes (LCCs). The writing is clear and the topic is of undeniable significance. However, several structural issues and scientific concerns must be resolved before publication can be recommended.
The most critical structural issue is the unconventional placement of the Methods section after Results and Discussion. This impairs the reader's ability to critically evaluate the modeling approach, input parameters, and simulation limitations. Equally important, the manuscript lacks both a Conclusion and a Perspectives section, leaving the reader without any synthesis of findings, critical appraisal of limitations, or indication of future directions.
The Introduction requires substantial expansion. While it effectively contextualizes climate change and the need to reduce the carbon footprint of construction materials, several important dimensions are absent. No connection is drawn between global sea level rise and reinforced concrete in marine environments, despite the structural implications for coastal infrastructure. The potential of low-pH binders to promote marine organism settlement and support biodiversity on submerged structures is not mentioned, though it broadens the environmental relevance of the materials studied. The Introduction would also benefit from framing service life extension as an environmental strategy in itself: longer-lived structures amortize embodied carbon over more service years, and preserve critical material resources such as aggregates, including sand. Finally, a brief quantitative statement on the economic costs of reinforcement corrosion (the most prevalent cause of premature degradation worldwide) would reinforce the practical urgency of the work.
Several scientific points require deeper treatment. The choice of a CEM II B-LL cement and water-to-cement ratios of 0.5 and 0.6 is not justified; these relatively high w/c ratios may raise durability concerns in aggressive environments and warrant explicit discussion. A quantitative comparison of the CO_2 emissions associated with the two binders would also substantiate the low-carbon framing of the work. The manuscript should more explicitly characterize the concrete properties most relevant to corrosion resistance (pH, electrical resistivity, water-accessible porosity, chloride diffusion coefficient) and clearly explain how these translate into model input parameters, link that is currently underdeveloped. Table 1 would be more informative if it included a measure of temperature variability in addition to mean annual values, and should be explicitly connected to Supplementary Note 10.
The Results and Discussion presents simulation outputs before the model assumptions, boundary conditions, and input parameters have been clearly stated, making it impossible to critically evaluate the findings. This must be corrected regardless of the final structural choice. The authors rely on modeled or generalized environmental data; for several locations, monitoring data from existing maritime studies or high-resolution records from nearby meteorological stations or port authorities could substantially improve boundary condition realism and should be explored. The reference cited in Supplementary Note 10 to support the claim that temperature is not a significant driver does not appear to be retrievable and is not in English, limiting independent verification. The authors should either substitute a verifiable peer-reviewed source or provide targeted simulations demonstrating the model's insensitivity to realistic temperature variations.
The model does not account for biological degradation mechanisms, including bio-corrosion driven by sulfate-reducing bacteria and biofouling communities; this should be acknowledged as a limitation. Finally, although the use of climate projections is commendable, the scenario selection is not justified and no discussion is provided on how uncertainty in climate projections propagates through model outputs. A sensitivity analysis on this point would considerably strengthen the scientific robustness of the work.
In summary, this manuscript makes a valuable contribution to an important and underexplored area of research, and the writing quality is high. The structural inversion of the Methods and Results sections and the absence of a Conclusion and Perspectives section must be corrected as a priority. The Introduction requires significant expansion, and the scientific concerns detailed above (particularly regarding material choice justification, model assumption presentation, temperature effects, and biological degradation) require substantive revision. Upon satisfactory revision, this work has strong potential for publication.

Version 1:

Reviewer comments:

Reviewer #1

(Remarks to the Author)
Thank you for the careful revisions. The paper is solid, and I recommend acceptance.

Reviewer #2

(Remarks to the Author)

The authors have addressed my questions with sufficient data, discussion and elaboration. I believe it is in a proper form for publication.

Reviewer #3

(Remarks to the Author)
Comments adequately addressed in the opinion of this reviewer.

Reviewer #4

(Remarks to the Author)
I reviewed the article that the authors referenced. All of the comments made were carefully incorporated by the authors. I am therefore in favor of publishing the article.

Open Access This Peer Review File is licensed under a Creative Commons Attribution 4.0 International License, which permits use, sharing, adaptation, distribution and reproduction in any medium or format, as long as you give appropriate credit to the original author(s) and the source, provide a link to the Creative Commons license, and indicate if changes were made.
In cases where reviewers are anonymous, credit should be given to 'Anonymous Referee' and the source.
The images or other third party material in this Peer Review File are included in the article's Creative Commons license, unless indicated otherwise in a credit line to the material. If material is not included in the article's Creative Commons license and your intended use is not permitted by statutory regulation or exceeds the permitted use, you will need to obtain permission directly from the copyright holder.
To view a copy of this license, visit https://creativecommons.org/licenses/by/4.0/

NCOMMS-25-76890
Point-by-point response to the reviewers' comments

Text in italics = reviewer comments (reproduced verbatim)
Text in blue = authors' replies

Reviewer #1 (Remarks to the Author):

An interesting paper from he outset.
The group more broadly is globally significant, as they are currently the contemporary critical mass in the domain of advanced studies in the degradation of reinforced concrete structures.
The present study takes the lens (appropriately, given the contemporary importantce) of climate and emissions. This is not a common topic in Nature Comms - in spite of the global relevance. As such, it is important that works such as these are communicated broadly. On this basis, the work is I believe important and merits publication. I have a number of comments for consideration.

Thank you very much for the positive assessment.

1) Writing style: I get the sense when writing for a general journal, the paper has drifted towards colloquial or editorial style, as opposed to focused on the science. Perhaps if the authors are aware that the paper merits publication based on the science, they will be more open to trimming superfluous and non-technical aspects of the narrative. Also, there are several atypical facets in the writing. For example, the Abstract should be one paragraph....not two. The writing style also is better for the reader if words such as 'we' are replaced by different sentence structures that put the subject at the core of the sentence, and not people.
(in fact, the second paragraph in the Abstract, could be the opening part of a consolidated paragraph, as it gives the reader what they need).

We appreciate the suggestions on style. Following consultation with the editor, we have made the following changes:

- We have carefully checked the manuscript for colloquial language and made amendments where necessary.
- We have modified the abstract as suggested (one paragraph, instead of two).
- Concerning the suggestion to replace words such as "we": We acknowledge that preferences for writing style may vary. However, we note that the use of the first-person plural ("we") is a common and accepted practice in Nature journals and does not necessarily detract from the scientific quality or readability of the manuscript. We have therefore retained the "we-form" where it supports clarity and readability, while ensuring that the overall writing remains precise and concise.

2) I appreciate there there is some nuance in the format required for the journal. However, I feel that the introduction provides a lot of background, that is broad, and

then the reader needs to really 'catch up' when the original parts of the paper are emphasised. I believe it's possible to start the paper by critically jumping into the topic at hand.

Given the different reviewers' somewhat conflicting suggestions regarding the introduction (namely, expanding versus trimming the background), we consulted with the editor and adopted a balanced approach that we hope satisfies all concerns.

With respect to the specific reviewer comment here, related to the abrupt transition from background to our original contribution, we have expanded the last part of the introduction (chapter 1), including the addition of an extra figure. These additions help to position our contribution better in the existing literature and state-of-the-art context. As a consequence, and to avoid overlap, we have removed Supplementary Note 2 from the Supplementary Materials file, where initially similar information was presented.

3) The results and discussion are REALLY interesting, and, really important.

Thank you very much for the positive assessment.

At this stage, I could have infinite questions - but I will actually leave the edits to the authors. My main point here, is that the reader is likely to want to be able to grapple with the impactful findings based on what is known in the field already. For example, how do the findings relate to known structures (or models) in the literature (i.e. from other works?). Is there any value in briefly discussing the findings based on the fields knowledge of things like critical chloride thresholds? (and how does this study then impact what may be done by a practitioner?). I am aware the group themselves are leaders in the work around critical chloride thresholds - so are well placed to briefly elaborate the discussion.
Better still, can a visual plot be made that relates the climate focused work in the study, with other works over the recent decades?
I don't see any of the above comments as having a pre-determined correct response or edits. They are principally for the authors to take the perspective of the reader (in the writing) and the revelation of the wider impact (through correlation with known / established works) - in a manner that makes sense to the authors.

We appreciate your perspective and agree that highlighting the broader impact of our finding, especially through connections with established literature and models, will strengthen the manuscript. In response to the above suggestions, we have implemented the following changes in the revised manuscript:

- We have expanded the introduction and discussion of the results accordingly, especially provided more context on existing literature models and how our work relates to this.
- As requested, a visual plot was added (new Figure 1) that positions the work of this study with the state of the art and progress made over the last decades.
- While we have carefully thought about the suggestion to include "critical chloride thresholds" in the discussion, we consider this concept to be beyond

the scope of the present study, primarily due to the different underlying mechanisms. Introducing “critical chloride thresholds” here could risk conflating distinct topics and potentially reduce the clarity of the manuscript. We have therefore chosen not to include this aspect, in order to maintain a clear and focused discussion.

Reviewer #2 (Remarks to the Author):

This paper proposed a conceptual framework to predict the service life of carbonation-influenced reinforced concrete. This novelty is to utilize moisture transport modelling based on day-to-day climate date. Steel rebar corrosion, caused either by carbonation or chloride, is a complicated process, and certain assumptions must be made to provide explainable results. The work has potential impact as it provides a tool to analysis how climate conditions influence corrosion rate. This is beyond the conventional rebar corrosion study which are highly sample specific. Over I feel the work is worth publishing.

Thank you very much for the positive assessment.

I have the following questions that the authors need to address.
1. Homogeneous corrosion along rebar surface is assumed, which is ok for carbonation-induced corrosion. Temperature, as an important climate factor, is not considered to influence the correlation between RH and corrosion rate – how to validate this?

We acknowledge that temperature can affect the corrosion rate of steel. However, as supported by the literature included in Supplementary Note 10, the effect of temperature is clearly secondary compared to that of moisture. Therefore, this study prioritizes the major role of moisture for the prediction of corrosion rates of steel in carbonated concrete. Based on the considerations in Supplementary Note 10, we are of the opinion that the omission of temperature does not significantly influence the outcomes of the present work, and the current formulation is sufficient to convey the main conceptual contribution of this study. Nevertheless, section 3.3 of the manuscript mentions that secondary factors, such as temperature, could be incorporated into the model in future studies.

2. There seems to be an assumption that corrosion rate is only determined by the moisture condition at the vicinity of the steel concrete interface (SCI), i.e. the corrosion rate – moisture correlation is independent of the cover depth. Can this be validated? Would oxygen availability be a limiting factor when rebar is deeply covered?

Since this comment is closely related to comment 11 below, we kindly refer to our reply to comment 11.

With respect to the specific comment on oxygen availability as a potential limiting factor, we would like to mention that previous work[1] focused on this question revealed no effect of oxygen availability under exposure conditions here relevant. We believe that this can also be expected since in carbonation-relevant exposure conditions, gas permeation through the concrete is a critical prerequisite for carbonation to happen. Based on our current understanding, the cover depth primarily influences the corrosion rate through its effect on moisture transport within the concrete, a point that is discussed extensively throughout the

[1] Stefanoni, M., Angst, U. M. and Elsener, B. (2018). "Electrochemistry and capillary condensation theory reveal the mechanism of corrosion in dense porous media." Scientific Reports 8(1): 7407.

manuscript.

3. Isn't the corrosion rate also related to the metallurgical nature of the steel? How is this considered in the model?

Thank you for raising this interesting question. We have carefully considered the potential role of metallurgy and reached the following conclusion. While metallurgical characteristics of reinforcing steel (e.g., composition and microstructure) can influence corrosion, their effect is regarded as secondary compared to that of the surrounding concrete environment, which is the dominant controlling factor (impacting corrosion rates over several orders of magnitude). This assumption is based on the extensive body of existing literature (spanning information from long-term studies of archaeological artefacts in soil, pipe corrosion in soil, to steel in concrete), which indicates that the corrosion rates of iron and carbon steel in near-neutral environments are governed primarily by exposure conditions (electrolyte chemistry and moisture state), rather than by the metallurgical properties of the iron/steel itself[2].

Furthermore, the available literature on metallurgical influences primarily addresses chloride-induced corrosion, where metallurgical features (and local differences) are known to play a role in this form of localized corrosion. For corrosion of steel in carbonated concrete, on the other hand, literature remains limited and is therefore difficult to incorporate in a robust manner at present. Based on corrosion science principles, we expect, however, that the effect of metallurgy may be less pronounced in homogeneous corrosion (as is the case in carbonated concrete), in contrast to localized corrosion (the case in chloride exposure environments).

For these reasons, metallurgical effects were not explicitly considered in this study.

4. In the corrosion kinetics modelling, is the SCI considered to have an identical pore structure as the bulk concrete? There are works suggesting the SCI may have more porosity than bulk concrete (Horne, A.T., Richardson, I.G. and Brydson, R.M.D., 2007. Quantitative analysis of the microstructure of interfaces in steel reinforced concrete. Cement and Concrete Research, 37(12), pp.1613-1623.)

Thank you for this comment. We agree that the steel-concrete interface (SCI) porosity is expected to be higher than that of the bulk cementitious matrix. While explicitly modeling SCI properties might be physically more accurate, it comes with a number of challenges and is currently not deemed feasible (and also not critically needed for the purpose of illustration of our concept). First, accounting for different porosity between SCI and bulk introduces additional complexity to the model, as one would have to incorporate spatially variable material properties. While the numerical implementation itself is relatively straightforward and could be readily achieved in the future, the current bottleneck lies in the limited

[2] An important example of the different literature studies in this regard is Melvin Romanoff's classic work on underground corrosion: Romanoff, M. (1957). Underground corrosion. Washington DC.

availability of data needed to justify assumptions regarding property variability. Second, due to the difficulty in experimentally measuring the degree of saturation (DoS) at the SCI (with its specific sorption isotherms), establishing a relationship between instantaneous corrosion rate (icorr) and DoS at the SCI would be extremely challenging. To ensure the model remains supported by measurable bulk data, we thus consistently applied bulk properties to both (i) the modeled bulk DoS at specific cover depths and (ii) the empirical relationship between bulk DoS and the icorr of steel. In our opinion, this approach is reasonable and allows relating currently available data. We agree, however, that future research should examine the influence of the particular aspect of different porosity at the SCI in more detail.

We have thus updated the manuscript to a) enhance clarity with respect to the selected approach (in the methods section) as well as justifying it better, and b) include a recommendation for future research (in the perspectives section).

5. *I like the idea of ToW for rebars, but what the author stated in line 238-245 seems to suggest that ToW is a misleading measure of the real corrosion kinetics, isn't it?*

Yes, the reviewer understood this correctly. We have explored the concept of Time of Wetness (ToW), as it is a potentially interesting approach, common in other fields such as atmospheric corrosion, where moisture, similar to the present case, is also among the decisive factors for corrosion. The ToW concept works well for atmospheric corrosion because the formation of thin electrolyte films on exposed surfaces increases relatively gradually with relative humidity, allowing corrosion to be approximated using a simple on/off wet–dry threshold. In porous media, however, moisture does not simply thicken an existing surface film, but instead leads to a more complex formation and distribution of electrolyte throughout the pore network. Thus, with increase of moisture, corrosion rate increases highly nonlinearly (exponentially). Because this transition is nonlinear rather than gradual, a binary metric like ToW cannot capture the underlying processes adequately and therefore fails to describe corrosion behavior in porous systems. We have added additional text to the respective part in the manuscript to clarify this better.

6. *Please provide a zoomed-in inset for Fig 4(a). Is it possible to provide the 1st order derivative for Fig 4 in the appendix? This will be a nice representation of corrosion kinetics throughout a year.*

We agree that Figure 4a (now Figure 5a) is difficult to read. While a consistent y-axis scale across all plots in this figure is essential for comparability, we have added a zoomed-in inset to Figure 5a to improve readability as suggested by the reviewer.

The derivative of the data shown in Figure 5 essentially is the data shown in Figure 2e,f and all corresponding figures shown in Supplementary Note 7. The only difference is the unit: While Figure 2e,f and the corresponding figures in Supplementary Note 7 are given in terms of $\mu A/cm^2$, Figure 5 displays the data in

terms of μm, which is the integrated form of electrochemical corrosion rate converted by Faraday's law of electrolysis to $\frac{\mu m}{y}$.

7. Fig 5 is great, but the estimation of carbonation phase relies on Duracrete model which is not an original work of this paper. Duracrete model itself has a good number of assumptions and regressions. Can the author comment on whether Duracrete model improves or reduces the reliability of service-life prediction in this paper? My concern is that the Duracrete model could not capture the influence of daily moisture change as the propagation-phase model in this paper.

Thank you for the positive comment on Figure 5. The use of the DuraCrete model for carbonation depth estimation is indeed based on an established framework; this model is widely regarded as state-of-the-art in probabilistic service-life modeling. We would like to point out that the DuraCrete model already incorporates key environmental influences, including temporal moisture changes through the time-of-wetness (ToW) concept. With respect to the specific influence of short-term or daily moisture fluctuations, however, the current literature remains limited, with comparatively little experimental evidence supporting the effect of fine-scale daily RH or wetting-drying variations on carbonation progression. Existing models such as the DuraCrete model are generally calibrated on long-term averaged environmental conditions. For these reasons, we believe that the use of the DuraCrete model for describing the corrosion initiation phase is well justified.

Finally, we would like to mention that the focus of this study is on improving the modelling of the propagation stage of reinforcement corrosion, rather than revisiting the comparatively well-established carbonation initiation framework. Therefore, the use of the DuraCrete model does not reduce the reliability of the overall service-life prediction in this work. Instead, it provides a consistent and widely accepted baseline for the initiation phase, allowing the study to concentrate on advancing the subsequent propagation modelling.

8. The current practical approaches indeed oversimplify the exposure conditions, but are friendly to normal concrete technicians. The author must explain how the proposed computational modelling method, which requires concrete property and climate data (daily based, not yearly averaged as the author mentioned) as input, can be readily used by the industry.

Thank you for raising this point. We agree that it will be important, ultimately, to have models that can be used by industry. However, the key idea of this manuscript is to propose a guiding concept for future research and engineering development, aiming to promote a shift in the philosophy with which the durability of reinforced concrete is addressed and to stimulate further discussion. As such, our concept is not intended for direct application in practice. We have stressed this in the "perspectives" section of the "results and discussion" chapter.

9. I fully agree with this: "Thus, climate-change induced shifts in precipitation patterns

may pose a problem and may threaten the durability of eco-friendly, reinforced concrete structures much more than a temperature rise by 2-3 °C."

Thank you very much for your positive comment. We agree that this is an important finding, and we are pleased to hear that you share this view.

10. Since supplementary figure 6-8 and table 2 are from real measurement, the author should provide the mix design of the specimens.

A Supplementary Note (no. 3) and table were added to report the mix design and properties of studied materials in agreement with the request of the reviewer.

11. The correlation between saturation and corrosion rate is a core input to the overall modelling. In section 4.3, I understand that a simplification is needed to complete this conceptual framework. However, the author should provide stronger justification to this relationship. In particular, why is this relationship independent of the cover depth? The author should also provide a perspective how this can be validated for a random concrete mixture in real practise.

We agree with the reviewer that the relationship between concrete saturation and steel corrosion rate is crucial for the future application of the proposed framework in both science and engineering. Accordingly, we have expanded the discussion of this relationship in the revised manuscript (Section 3.3), highlighting the limitations of currently available experimental data and providing specific perspectives and guidance for future research in this area (e.g. for validation, as suggested by the reviewer).

The relationship adopted to illustrate the here proposed concept is based on the best available data at present; however, we acknowledge that uncertainties remain in these data due to the limitations in the literature mentioned above and described in more detail in the revised paper. These uncertainties, and their implications for the presented calculations, have now been more explicitly discussed and highlighted in the revised manuscript.

We trust that these amendments satisfactorily address the reviewer's concern.

Reviewer #3 (Remarks to the Author):

This study presented a framework for durability evaluation of concrete subjected to carbonation. The study is well written and relevant to current needs.

Thank you very much for the positive assessment.

However, there some aspects that are unclear and should be amended/added. Please see points below:

- L40: silica fume? That's very seldom used for clinker replacement and specifically used for durability / high performance concrete

We agree with the reviewer that silica fume is rarely used for concrete in carbonation exposure environments. We have removed this from the list in the introduction, and instead added "combustion ashes from biomass and waste" and specified "slag" as "ground granulated blast furnace slag".

- L42: I would not call these properties superior. Early-age heat of hydration reduction is beneficial for massive concrete structures but comes at a penalty of lower strength, which admittedly is a main blocker in the adoption of lower carbon concretes. Chloride diffusion and associated properties are more superior for SCMs, as is later age strength gain

We agree with the reviewer that reduction of early-age heat of hydration may not only be viewed as an advantage (decreased cracking), but also introduce a disadvantage (lower early age strength, limiting the adoption of such concretes in practice). We have thus removed this aspect from the sentence and rephrased it. The main point of this sentence is that low-clinker cements can produce concrete with properties comparable to those of traditional cements, or in some aspects, even superior ones. We hope that our revision clarified this.

- L54-56: reads like depassivation is the main reason the carbon steel is used

We agree with the reviewer that the original sentence may have been misleading in its phrasing, and we have revised it for improved clarity.

- L79: abandon is a strong word. I guess "relax the reliance" on carbonation front reaching the reinforcement for durability might be more straight forward

Thank you for the suggestion, which we have adopted.

- L92-95: simply, the deemed-to-satisfy approach

Thank you for suggestion this established term. We have added it to improve clarity.

- L96: UK too from BSI flex 350

Thank you for the suggestion. This standard has now been included in the overview table in Supplementary Note 1 as well.

- Supplementary information Table 1, for UK, "E4" is not a cement but a performance category. Suggest you list the E4 corresponding cements. Also UK has BSI Flex 350. Suggest the authors also consider, Ireland, Norway and Canada in the review.

Thank you for noticing this point on the British standards. We have clarified the corresponding information in Table 1 and, as suggested by the reviewer, incorporated information on carbonation-induced corrosion measures in standards from additional countries.

L116. Agree that these are qualitative. But there are quantifications based on RH. Such as, works by Stefanie Von Greve-Dierfeld and other organisations etc. These need to be reported and acknowledged

Thank you for raising this point. We agree that attempts have been made in the literature to quantify RH and other measures (temperature, ToW) in the context of natural carbonation (e.g. field exposure sites). We have now mentioned these previous works in the introduction of the revised manuscript as requested by the reviewer.

- L146: CEM II/B-LL is not a composite cement but a Portland-limestone cement. Also declare the % limestone power and the two distinct w/b

Thank you for bringing this to our attention. We carefully checked the relevant European standards (EN 197-1). While the term "Portland composite cement" is more general, we agree that "Portland limestone cement" is more appropriate for the CEM II/B-LL used in this study. We made amendments in the manuscript accordingly.

Regarding the % limestone and w/b ratios, Supplementary Note 3 was added to report the mix design and further details of studied materials.

- The basis on selection CEM I and CEM IIB LL for calculations need to be clarified.

We agree that justification of the selection of these systems would have been beneficial. We have now added such information to section 4.2 of the revised manuscript.

- L192: CEM II/B-LL on its own is very hard to use in concrete at higher %, the w/b of 0.6 will practically make this concrete unusable and really vulnerable to deterioration.

We agree with the reviewer that a CEM II/B-LL concrete with a w/b ratio of 0.6 is expected to be relatively vulnerable to deterioration. This mixture was intentionally selected as an extreme case to highlight these effects. Hence, our results are

consistent with the reviewer's remark, as they indeed indicate pronounced susceptibility to deterioration for this mix.

- Figure 2: useful finding, but how reliable is the estimation?

The moisture transport model employed in this study represents the current state of the art for simulating moisture transport in cementitious materials and has been extensively validated in the literature[3]. For instance, it was shown that upon calibration with measured mass loss data, this model was able to accurately reproduce saturation (or moisture content) profiles, which were independently validated using the gamma-ray attenuation technique[4]. Here, the model was calibrated by laboratory-measured water absorption data (see Supplementary Notes 4 and 6). Therefore, the moisture transport simulations presented in this work are considered robust and sufficiently accurate for the intended analysis. The results of the moisture transport simulations were then converted to corrosion rates on the basis of available literature data, as detailed in Supplementary Note 8 and discussed in the revised manuscript in more detail than in the original version in the manuscript. Specifically, to provide more context on the reliability of these calculations, we have expanded the discussion in the revised manuscript (Section 3.3), highlighting the limitations of currently available experimental data and providing specific perspectives and guidance for future research in this area (e.g. for validation, as suggested by the reviewer).

- Figure 4: as in Figure 2 the relevant threshold for negligible corrosion rate is proposed as 0.1 µA/cm^2, it would be useful to have an equivalent for section loss vs crack (acknowledging this depends on concrete strength, cover depth etc)

We agree with the reviewer that such thresholds are needed both for corrosion rate and for sectional loss. We have thus expanded the manuscript accordingly and provided more background for the sectional loss threshold (including acknowledging literature and uncertainties in our assumption) in section 3.2.

- Finally on perspectives, what should be more clearly discussed is the lack of on-site validation of the model. Have the authors tried to corelate their modelling results to evidence/measurements from actual structures in corresponding locations? This type of analysis is something that should be added to strengthen the validity of the approach.

We agree with the reviewer that on-site validation of the model is an important aspect. Thus, the aspect of validation has been significantly strengthened in the revised manuscript. Specifically, we have differentiated between the carbonation and corrosion components of the proposed model. As the carbonation component – here, the DuraCrete model – is already well-established in the literature, we have integrated references to existing validation studies within the introduction of the manuscript to provide better context. The moisture-governed corrosion

[3] Mainguy, M., Coussy, O. and Baroghel-Bouny, V., 2001. Role of air pressure in drying of weakly permeable materials. Journal of engineering mechanics, 127(6), pp.582-592.
[4] Zhang, Z., Thiery, M. and Baroghel-Bouny, V., 2015. Numerical modelling of moisture transfers with hysteresis within cementitious materials: Verification and investigation of the effects of repeated wetting–drying boundary conditions. Cement and Concrete Research, 68, pp.10-23.

component, on the other hand, represents a novel frontier where empirical validation remains an ongoing challenge. We have explicitly addressed this in the "Perspectives" (section 3.3), where we now highlight – among other aspects – the need for validating laboratory-derived data against long-term field data. This expansion clarifies both the current scientific boundaries of the model and the specific pathways required for future empirical verification. We hope that this adequately addresses the reviewer's concern.

- Good study overall and moves durability design/characterisation towards the right direction.

Thank you very much for the positive comment.

Reviewer #4 (Remarks to the Author):

This manuscript addresses a timely and relevant challenge at the intersection of sustainable construction and infrastructure durability, proposing a climate-informed corrosion modeling framework for low-carbon concretes (LCCs). The writing is clear and the topic is of undeniable significance.

Thank you very much for the positive assessment.

However, several structural issues and scientific concerns must be resolved before publication can be recommended.
The most critical structural issue is the unconventional placement of the Methods section after Results and Discussion. This impairs the reader's ability to critically evaluate the modeling approach, input parameters, and simulation limitations. Equally important, the manuscript lacks both a Conclusion and a Perspectives section, leaving the reader without any synthesis of findings, critical appraisal of limitations, or indication of future directions.

We appreciate these suggestions on manuscript structure and style. Following consultation with the editor, we provide the following replies:

- It is common in Nature portfolio journals to place the "Methods" after the "Results and Discussion". While we can thus not change this, we have carefully checked the manuscript and made sure (in response to the reviewer's concern) that the rationale of the methodological approach is outlined in section 2 as well as in the beginning of section 3. Hence, the reader should now have sufficient knowledge about the methodological approach when the results are presented in section 3.
- Similarly, Nature Communications aims to avoid a "Conclusion" section. Nevertheless, we have revisited the manuscript to ensure that a concluding discussion as well as perspectives are integrated towards the end.

The Introduction requires substantial expansion. While it effectively contextualizes climate change and the need to reduce the carbon footprint of construction materials, several important dimensions are absent. No connection is drawn between global sea level rise and reinforced concrete in marine environments, despite the structural implications for coastal infrastructure. The potential of low-pH binders to promote marine organism settlement and support biodiversity on submerged structures is not mentioned, though it broadens the environmental relevance of the materials studied. The Introduction would also benefit from framing service life extension as an environmental strategy in itself: longer-lived structures amortize embodied carbon over more service years, and preserve critical material resources such as aggregates, including sand. Finally, a brief quantitative statement on the economic costs of reinforcement corrosion (the most prevalent cause of premature degradation worldwide) would reinforce the practical urgency of the work.

Given the different reviewers' somewhat conflicting suggestions regarding the introduction (namely, expanding versus trimming the background), we consulted with the editor and adopted a balanced approach that we hope satisfies all

concerns. With respect to the specific reviewer comment here, we have made the following changes:

- Concerning the suggestion to include a brief statement on the economic costs of corrosion of steel in concrete, we agree with the reviewer and have added a statement to the introduction, including making reference to the related literature.
- Similarly, we have added statements addressing the point that service life extension can be an "environmental strategy" as well (both in the introduction section as well as in the perspectives section).
- Concerning the suggestion to mention global sea level rise and other aspects related to marine exposure, we would like to note that this manuscript has the focus on corrosion in carbonation environments. This is a specific degradation mechanism distinct from other mechanisms such as chloride-induced corrosion relevant in marine exposure. Accordingly, the field has traditionally treated these mechanisms separately. Consistent with this established distinction, the present study focuses exclusively on carbonation-induced corrosion, with particular emphasis on the rate at which corrosion develops. We have made this clear in the introduction.

Several scientific points require deeper treatment. The choice of a CEM II B-LL cement and water-to-cement ratios of 0.5 and 0.6 is not justified; these relatively high w/c ratios may raise durability concerns in aggressive environments and warrant explicit discussion.

We agree with the reviewer that the selection of materials could have been better justified. In the revised manuscript, we have now provided such justification (section 4.2). We further agree with the reviewer that the system "CEM II/B-LL concrete with a w/b ratio of 0.6" is expected to be relatively vulnerable to deterioration. This mixture was intentionally selected as an extreme case to highlight the effects under study in this manuscript. Hence, our results are consistent with the reviewer's remark, as they indeed indicate pronounced susceptibility to deterioration for this mix.

A quantitative comparison of the CO_2 emissions associated with the two binders would also substantiate the low-carbon framing of the work.

Thank you for this interesting suggestion. We have added a note on the global warming potential (equivalent CO2 emissions) of the different mixes in section 3.1 of the revised manuscript, highlighting the reduced emissions of concretes produced with CEM II B-LL cement compared to the CEM I concrete. This new information, added here in the context of service life predictions, indicates that equally durable concrete can be achieved while reducing the greenhouse gas emissions.

The manuscript should more explicitly characterize the concrete properties most relevant to corrosion resistance (pH, electrical resistivity, water-accessible porosity,

chloride diffusion coefficient) and clearly explain how these translate into model input parameters, link that is currently underdeveloped.

We agree with the reviewer that a clearer and more comprehensive description of the material properties would have been beneficial. In response to the reviewer's request, we have thus expanded Supplementary Notes 3–6 to include additional material properties obtained from characterization. Furthermore, we have carefully reviewed Section 4 of the manuscript to ensure that the link between material characterization and the corresponding model input parameters is clearly described.

Table 1 would be more informative if it included a measure of temperature variability in addition to mean annual values, and should be explicitly connected to Supplementary Note 10.

More detailed information on the variability of temperature in the four studied locations was added to the supplementary information (specifically, to Supplementary Note 2, where a detailed analysis of the meteorological data is presented). Moreover, as suggested by the reviewer, a link to Supplementary Note 10 was established.

The Results and Discussion presents simulation outputs before the model assumptions, boundary conditions, and input parameters have been clearly stated, making it impossible to critically evaluate the findings. This must be corrected regardless of the final structural choice.

We kindly refer to the comment above related to the structure of the manuscript determined by the journal. To mitigate the reviewer's concern, we have carefully checked that the essential key information on methodology is given prior to the presentation of the results and that the reader is adequately directed to all needed details; we have also added more methodological details to the figure caption to better support the interpretation of the data.

The authors rely on modeled or generalized environmental data; for several locations, monitoring data from existing maritime studies or high-resolution records from nearby meteorological stations or port authorities could substantially improve boundary condition realism and should be explored.

We would like to clarify that we did not rely on "modeled or generalized environmental data", but instead used publicly available, real meteorological data from weather stations, as suggested by the reviewer. Recognizing that this may not have been adequately described in the original version, we have carefully revised the manuscript and the supplementary materials to make this point clearer.

The reference cited in Supplementary Note 10 to support the claim that temperature is not a significant driver does not appear to be retrievable and is not in English, limiting independent verification. The authors should either substitute a verifiable peer-reviewed

source or provide targeted simulations demonstrating the model's insensitivity to realistic temperature variations.

Thank you for noticing this point. Although the title of study mentioned by the reviewer is in German, the report content is actually available in English. We recognize, however, that locating the report online may not have been straightforward. To improve accessibility of this reference, we have now added a direct link in Supplementary Note 10. Furthermore, as requested by the reviewer, Supplementary Note 10 was expanded to include additional literature (peer reviewed studies) on the effect of temperature on the corrosion rate of steel compared to that of moisture.

The model does not account for biological degradation mechanisms, including bio-corrosion driven by sulfate-reducing bacteria and biofouling communities; this should be acknowledged as a limitation.

We agree that the model does not account for such degradation mechanisms. In agreement with the reviewer's request, we have added a statement describing the limitation in the introduction.

Finally, although the use of climate projections is commendable, the scenario selection is not justified and no discussion is provided on how uncertainty in climate projections propagates through model outputs. A sensitivity analysis on this point would considerably strengthen the scientific robustness of the work.

We agree that scenario selection and the propagation of uncertainties in climate projections are important aspects when assessing future performance. However, the primary aim of this study is to demonstrate how a given set of meteorological input data can be integrated into the proposed framework to predict corrosion-induced damage. For this purpose, we relied on existing meteorological datasets as illustrative inputs rather than exploring or comparing specific climate scenarios. With respect to the selection of the meteorological data, we have carefully checked the manuscript and, where necessary, clarified the presentation to ensure that it is sufficiently justified.

The potential use of the proposed framework to investigate the effects of future climate change (such as shifts in precipitation patterns) is explicitly presented as an outlook in the perspectives section of the manuscript, meant to guide and stimulate future research. In this regard, we acknowledge that a systematic treatment of uncertainties and a dedicated sensitivity analysis would be essential for such future-oriented applications. This, however, is beyond the scope of the present work.

In summary, this manuscript makes a valuable contribution to an important and underexplored area of research, and the writing quality is high.

Thank you very much for the positive assessment.

The structural inversion of the Methods and Results sections and the absence of a Conclusion and Perspectives section must be corrected as a priority. The Introduction requires significant expansion, and the scientific concerns detailed above (particularly regarding material choice justification, model assumption presentation, temperature effects, and biological degradation) require substantive revision.

We kindly refer to our detailed individual replies to these points above.

Upon satisfactory revision, this work has strong potential for publication.

Thank you very much for the positive assessment.

NCOMMS-25-76890A
Point-by-point response to the reviewers' comments

Text in italics = reviewer comments (reproduced verbatim)
Text in blue = authors' replies

REVIEWERS' COMMENTS

Reviewer #1 (Remarks to the Author):

Thank you for the careful revisions. The paper is solid, and I recommend acceptance.

Authors' reply: Thank you very much for the positive assessment.

Reviewer #2 (Remarks to the Author):

The authors have addressed my questions with sufficient data, discussion and elaboration. I believe it is in a proper form for publication.

Authors' reply: Thank you very much for the positive assessment.

Reviewer #3 (Remarks to the Author):

Comments adequately addressed in the opinion of this reviewer.
Authors' reply: Thank you very much for the positive assessment.

Reviewer #4 (Remarks to the Author):

I reviewed the article that the authors referenced. All of the comments made were carefully incorporated by the authors. I am therefore in favor of publishing the article.

Authors' reply: Thank you very much for the positive assessment.
